# Supplementary figures and images for: High numbers of COVID-19 patients transit through non-COVID wards, and associated healthcare workers have high infection rates: An observational cross-sectional study
Source: PLoS One. 2022 Oct 19;17(10):e0275154. doi: 10.1371/journal.pone.0275154 (PMC9581418; doi:10.1371/journal.pone.0275154)

# Supplement table 1


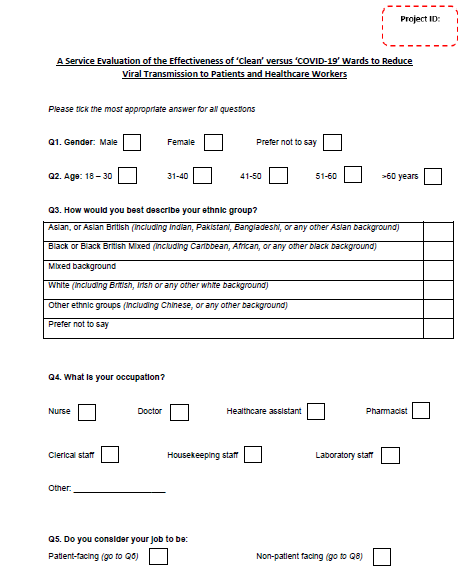


#
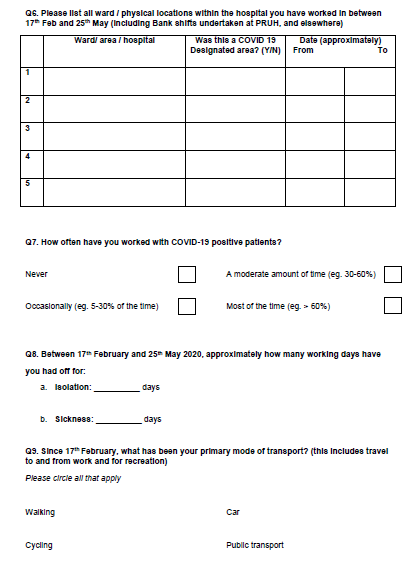


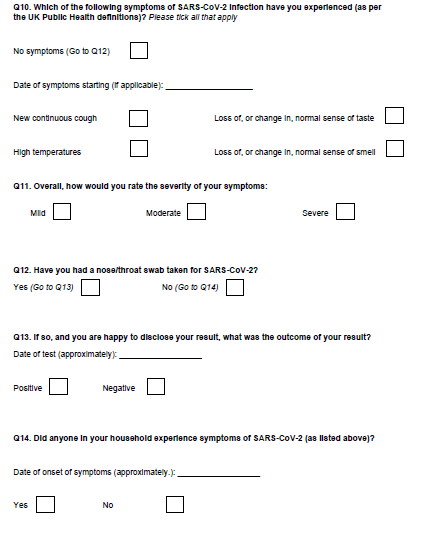


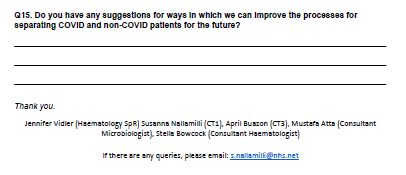


***S1 Table****:* Questionnaire completed by participants

Supplement: S1 Table — (DOCX) [file pone.0275154.s001.docx]
