# Supplementary material for: High numbers of COVID-19 patients transit through non-COVID wards, and associated healthcare workers have high infection rates: An observational cross-sectional study
Source: PLoS One. 2022 Oct 19;17(10):e0275154. doi: 10.1371/journal.pone.0275154 (PMC9581418; doi:10.1371/journal.pone.0275154)
Supplement: S2 Table — Values are all n (%) with p-values from chi-square tests. NPF–non patient-facing, PF–patient-facing. (DOCX) [file pone.0275154.s002.docx]

# Supplement table 2

|  |  | Total | negative/indeterminate | positive | P value |  |
| --- | --- | --- | --- | --- | --- | --- |
|  |  | N=1,118 (%) | N=733 (65.6%) | N=385 (34.4%) |  |  |
| Age | 18-30 | 295 | 191 (64.7) | 104 (35.3) | 0.068 |  |
|  | 31-40 | 256 | 164 (64.1) | 92 (35.9) |  |  |
|  | 41-50 | 260 | 163 (62.7) | 97 (37.3) |  |  |
|  | 51-60 | 232 | 154 (66.4) | 78 (33.6) |  |  |
|  | >60 | 71 | 57 (80.3) | 14 (19.7) |  |  |
|  | unknown | 4 | 4 (100.0) | 0 (0.0) |  |  |
| Gender | male | 233 | 151 (64.8) | 82 (35.2) | 0.15 |  |
|  | female | 878 | 575 (65.5) | 303 (34.5) |  |  |
|  | unknown | 7 | 7 (100.0) | 0 (0.0) |  |  |
| Ethnicity | white | 592 | 440 (74.3) | 152 (25.7) | <0.001 |  |
|  | black | 118 | 64 (54.2) | 54 (45.8) |  |  |
|  | asian | 315 | 171 (54.3) | 144 (45.7) |  |  |
|  | mixed | 24 | 17 (70.8) | 7 (29.2) |  |  |
|  | other | 37 | 19 (51.4) | 18 (48.6) |  |  |
|  | unknown | 32 | 22 (68.8) | 10 (31.2) |  |  |
| BAME | white/unknown | 624 | 462 (74.0) | 162 (26.0) | <0.001 |  |
|  | BAME | 494 | 271 (54.9) | 223 (45.1) |  |  |
| Public transport | no | 740 | 508 (68.6) | 232 (31.4) | 0.002 |  |
|  | yes | 378 | 225 (59.5) | 153 (40.5) |  |  |
| Risk allocation | Laboratory (NPF) | 74 | 66 (89.2) | 8 (10.8) | <0.001 |  |
|  | Non-clinical hospital staff (NPF) | 220 | 169 (76.8) | 51 (23.2) |  |  |
|  | Non-COVID wards only (PF) | 286 | 196 (68.5) | 90 (31.5) |  |  |
|  | Mixed exposure (PF) | 346 | 194 (56.1) | 152 (43.9) |  |  |
|  | COVID wards throughout (PF) | 168 | 94 (56.0) | 74 (44.0) |  |  |
|  | Patient facing - Unknown | 24 | 14 (58.3) | 10 (41.7) |  |  |
| Symptomatic household contacts |  |  |  |  |  |  |
|  | No | 718 | 517 (72.0) | 201 (28.0) | 0.25 |  |
|  | Yes | 350 | 185 (52.9) | 165 (47.1) |  |  |
|  | Unknown | 50 | 31 (62.0) | 19 (38.0) |  |  |
|  |  |  |  |  |  |  |
| Self assessed COVID patient contact | Never | 276 | 217 (78.6) | 59 (21.4) |  | <0.001 |
|  | Occasionally | 283 | 179 (63.3) | 104 (36.7) |  |  |
|  | Moderate | 192 | 100 (52.1) | 92 (47.9) |  |  |
|  | Most | 225 | 122 (54.2) | 103 (45.8) |  |  |
|  | Unknown | 142 | 115 (81.0) | 27 (19.0) |  |  |
|  |  |  |  |  |  |  |
| Days of sickness |  | Median: 0  IQR: 5 (0 – 5) | Median: 0  IQR: 0 (0 – 0) | Median: 2  IQR: 10 ( 0 – 10) | P=0.52 |  |
|  |  |  |  |  |  |  |
| Severity of symptoms |  |  |  |  |  |  |
|  | Mild | 184 | 93 (50.5) | 91 (49.5) | P=0.81 |  |
|  | Moderate | 227 | 91 (40.1) | 136 (59.9) |  |  |
|  | Severe | 51 | 10 (19.6) | 41 (80.4) |  |  |
|  | Unknown | 17 | 11 (64.7) | 6 (35.3) |  |  |
|  | (No symptoms) | 639 | 528 (82.6) | 111 (17.4) |  |  |

***S2 table***: Demographics of all 1118 staff by evidence of infection (positive serology or positive PCR versus negative for both tests). Values are all n (%) with p-values from chi-square tests.

*NPF – non patient-facing, PF – patient-facing*
