# Supplementary material for: High numbers of COVID-19 patients transit through non-COVID wards, and associated healthcare workers have high infection rates: An observational cross-sectional study
Source: PLoS One. 2022 Oct 19;17(10):e0275154. doi: 10.1371/journal.pone.0275154 (PMC9581418; doi:10.1371/journal.pone.0275154)
Supplement: S3 Table — Values are all n (%) with p-values from chi-square tests. (DOCX) [file pone.0275154.s003.docx]

# Supplement table 3

|  |  | Total  n = 1118 (%) | Patient facing n = 824 (73.7%) | Non-patient facing (%) (n = 294) (26.3%) |
| --- | --- | --- | --- | --- |
| Gender |  |  |  |  |
|  | Male | 233 | 163 (70.0) | 70 (30.0) |
|  | Female | 878 | 655 (74.6) | 223 (254) |
|  | Unknown | 7 | 6 (85.7) | 1 (14.3) |
|  |  |  |  |  |
|  |  |  |  |  |
|  |  |  |  |  |
| Age (years) |  |  |  |  |
|  | 18 – 30 | 295 | 239 (81.0) | 56 (19.0) |
|  | 31 – 40 | 256 | 197 (77.0) | 59 (23.0) |
|  | 41 – 50 | 260 | 200 (76.9) | 60 (23.1) |
|  | 51 – 60 | 232 | 144 (62.1) | 88 (37.9) |
|  | > 60 | 71 | 41 (57.7) | 30 (42.3) |
|  | Unknown | 4 | 3 (75.0) | 1 (25.0) |
|  |  |  |  |  |
|  |  |  |  |  |
| Ethnicity |  |  |  |  |
|  | Asian | 315 | 282 (89.5) | 33 (10.5) |
|  | Black | 118 | 91 (77.1) | 27 (22.9) |
|  | White | 592 | 374 (63.2) | 218 (36.8) |
|  | Mixed | 24 | 16 (66.7) | 8 (33.3) |
|  | Other | 37 | 34 (91.9) | 3 (8.1) |
|  | Unknown | 32 | 27 (84.4) | 5 (15.6) |
|  |  |  |  |  |
| Occupation |  |  |  |  |
|  | Nurses & nursing assistants | 467 | 461 (98.7) | 6 (1.3) |
|  | Doctors | 127 | 125 (98.4) | 2 (1.6) |
|  | Clerical staff | 219 | 62 (28.3) | 157 (71.7) |
|  | Laboratory staff | 69 | 0 (0.0) | 69 (100.0) |
|  | Pharmacist | 41 | 32 (78.0) | 9 (22.0) |
|  | Midwife | 28 | 28 (100.) | 0 (0.0) |
|  | Housekeeping staff | 21 | 19 (90.5) | 2 (9.5) |
|  | Allied health professions (PT + OT + SALT + radiographers) | 43 | 43 (100.0) | 0 (0.0) |
|  | Porters | 13 | 12 (92.3) | 1 (7.7) |
|  | Retail | 8 | 0 (0.0) | 8 (100.0) |
|  | Engineers | 7 | 1 (14.3) | 6 (85.7) |
|  | Other | 75 | 41 (54.7) | 34 (45.3) |
|  |  |  |  |  |
| Public transport used |  |  |  |  |
|  | No | 726 | 499 (68.7) | 227 (31.3) |
|  | Yes | 378 | 315 (83.3) | 63 (16.7) |
|  | Unknown | 14 | 10 (71.4) | 4 (28.6) |
|  |  |  |  |  |
| COVID symptoms |  |  |  |  |
|  | No | 573 | 390 (68.1) | 183 (31.9) |
|  | Yes | 453 | 371 (81.9) | 82 (18.1) |
|  | Other symptoms noted | 26 | 22 (84.6) | 4 (15.4) |
|  | Unknown | 66 | 41 (62.1) | 25 (37.9) |
|  |  |  |  |  |
| Severity of symptoms |  |  |  |  |
|  | Mild | 184 | 152 (82.6) | 32 (17.4) |
|  | Moderate | 227 | 187 (82.4) | 40 (17.6) |
|  | Severe | 51 | 43 (84.3) | 8 (15.7) |
|  |  |  |  |  |
| Days of sickness |  |  |  |  |
|  |  |  | Median: 0  Mean:4.5  Range: 0-120 | Median: 0  Mean: 3.2  Range: 0-63 |
|  | Unknown | 75 | 45 | 30 |
|  |  |  |  |  |
| Symptomatic household contacts |  |  |  |  |
|  | No | 718 | 517 (72.0) | 201 (28.0) |
|  | Yes | 350 | 277 (79.1) | 73 (20.9) |
|  | Unknown | 50 | 30 (60.0) | 20 (40.0) |
|  |  |  |  |  |
| Swab taken? |  |  |  |  |
|  | Yes - positive | 43 | 39 (90.7) | 4 (9.3) |
|  | Yes - negative | 150 | 110 (73.3) | 40 (26.7) |
|  | No | 212 | 172 (81.1) | 40 (18.9) |
|  | Unknown | 713 | 503 (70.5) | 210 (29.5) |
|  |  |  |  |  |

***S3Table****:* Demographics of all 1118 staff by role (patient-facing or non patient-facing). Values are all n (%) with p-values from chi-square tests.
