# Supplementary material for: High numbers of COVID-19 patients transit through non-COVID wards, and associated healthcare workers have high infection rates: An observational cross-sectional study
Source: PLoS One. 2022 Oct 19;17(10):e0275154. doi: 10.1371/journal.pone.0275154 (PMC9581418; doi:10.1371/journal.pone.0275154)
Supplement: S4 Table — *Staff were not specifically asked about PPE but these were free-text responses to the above question. (DOCX) [file pone.0275154.s004.docx]

# Supplementary table 4

| **Response Themes** | **Percentage of Responses** | **Areas for Improvement Highlighted** |
| --- | --- | --- |
| Isolation Procedures/ ward allocation/ patient flow | 42% | Separation of COVID /non-COVID patients/whole hospital floors/separate lifts  Use of side rooms  Initial ward allocations  Internal ward transfers |
| Testing | 37% | Timing of testing patients  Reliability and speed of testing and results  Staff testing |
| Reduction of Transmission | 12% | Housekeeping  Staffing issues e.g. not moving between ward types Working from home  Visiting policy  Social distancing in hospital |
| *PPE | 9% | PPE for staff  PPE for visitors and patients |

***S4 Table:*** Themes in staff responses to the question ‘Do you have any suggestions for ways in which we can improve the processes for separating COVID and non-COVID patients for the future?’

*Staff were not specifically asked about PPE but these were free-text responses to the above question.
