# Supplementary material for: High numbers of COVID-19 patients transit through non-COVID wards, and associated healthcare workers have high infection rates: An observational cross-sectional study
Source: PLoS One. 2022 Oct 19;17(10):e0275154. doi: 10.1371/journal.pone.0275154 (PMC9581418; doi:10.1371/journal.pone.0275154)
Supplement: S1 Fig — (DOCX) [file pone.0275154.s005.docx]

# Supplementary figure 1


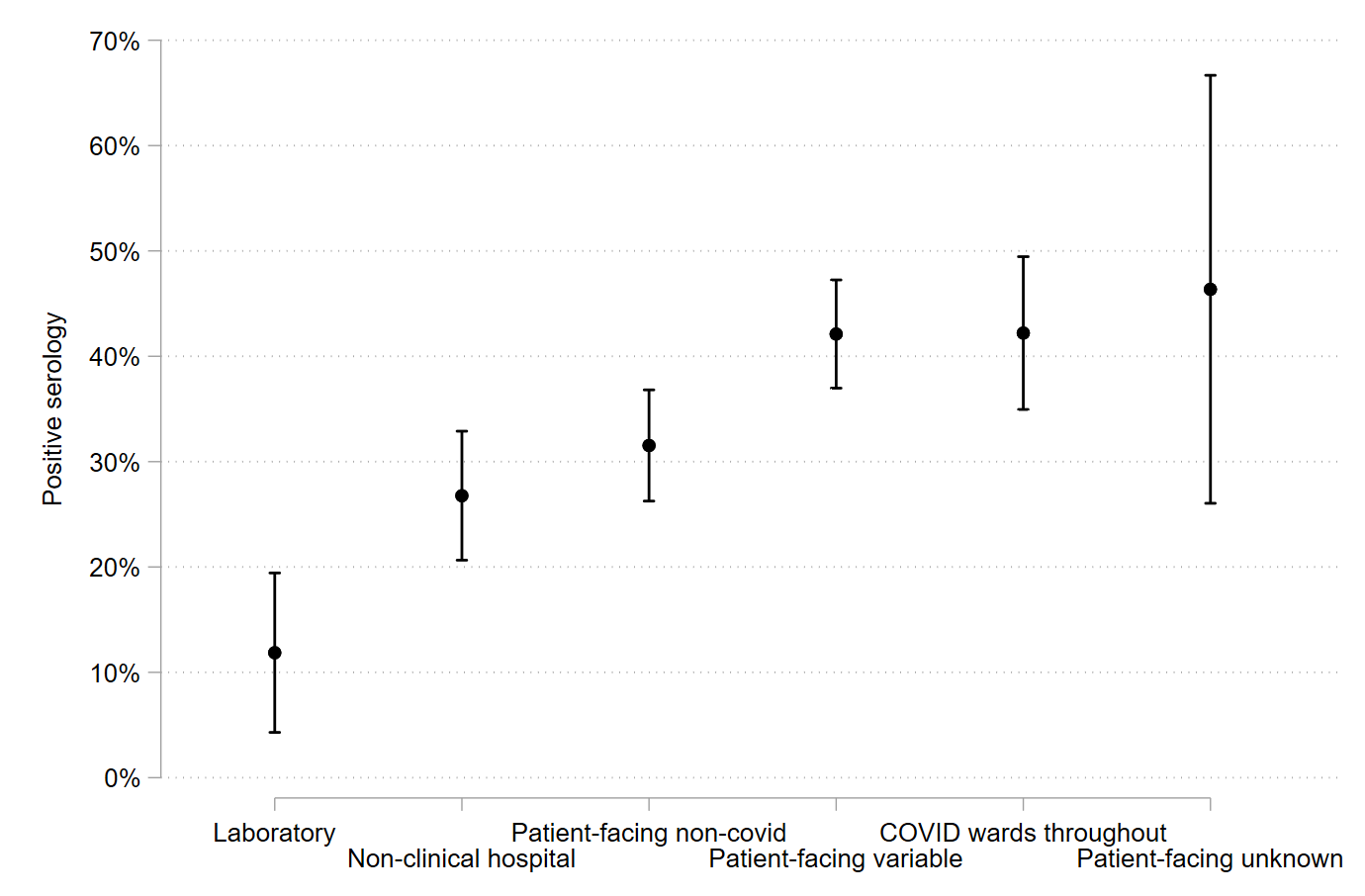


***S1 Figure****:* Adjusted predicted probabilities of infection for all risk exposure categories where these are adjusted for age, gender, BAME, public transport and household contacts
