# Supplementary material for: High numbers of COVID-19 patients transit through non-COVID wards, and associated healthcare workers have high infection rates: An observational cross-sectional study
Source: PLoS One. 2022 Oct 19;17(10):e0275154. doi: 10.1371/journal.pone.0275154 (PMC9581418; doi:10.1371/journal.pone.0275154)
Supplement: S1 Dataset — (PDF) [file pone.0275154.s006.pdf]

ID number	Gender	Age (years)	Ethnicity	Revised risk allocation	Days off for		Mode of transport				Symptoms					Severity of symptoms	Serology result	PCR Result	Overall result	Household symptoms
			Ethnicity		Isolation	Sickness	Walking	Cycling	Car	Public transport	None	Cough	Fever	No taste	Anosmia					Household symptoms
001	Female	51-60	White	Patient-facing: COVID wards throughout	0	0	1	0	0	0	1	0	0	0	0	n/a	Negative	Negative	Negative	Yes
002	Female	41-50	White	Patient-facing: Mixed exposure	0	0	0	0	0	1	1	0	0	0	0	n/a	Negative	Not done	Negative	No
004	Female	31-40	Asian	Patient-facing: Mixed exposure	7	0	0	0	1	0	0	0	1	0	0	Moderate	Negative	Not done	Negative	Yes
005	Female	18 – 30	Asian	Patient-facing: COVID wards throughout	0	18	0	0	0	1	0	0	1	1	1	Moderate	Positive	Positive	Positive	Yes
006	Male	51-60	Asian	Patient-facing: Non-COVID wards only	0	7	0	0	1	0	0	0	1	0	0	Mild	Negative	Negative	Negative	No
007	Female	18 – 30	Asian	Patient-facing: COVID wards throughout	0	0	0	0	1	0	0	1	0	0	0	Moderate	Negative	Not done	Negative	Yes
008	Female	18 – 30	White	Patient-facing: Mixed exposure	14	0	1	0	1	1	1	0	0	0	0	n/a	Negative	Not done	Negative	Yes
010	Female	18 – 30	Asian	Patient-facing: Mixed exposure	0	0	0	0	0	1	1	0	0	0	0	n/a	Negative	Not done	Negative	No
011	Male	18 – 30	Mixed	Patient-facing: COVID wards throughout	7	0	0	0	1	0	0	0	0	1	1	Mild	Positive	Not done	Positive	No
012	Female	31-40	Asian	Non-patient facing	10	0	0	0	1	1	1	0	0	0	0	n/a	Negative	Not done	Negative	Yes
013	Female	31-40	Asian	Patient-facing: Mixed exposure	0	2	0	0	1	0	0	0	0	1	1	Mild	Positive	Not done	Positive	No
014	Female	41-50	Black	Patient facing: Unknown	0	0	0	0	0	1	1	0	0	0	0	n/a	Indeterminate	Not done	Indeterminate	No
015	Female	18 – 30	White	Patient-facing: COVID wards throughout	7	7	1	0	1	0	0	0	0	1	1	Moderate	Positive	Not done	Positive	No
016	Female	41-50	Asian	Patient-facing: COVID wards throughout	2	7	0	0	1	0	0	1	0	0	0	Moderate	Negative	Negative	Negative	No
017	Female	18 – 30	Asian	Patient-facing: Non-COVID wards only	7	3	0	0	0	1	0	0	0	1	1	Mild	Positive	Negative	Positive	Yes
018	Female	31-40	Black	Patient-facing: COVID wards throughout	0	0	0	0	1	0	1	0	0	0	0	n/a	Negative	Not done	Negative	No
019	Female	31-40	Asian	Patient-facing: COVID wards throughout	0	7	0	0	1	0	1	0	0	0	0	n/a	Negative	Not done	Negative	No
020	Female	18 – 30	White	Patient-facing: COVID wards throughout	0	0	0	0	1	0	1	0	0	0	0	n/a	Negative	Not done	Negative	No
021	Female	18 – 30	Asian	Patient-facing: Mixed exposure	8	Not answered	0	0	0	1	0	0	0	0	1	Mild	Positive	Not done	Positive	Yes
022	Female	18 – 30	Asian	Patient-facing: Mixed exposure	0	0	0	0	0	1	1	0	0	0	0	n/a	Negative	Not done	Negative	No
023	Male	51-60	White	Non-patient facing: Non-clinical hospital staff	0	0	1	0	1	0	1	0	0	0	0	n/a	Negative	Not done	Negative	No
024	Male	41-50	White	Patient-facing: Mixed exposure	0	9	0	0	1	0	0	0	1	1	1	Moderate	Positive	Positive	Positive	Yes
025	Female	51-60	Black	Non-patient facing: Non-clinical hospital staff	0	0	0	0	1	0	0	1	1	0	0	Moderate	Negative	Not done	Negative	Yes
026	Male	18 – 30	White	Patient-facing: Mixed exposure	0	0	1	1	1	1	1	0	0	0	0	n/a	Negative	Not done	Negative	No
027	Female	31-40	Asian	Patient-facing: Mixed exposure	10	0	0	0	0	1	0	0	0	1	1	Moderate	Positive	Positive	Positive	No
028	Female	18 – 30	White	Patient-facing: Non-COVID wards only	0	0	0	1	0	0	1	0	0	0	0	n/a	Negative	Positive	Positive	No
029	Female	18 – 30	Other	Patient-facing: Mixed exposure	14	0	0	0	0	1	1	0	0	0	0	n/a	Negative	Not done	Negative	Yes
030	Female	41-50	White	Patient-facing: Non-COVID wards only	0	0	0	0	1	0	1	0	0	0	0	n/a	Negative	Not done	Negative	No
031	Female	>60	Not answered	Non-patient facing: Non-clinical hospital staff	6	3	0	0	1	0	1	0	0	0	0	n/a	Negative	Not done	Negative	Yes
032	Male	18 – 30	White	Patient-facing: Mixed exposure	0	0	1	0	1	1	1	0	0	0	0	n/a	Positive	Not done	Positive	No
033	Male	31-40	White	Patient-facing: Mixed exposure	0	0	0	1	0	0	1	0	0	0	0	n/a	Negative	Not done	Negative	No
034	Female	51-60	White	Patient-facing: Mixed exposure	0	0	0	0	1	0	0	0	0	1	1	Mild	Positive	Negative	Positive	No
035	Male	31-40	White	Patient-facing: Mixed exposure	10	0	0	0	1	0	1	0	0	0	0	n/a	Negative	Not done	Negative	Yes
036	Female	51-60	White	Patient-facing: Mixed exposure	2	0	0	0	1	0	0	0	0	1	1	Mild	Negative	Negative	Negative	Yes
038	Female	18 – 30	Asian	Patient-facing: Mixed exposure	0	0	0	0	1	0	1	0	0	0	0	n/a	Negative	Not done	Negative	Yes
039	Female	18 – 30	Asian	Patient-facing: COVID wards throughout	1	1	1	0	0	0	0	1	0	0	0	Mild	Positive	Negative	Positive	No
040	Female	31-40	White	Patient-facing: Mixed exposure	7	35	1	0	1	0	0	1	1	0	0	Moderate	Negative	Not done	Negative	Yes
041	Male	51-60	White	Patient-facing: Non-COVID wards only	0	8	0	0	1	0	0	1	0	0	0	Moderate	Positive	Positive	Positive	No
042	Male	31-40	Asian	Patient facing: Unknown	7	14	0	0	1	0	0	1	1	1	1	Moderate	Positive	Negative	Positive	No
043	Female	18 – 30	Asian	Patient-facing: COVID wards throughout	0	0	1	0	1	0	1	0	0	0	0	n/a	Positive	Not done	Positive	No
044	Female	18 – 30	Black	Patient-facing: Mixed exposure	0	0	0	0	1	0	1	0	0	0	0	n/a	Positive	Not done	Positive	No
045	Female	31-40	White	Patient-facing: Mixed exposure	0	7	0	0	1	0	0	1	1	1	1	Mild	Negative	Not done	Negative	Yes
046	Female	31-40	Asian	Patient-facing: Non-COVID wards only	30	30	0	0	0	1	0	1	1	1	1	Moderate	Negative	Negative	Negative	Yes
047	Male	31-40	Asian	Patient-facing: Non-COVID wards only	14	1	1	0	0	1	0	1	1	1	1	Severe	Positive	Positive	Positive	Yes
048	Female	41-50	White	Patient-facing: Non-COVID wards only	0	0	0	0	0	1	0	1	1	0	0	Mild	Negative	Negative	Negative	No
050	Female	41-50	Black	Patient-facing: Mixed exposure	0	10	0	0	0	1	2	0	0	0	0	Mild	Negative	Not done	Negative	No
051	Female	41-50	Asian	Patient-facing: Mixed exposure	14	0	0	0	1	1	1	0	0	0	0	n/a	Negative	Not done	Negative	Yes
052	Female	41-50	White	Patient-facing: Mixed exposure	14	42	0	0	1	0	1	0	0	0	0	n/a	Negative	Not done	Negative	Yes
053	Female	18 – 30	Mixed	Patient-facing: Non-COVID wards only	0	15	0	0	1	0	0	0	0	1	1	Mild	Positive	Not done	Positive	Yes
054	Male	51-60	Not answered	Patient-facing: Non-COVID wards only	0	0	1	0	0	0	Not an	Not ans	Not an	Not answ	Not answ	n/a	Positive	Not done	Positive	Not answered
056	Female	>60	White	Patient-facing: Non-COVID wards only	0	0	1	0	1	1	1	0	0	0	0	n/a	Negative	Negative	Negative	No
057	Female	51-60	White	Patient-facing: COVID wards throughout	0	0	0	1	0	0	0	0	0	1	1	Mild	Positive	Negative	Positive	No
058	Female	51-60	White	Patient-facing: Mixed exposure	7	7	0	0	1	0	0	1	0	1	1	Moderate	Positive	Not done	Positive	No
059	Prefer not to say	51-60	Black	Patient-facing: Non-COVID wards only	14	14	0	0	1	0	0	1	0	0	0	Severe	Negative	Negative	Negative	No
060	Female	51-60	Black	Patient-facing: Mixed exposure	0	10	0	0	1	0	1	0	0	0	0	n/a	Positive	Not done	Positive	No
061	Female	18 – 30	White	Patient-facing: Mixed exposure	Not answered	Not answered	0	0	0	1	1	0	0	0	0	n/a	Negative	Not done	Negative	No
062	Female	18 – 30	Asian	Patient-facing: COVID wards throughout	0	7	0	0	1	1	0	1	0	1	1	Moderate	Positive	Negative	Positive	Yes
063	Female	41-50	Black	Patient-facing: Non-COVID wards only	Not answered	Not answered	0	0	1	0	1	0	0	0	0	n/a	Positive	Negative	Positive	No
064	Male	31-40	Black	Non-patient facing: anon	0	0	0	0	1	1	0	0	1	0	0	Mild	Negative	Not done	Negative	No
065	Female	31-40	Black	Patient-facing: Mixed exposure	0	0	0	0	1	0	1	0	0	0	0	n/a	Negative	Not done	Negative	No
066	Female	18 – 30	White	Patient-facing: Mixed exposure	0	7	0	0	1	0	0	1	1	0	0	Moderate	Negative	Not done	Negative	No
067	Female	18 – 30	Asian	Patient-facing: Mixed exposure	21	14	1	0	1	1	0	1	1	1	1	Moderate	Negative	Not done	Negative	Yes
068	Female	51-60	White	Patient facing: Unknown	Not answered	Not answered	Not answered	Not answered	Not answered	Not answered	1	0	0	0	0	n/a	Negative	Not done	Negative	No
069	Female	51-60	White	Patient-facing: Non-COVID wards only	2	0	0	0	0	1	1	0	0	0	0	n/a	Negative	Negative	Negative	Yes
070	Male	31-40	Mixed	Patient-facing: Mixed exposure	14	14	1	0	0	1	0	0	1	1	1	Moderate	Positive	Not done	Positive	No
071	Female	51-60	White	Non-patient facing: anon	0	5	1	0	0	1	0	1	0	0	1	Severe	Positive	Negative	Positive	No
072	Female	>60	White	Non-patient facing: anon	0	0	0	0	0	1	1	0	0	0	0	n/a	Negative	Not done	Negative	No
073	Female	41-50	Asian	Patient-facing: COVID wards throughout	20	0	0	0	0	1	0	0	1	1	1	Moderate	Positive	Not done	Positive	No
074	Female	18 – 30	Asian	Patient-facing: COVID wards throughout	0	0	0	0	0	1	1	0	0	0	0	n/a	Positive	Not done	Positive	No
075	Female	41-50	Not answered	Patient-facing: Mixed exposure	0	0	0	0	1	0	1	0	0	0	0	n/a	Negative	Not done	Negative	No
076	Female	41-50	Asian	Patient-facing: COVID wards throughout	0	0	0	0	0	1	1	0	0	0	0	n/a	Negative	Not done	Negative	Not answered
077	Male	>60	Other	Patient-facing: Non-COVID wards only	0	0	1	0	1	0	1	0	0	0	0	n/a	Negative	Not done	Negative	No
078	Female	18 – 30	White	Patient-facing: Mixed exposure	0	7	0	0	0	1	0	0	1	1	1	Moderate	Positive	Not done	Positive	Yes
079	Male	41-50	White	Patient-facing: Mixed exposure	0	0	0	0	0	1	1	0	0	0	0	n/a	Negative	Negative	Negative	No
080	Not answered	Not answered	Not answered	Patient facing: Unknown	Not answered	Not answered	Not answered	Not answered	Not answered	Not answered	1	0	0	0	0	n/a	Negative	Not done	Negative	No
081	Female	>60	White	Non-patient facing: anon	0	0	0	0	1	0	1	0	0	0	0	n/a	Negative	Not done	Negative	No
082	Female	18 – 30	White	Non-patient facing: anon	0	0	0	0	1	1	1	0	0	0	0	n/a	Negative	Not done	Negative	No
083	Female	41-50	Other	Patient-facing: COVID wards throughout	0	14	0	0	1	0	0	0	1	1	1	Moderate	Positive	Not done	Positive	No
084	Male	>60	White	Non-patient facing: anon	0	0	0	0	1	0	1	0	0	0	0	n/a	Negative	Not done	Negative	No
085	Male	31-40	Asian	Patient-facing: Mixed exposure	14	7	1	0	0	1	0	0	1	0	0	Moderate	Negative	Negative	Negative	Yes
086	Female	18 – 30	Mixed	Patient-facing: Mixed exposure	14	14	0	0	1	0	0	1	1	1	0	Moderate	Positive	Not done	Positive	No
087	Female	31-40	Black	Non-patient facing: anon	14	7	0	0	1	0	Not an	Not ans	Not an	Not answ	Not answ	n/a	Positive	Positive	Positive	Not answered
088	Female	51-60	White	Patient-facing: Non-COVID wards only	7	14	1	1	1	0	0	0	0	0	0	Moderate	Positive	Positive	Positive	Yes
089	Female	31-40	White	Patient-facing: Mixed exposure	0	0	0	0	0	1	1	0	0	0	0	n/a	Positive	Not done	Positive	No
090	Female	41-50	Asian	Patient-facing: Mixed exposure	0	27	1	0	0	0	0	0	1	1	0	Severe	Positive	Not done	Positive	Yes
091	Male	41-50	Asian	Patient-facing: Mixed exposure	2	0	1	0	0	0	1	0	0	0	0	n/a	Negative	Negative	Negative	Yes
092	Female	41-50	Not answered	Patient-facing: Non-COVID wards only	14	7	1	0	0	0	0	1	1	0	0	Moderate	Positive	Negative	Positive	No
093	Male	41-50	White	Non-patient facing: anon	7	3	1	0	0	0	0	1	0	0	0	Moderate	Negative	Not done	Negative	Yes
094	Female	51-60	White	Patient-facing: Mixed exposure	0	10	0	1	1	0	0	0	0	1	1	Moderate	Positive	Positive	Positive	Yes
095	Male	51-60	White	Non-patient facing: Non-clinical hospital staff	0	0	0	0	1	0	1	0	0	0	0	n/a	Negative	Negative	Negative	No
096	Female	31-40	White	Non-patient facing: Non-clinical hospital staff	0	0	0	0	1	0	1	0	0	0	0	n/a	Negative	Negative	Negative	No
097	Female	18 – 30	Asian	Patient-facing: Mixed exposure	10	0	0	0	0	1	1	0	0	0	0	n/a	Negative	Not done	Negative	Yes

098	Female	18 – 30	White	Patient-facing: Non-COVID wards only	0	0	0	0	1	1	1	0	0	0	0	n/a	Negative	Not done	Negative	No
099	Female	51-60	White	Patient-facing: Mixed exposure	0	0	0	0	1	0	1	0	0	0	0	n/a	Negative	Not done	Negative	No
100	Male	18 – 30	Asian	Non-patient facing: anon	7	5	0	0	0	1	0	1	1	0	0	Mild	Positive	Not done	Positive	Yes
101	Female	51-60	White	Non-patient facing: Non-clinical hospital staff	0	0	1	1	1	1	1	0	0	0	0	n/a	Negative	Negative	Negative	No
102	Female	>60	White	Patient-facing: Non-COVID wards only	0	1	0	0	1	0	1	0	0	0	0	n/a	Negative	Negative	Negative	No
104	Female	31-40	White	Non-patient facing: Non-clinical hospital staff	14	0	0	0	0	1	1	0	0	0	0	n/a	Negative	Negative	Negative	No
105	Female	>60	White	Non-patient facing: Non-clinical hospital staff	0	3	1	0	1	0	0	1	0	1	1	Mild	Positive	Not done	Positive	Yes
106	Male	41-50	Asian	Patient-facing: Mixed exposure	3.5	0	0	0	1	0	1	0	0	0	0	n/a	Negative	Negative	Negative	No
107	Female	18 – 30	White	Patient-facing: COVID wards throughout	2	2	0	0	1	0	0	0	1	0	0	Mild	Negative	Negative	Negative	No
108	Male	41-50	Asian	Patient-facing: Mixed exposure	0	0	0	0	1	0	1	0	0	0	0	n/a	Negative	Not done	Negative	No
109	Female	>60	Mixed	Non-patient facing: Non-clinical hospital staff	0	5	0	0	0	1	1	0	0	0	0	n/a	Negative	Negative	Negative	No
110	Female	>60	White	Patient-facing: COVID wards throughout	0	0	0	0	1	0	1	0	0	0	0	n/a	Negative	Not done	Negative	No
111	Female	41-50	White	Patient-facing: Non-COVID wards only	5	0	0	0	1	0	0	1	1	0	0	Mild	Negative	Not done	Negative	No
113	Female	18 – 30	White	Patient-facing: Mixed exposure	0	2	0	0	1	1	1	0	0	0	0	n/a	Negative	Not done	Negative	Not answered
114	Female	>60	Black	Patient-facing: Non-COVID wards only	14	0	0	0	0	1	2	Not ans	Not an	Not answe	Not answe	Mild	Positive	Not done	Positive	Yes
115	Female	41-50	Black	Patient-facing: COVID wards throughout	0	0	0	0	0	1	1	0	0	0	0	n/a	Positive	Not done	Positive	No
116	Female	41-50	White	Patient-facing: Non-COVID wards only	14	44	0	0	1	0	0	1	1	1	1	Severe	Positive	Not done	Positive	Yes
117	Female	18 – 30	White	Patient-facing: Non-COVID wards only	0	1	0	0	1	0	1	0	0	0	0	n/a	Negative	Not done	Negative	No
118	Male	18 – 30	Asian	Patient-facing: COVID wards throughout	0	14	0	0	0	1	0	1	1	1	1	Severe	Positive	Negative	Positive	Yes
119	Female	51-60	Prefer not to say	Non-patient facing: Non-clinical hospital staff	0	0	0	0	1	0	1	0	0	0	0	n/a	Negative	Negative	Negative	No
120	Female	18 – 30	Other	Patient-facing: Mixed exposure	0	0	1	0	1	1	1	0	0	0	0	n/a	Positive	Not done	Positive	No
121	Female	31-40	Black	Non-patient facing: Non-clinical hospital staff	0	3	0	0	0	1	1	0	0	0	0	n/a	Positive	Not done	Positive	No
122	Female	31-40	Asian	Patient-facing: Mixed exposure	0	14	0	0	1	0	0	0	1	1	1	Moderate	Positive	Positive	Positive	Yes
123	Female	18 – 30	Asian	Patient-facing: Mixed exposure	1	1	0	0	1	0	0	1	1	0	0	Mild	Negative	Negative	Negative	No
124	Female	41-50	White	Patient-facing: Non-COVID wards only	0	4	0	0	1	0	1	0	0	0	0	n/a	Negative	Not done	Negative	Yes
125	Female	51-60	White	Patient-facing: Non-COVID wards only	0	2	0	0	1	0	1	0	0	0	0	n/a	Negative	Negative	Negative	No
126	Female	31-40	Asian	Patient-facing: Non-COVID wards only	21	0	0	0	1	0	0	0	1	1	1	Moderate	Positive	Positive	Positive	No
127	Female	41-50	White	Patient-facing: Non-COVID wards only	7	0	0	0	1	0	Not an	Not ans	Not an	Not answe	Not answe	n/a	Negative	Negative	Negative	No
128	Female	51-60	White	Patient-facing: Non-COVID wards only	0	0	0	0	1	0	1	0	0	0	0	n/a	Negative	Not done	Negative	No
129	Female	41-50	Asian	Patient-facing: Non-COVID wards only	0	8	1	0	1	1	1	0	0	0	0	n/a	Negative	Negative	Negative	No
130	Female	51-60	White	Patient-facing: Mixed exposure	0	6	0	0	1	0	1	0	0	0	0	n/a	Negative	Negative	Negative	No
131	Female	18 – 30	Asian	Patient-facing: COVID wards throughout	0	4	0	0	1	1	0	0	1	1	1	Moderate	Indeterminate	Not done	Indeterminate	Yes
132	Female	18 – 30	White	Patient-facing: Mixed exposure	0	0	0	0	1	0	1	0	0	0	0	n/a	Negative	Not done	Negative	No
133	Female	41-50	Mixed	Patient-facing: Non-COVID wards only	14	0	0	0	1	0	0	0	0	1	1	Moderate	Negative	Not done	Negative	Yes
134	Female	18 – 30	White	Patient-facing: Non-COVID wards only	14	0	1	0	0	1	1	0	0	0	0	n/a	Negative	Negative	Negative	Yes
135	Female	31-40	White	Patient-facing: Mixed exposure	0	14	0	0	0	1	0	1	1	0	0	Moderate	Negative	Negative	Negative	Yes
136	Female	41-50	White	Patient-facing: COVID wards throughout	0	0	1	0	0	1	1	0	0	0	0	n/a	Positive	Not done	Positive	Yes
137	Female	18 – 30	White	Patient-facing: Non-COVID wards only	14	0	0	0	1	0	1	0	0	0	0	n/a	Negative	Negative	Negative	Yes
138	Female	31-40	Asian	Patient-facing: Mixed exposure	14	0	1	0	0	1	Not an	Not ans	Not an	Not answe	Not answe	n/a	Negative	Not done	Negative	Yes
139	Female	31-40	Asian	Patient-facing: Mixed exposure	0	14	0	0	1	1	0	0	0	1	0	Mild	Positive	Not done	Positive	Yes
140	Female	41-50	White	Non-patient facing: Non-clinical hospital staff	0	0	0	0	1	0	1	0	0	0	0	n/a	Negative	Not done	Negative	No
141	Female	18 – 30	White	Non-patient facing: Non-clinical hospital staff	0	0	0	0	1	0	1	0	0	0	0	n/a	Negative	Not done	Negative	No
142	Female	51-60	White	Patient-facing: Non-COVID wards only	0	0	0	0	1	0	1	0	0	0	0	n/a	Negative	Not done	Negative	No
143	Female	18 – 30	White	Patient-facing: Mixed exposure	0	0	0	0	0	1	1	0	0	0	0	n/a	Negative	Negative	Negative	No
144	Female	31-40	White	Patient-facing: Non-COVID wards only	0	7	0	0	0	1	0	1	0	0	0	Mild	Positive	Not done	Positive	Not answered
145	Female	41-50	Asian	Patient-facing: Non-COVID wards only	14	0	0	0	1	0	0	1	1	1	1	Severe	Negative	Negative	Negative	Yes
146	Female	31-40	Other	Patient-facing: Non-COVID wards only	0	0	0	0	0	1	1	0	0	0	0	n/a	Negative	Not done	Negative	No
147	Female	41-50	Other	Patient-facing: Non-COVID wards only	0	0	0	0	1	0	1	0	0	0	0	n/a	Negative	Negative	Negative	No
148	Female	41-50	White	Patient-facing: Non-COVID wards only	0	0	0	0	1	0	1	0	0	0	0	n/a	Negative	Negative	Negative	No
149	Female	18 – 30	Asian	Patient-facing: Mixed exposure	7	0	0	0	1	0	0	0	1	0	0	Moderate	Positive	Not done	Positive	Yes
150	Female	41-50	White	Patient-facing: Mixed exposure	0	14	0	0	0	1	0	1	1	0	0	Moderate	Positive	Not done	Positive	No
151	Female	51-60	Black	Patient-facing: Non-COVID wards only	0	0	0	0	0	1	1	0	0	0	0	n/a	Negative	Not done	Negative	No
152	Male	51-60	White	Patient-facing: Non-COVID wards only	0	0	0	0	1	0	1	0	0	0	0	n/a	Negative	Not done	Negative	No
153	Female	41-50	White	Patient-facing: Mixed exposure	0	0	0	0	1	0	1	0	0	0	0	n/a	Negative	Not done	Negative	No
155	Female	>60	White	Patient-facing: Non-COVID wards only	14	0	0	0	1	1	0	1	1	0	0	Mild	Positive	Not done	Positive	Yes
156	Female	41-50	Asian	Patient-facing: Non-COVID wards only	14	0	0	0	1	1	0	0	1	1	0	Moderate	Positive	Positive	Positive	No
157	Male	41-50	Not answered	Patient-facing: COVID wards throughout	7	7	1	0	0	0	0	0	1	0	0	Mild	Negative	Not done	Negative	No
158	Male	31-40	Not answered	Patient facing: Unknown	0	0	0	0	0	1	0	0	1	0	0	Mild	Negative	Negative	Negative	No
159	Female	18 – 30	Asian	Patient-facing: COVID wards throughout	14	0	1	0	0	1	0	1	0	0	0	Moderate	Negative	Not done	Negative	Yes
160	Female	>60	White	Non-patient facing: anon	0	0	0	0	1	0	1	0	0	0	0	n/a	Negative	Not done	Negative	No
161	Female	>60	White	Non-patient facing: anon	0	0	0	0	1	0	1	0	0	0	0	n/a	Negative	Not done	Negative	No
162	Female	51-60	White	Non-patient facing: Non-clinical hospital staff	3	0	0	0	1	0	0	0	0	0	0	Mild	Negative	Not done	Negative	No
163	Male	31-40	Asian	Patient-facing: Mixed exposure	20	8	0	0	0	1	0	1	1	1	1	Severe	Positive	Not done	Positive	Yes
164	Female	>60	White	Patient facing: Unknown	0	0	0	0	0	1	1	0	0	0	0	n/a	Negative	Negative	Negative	No
165	Female	41-50	White	Patient-facing: Non-COVID wards only	0	1	0	0	1	0	0	1	1	0	0	Moderate	Negative	Not done	Negative	No
166	Female	51-60	Other	Patient-facing: Mixed exposure	7	14	0	0	1	1	0	0	0	1	1	Severe	Positive	Not done	Positive	Yes
167	Female	31-40	Black	Patient-facing: Non-COVID wards only	0	0	0	0	0	1	1	0	0	0	0	n/a	Negative	Not done	Negative	No
168	Female	>60	White	Non-patient facing: anon	0	0	Not answered	Not answered	Not answered	Not answered	1	0	0	0	0	n/a	Negative	Not done	Negative	No
169	Female	18 – 30	Asian	Patient-facing: Non-COVID wards only	14	0	0	0	0	1	0	1	1	1	1	Moderate	Positive	Positive	Positive	Yes
170	Female	>60	Black	Patient-facing: Mixed exposure	7	1	0	0	1	1	1	0	0	0	0	n/a	Negative	Not done	Negative	No
171	Female	31-40	White	Patient-facing: COVID wards throughout	0	0	0	0	1	0	0	0	1	0	0	Moderate	Negative	Not done	Negative	Yes
172	Female	18 – 30	Asian	Patient-facing: Mixed exposure	7	7	0	0	0	1	0	0	0	1	0	Mild	Positive	Not done	Positive	Yes
173	Female	41-50	Asian	Patient-facing: Mixed exposure	14	41	0	0	1	0	0	1	1	1	1	Severe	Positive	Positive	Positive	Yes
174	Female	31-40	Asian	Patient-facing: COVID wards throughout	0	0	0	0	0	1	1	0	0	0	0	n/a	Negative	Not done	Negative	No
176	Female	31-40	White	Patient facing: Unknown	14	0	0	0	1	0	1	0	0	0	0	n/a	Negative	Negative	Negative	Yes
177	Male	>60	White	Patient facing: Unknown	0	0	Not answered	Not answered	Not answered	Not answered	1	0	0	0	0	n/a	Negative	Not done	Negative	No
178	Female	51-60	White	Patient-facing: Mixed exposure	10	0	1	0	1	0	0	1	0	0	0	Moderate	Negative	Not done	Negative	No
179	Female	41-50	White	Patient-facing: COVID wards throughout	0	0	0	0	1	0	1	0	0	0	0	n/a	Negative	Not done	Negative	No
180	Female	41-50	White	Patient-facing: Mixed exposure	0	0	0	0	1	1	0	0	0	1	1	Moderate	Positive	Not done	Positive	Yes
181	Female	>60	Asian	Non-patient facing: anon	14	0	0	0	1	0	0	1	0	0	0	Moderate	Negative	Not done	Negative	No
182	Male	31-40	Asian	Non-patient facing: anon	0	0	0	0	1	0	1	0	0	0	0	n/a	Negative	Not done	Negative	No
183	Female	31-40	White	Patient-facing: Non-COVID wards only	7	0	0	0	1	0	0	0	0	1	1	Mild	Positive	Positive	Positive	Yes
184	Male	41-50	White	Patient-facing: Non-COVID wards only	0	0	0	1	1	0	0	1	0	0	0	Mild	Negative	Not done	Negative	Yes
185	Female	31-40	White	Patient-facing: Non-COVID wards only	0	8	0	1	0	1	0	0	1	0	0	Mild	Positive	Not done	Positive	No
186	Female	31-40	White	Patient-facing: Non-COVID wards only	0	0	0	0	1	1	1	0	0	0	0	n/a	Negative	Not done	Negative	No
187	Female	41-50	Asian	Patient-facing: Mixed exposure	0	0	0	0	1	0	1	0	0	0	0	n/a	Negative	Not done	Negative	No
188	Female	41-50	White	Non-patient facing: Non-clinical hospital staff	14	7	1	0	0	1	0	1	0	1	0	Mild	Positive	Not done	Positive	Yes
190	Male	31-40	Other	Patient-facing: Mixed exposure	0	7	1	0	0	0	0	0	1	0	0	Mild	Positive	Not done	Positive	No
191	Female	51-60	White	Patient-facing: Mixed exposure	0	0	0	0	1	0	1	0	0	0	0	n/a	Negative	Not done	Negative	No
192	Female	41-50	White	Patient-facing: Mixed exposure	30	0	0	0	1	0	2	0	0	0	0	Moderate	Positive	Positive	Positive	Yes
193	Female	18 – 30	Asian	Patient-facing: Mixed exposure	0	21	0	0	0	1	0	1	0	0	0	Moderate	Positive	Positive	Positive	Yes
194	Female	41-50	White	Patient facing: Unknown	0	1	0	0	0	1	1	0	0	0	0	n/a	Positive	Not done	Positive	No
195	Female	31-40	White	Patient-facing: Non-COVID wards only	4	0	0	0	1	0	1	0	0	0	0	n/a	Negative	Not done	Negative	Yes
196	Female	41-50	Asian	Patient-facing: Mixed exposure	14	8	0	0	0	1	0	0	1	1	0	Severe	Positive	Negative	Positive	No
197	Female	18 – 30	Asian	Non-patient facing: anon	0	0	0	0	1	0	1	0	0	0	0	n/a	Negative	Not done	Negative	No

198	Female	31-40	Not answered	Non-patient facing: anon	0	8	0	0	0	1	Not an	Not ans	Not an	Not answe	Not answe	n/a	Negative	Not done	Negative	Not answered
199	Female	41-50	Asian	Patient-facing: Non-COVID wards only	14	1	0	0	1	1	Not an	Not ans	Not an	Not answe	Not answe	n/a	Negative	negative	Negative	Not answered
200	Female	18 – 30	Black	Patient facing: Unknown	0	0	1	0	0	0	0	0	0	1	1	Moderate	Positive	Not done	Positive	No
201	Female	51-60	White	Patient-facing: Mixed exposure	0	2	0	0	1	0	0	0	0	1	1	Mild	Positive	Negative	Positive	No
202	Female	18 – 30	White	Patient-facing: Non-COVID wards only	0	0	0	0	0	1	1	0	0	0	0	n/a	Negative	Negative	Negative	No
203	Female	31-40	White	Patient-facing: Mixed exposure	0	0	0	1	1	0	1	0	0	0	0	n/a	Negative	Not done	Negative	No
204	Female	31-40	Asian	Patient-facing: Non-COVID wards only	11	0	0	0	1	1	0	1	1	0	0	Mild	Negative	Negative	Negative	Yes
205	Female	18 – 30	Asian	Patient-facing: Mixed exposure	28	0	1	0	0	0	0	1	1	0	0	Mild	Negative	Negative	Negative	Yes
206	Male	41-50	Asian	Patient-facing: Non-COVID wards only	21	21	0	0	1	0	0	1	1	1	1	Moderate	Positive	Positive	Positive	Yes
207	Female	51-60	Asian	Patient-facing: Non-COVID wards only	0	0	0	0	1	1	1	0	0	0	0	n/a	Negative	Not done	Negative	No
208	Female	41-50	Asian	Patient-facing: Non-COVID wards only	0	14	0	0	1	0	0	0	1	0	0	Moderate	Negative	Positive	Positive	Yes
209	Male	>60	White	Patient-facing: Mixed exposure	0	2	0	1	0	0	1	0	0	0	0	n/a	Negative	Negative	Negative	No
210	Male	18 – 30	Asian	Patient-facing: Mixed exposure	7	0	1	0	0	0	0	0	1	0	0	Mild	Positive	Positive	Positive	No
211	Male	18 – 30	Asian	Patient-facing: Mixed exposure	14	7	0	0	0	1	0	1	0	0	0	Moderate	Positive	Negative	Positive	No
212	Female	41-50	White	Patient-facing: Mixed exposure	0	0	0	0	0	1	Not an	Not ans	Not an	Not answe	Not answe	n/a	Positive	Not done	Positive	No
213	Female	31-40	Asian	Patient-facing: COVID wards throughout	14	14	0	0	0	1	0	0	0	1	1	Moderate	Positive	Not done	Positive	No
214	Male	18 – 30	White	Patient-facing: Mixed exposure	10	0	0	0	0	1	0	0	1	0	0	Mild	Positive	Positive	Positive	Yes
215	Female	18 – 30	Asian	Patient-facing: COVID wards throughout	7	3	1	0	0	0	0	1	1	1	1	Moderate	Positive	Positive	Positive	Yes
216	Male	41-50	Other	Patient-facing: Mixed exposure	0	28	0	0	1	0	0	1	1	0	0	Mild	Negative	Negative	Negative	No
217	Male	31-40	Black	Patient-facing: Mixed exposure	7	0	0	0	1	0	0	1	1	0	0	Moderate	Positive	Not done	Positive	No
218	Female	31-40	White	Patient-facing: Mixed exposure	14	10	0	0	1	1	1	0	0	0	0	n/a	Negative	Negative	Negative	Yes
219	Female	51-60	White	Patient-facing: Non-COVID wards only	0	1	1	0	1	0	1	0	0	0	0	n/a	Negative	Not done	Negative	No
220	Female	31-40	White	Patient-facing: Non-COVID wards only	14	0	0	0	0	1	0	0	0	0	1	Mild	Positive	Not done	Positive	Yes
221	Female	18 – 30	Asian	Patient-facing: Non-COVID wards only	0	11	1	0	0	1	1	0	0	0	0	n/a	Positive	Not done	Positive	Yes
222	Female	41-50	White	Patient-facing: COVID wards throughout	0	0	0	0	1	0	1	0	0	0	0	n/a	Negative	Not done	Negative	No
223	Female	41-50	Asian	Patient-facing: COVID wards throughout	0	0	0	0	1	0	1	0	0	0	0	n/a	Negative	Negative	Negative	No
224	Male	31-40	White	Patient-facing: COVID wards throughout	2	2	0	0	1	0	0	0	0	0	0	Mild	Negative	Negative	Negative	No
225	Male	31-40	White	Patient-facing: COVID wards throughout	0	0	0	0	0	1	1	0	0	0	0	n/a	Negative	Not done	Negative	No
227	Female	18 – 30	Mixed	Patient-facing: COVID wards throughout	14	0	0	0	0	1	Not an	Not ans	Not an	Not answe	Not answe	n/a	Positive	Negative	Positive	Yes
228	Male	31-40	Other	Patient-facing: COVID wards throughout	0	0	0	0	1	0	1	0	0	0	0	n/a	Negative	Not done	Negative	No
229	Male	41-50	White	Patient-facing: COVID wards throughout	2	0	0	1	0	0	1	0	0	0	0	n/a	Negative	Negative	Negative	No
230	Male	18 – 30	White	Patient-facing: Mixed exposure	0	0	0	1	0	1	1	0	0	0	0	n/a	Negative	Not done	Negative	No
231	Female	41-50	White	Patient-facing: COVID wards throughout	5	0	0	0	1	0	1	0	0	0	0	n/a	Positive	Not done	Positive	No
232	Male	31-40	Asian	Patient-facing: Non-COVID wards only	14	0	0	0	0	1	1	0	0	0	0	n/a	Negative	Negative	Negative	Yes
233	Male	18 – 30	Other	Patient-facing: Non-COVID wards only	14	14	1	0	0	0	0	0	1	1	1	Mild	Positive	Positive	Positive	No
234	Female	18 – 30	White	Patient-facing: COVID wards throughout	0	7	0	0	1	0	0	1	0	0	1	Mild	Positive	Positive	Positive	No
235	Female	18 – 30	Asian	Patient-facing: Non-COVID wards only	14	0	1	0	0	0	2	0	0	0	0	Mild	Positive	Negative	Positive	Yes
236	Female	18 – 30	Asian	Patient-facing: Mixed exposure	0	0	1	0	0	0	1	0	0	0	0	n/a	Negative	Negative	Negative	Yes
237	Female	31-40	White	Patient-facing: Mixed exposure	0	14	0	0	1	0	1	0	0	0	0	n/a	Negative	Not done	Negative	No
238	Female	18 – 30	Asian	Patient-facing: COVID wards throughout	0	0	0	0	1	0	1	0	0	0	0	n/a	Positive	Not done	Positive	No
240	Female	18 – 30	Asian	Patient-facing: COVID wards throughout	9	0	0	0	1	1	0	1	0	0	0	Moderate	Negative	Negative	Negative	Yes
241	Female	>60	White	Patient-facing: COVID wards throughout	0	4	0	0	1	0	1	0	0	0	0	n/a	Negative	Not done	Negative	No
242	Female	18 – 30	Asian	Patient-facing: COVID wards throughout	0	2	0	0	0	1	1	0	0	0	0	n/a	Negative	Not done	Negative	No
243	Male	18 – 30	White	Patient-facing: COVID wards throughout	0	0	0	0	0	1	0	1	0	0	0	Mild	Negative	Not done	Negative	No
245	Female	41-50	Asian	Patient-facing: Mixed exposure	14	14	0	0	1	0	0	1	1	1	0	Moderate	Positive	Not done	Positive	Yes
246	Female	41-50	Asian	Patient-facing: Mixed exposure	0	0	0	0	1	1	Not an	Not ans	Not an	Not answe	Not answe	n/a	Positive	Not done	Positive	Not answered
247	Female	31-40	Asian	Patient-facing: Mixed exposure	9	16	0	0	0	1	0	1	1	1	1	Moderate	Positive	Positive	Positive	Yes
248	Female	18 – 30	Black	Patient-facing: Mixed exposure	14	14	0	0	0	1	0	0	0	1	1	Severe	Positive	Negative	Positive	No
249	Female	41-50	Black	Patient-facing: Mixed exposure	7	7	Not answered	Not answered	Not answered	Not answered	0	0	0	1	0	Mild	Positive	Not done	Positive	Yes
250	Female	41-50	White	Patient-facing: Mixed exposure	0	0	0	0	1	0	0	1	0	0	0	Mild	Negative	Not done	Negative	No
251	Male	51-60	Asian	Patient-facing: Non-COVID wards only	0	0	0	0	1	0	1	0	0	0	0	n/a	Negative	Negative	Negative	No
252	Male	41-50	Black	Patient-facing: COVID wards throughout	0	0	0	0	1	0	1	0	0	0	0	n/a	Negative	Negative	Negative	No
253	Female	31-40	White	Patient-facing: Non-COVID wards only	0	10	1	0	0	1	0	1	0	0	0	Severe	Negative	Not done	Negative	Yes
254	Female	31-40	Asian	Patient-facing: Non-COVID wards only	0	0	0	0	0	1	Not an	Not ans	Not an	Not answe	Not answe	n/a	Negative	Not done	Negative	No
255	Female	18 – 30	Asian	Patient-facing: Non-COVID wards only	0	0	1	0	1	1	1	0	0	0	0	n/a	Negative	Not done	Negative	No
256	Female	41-50	Asian	Patient-facing: COVID wards throughout	14	0	0	0	0	1	0	0	1	0	1	Mild	Positive	Negative	Positive	Yes
257	Male	51-60	White	Patient-facing: COVID wards throughout	0	5	0	0	1	0	0	1	0	0	0	Severe	Positive	Not done	Positive	No
258	Female	51-60	White	Patient-facing: Non-COVID wards only	0	0	0	0	1	0	1	0	0	0	0	n/a	Negative	Not done	Negative	No
259	Male	41-50	Asian	Patient facing: Unknown	14	6	0	0	1	0	0	0	1	1	1	Moderate	Positive	Positive	Positive	No
260	Female	18 – 30	White	Patient-facing: Mixed exposure	0	0	0	0	0	1	1	0	0	0	0	n/a	Negative	Not done	Negative	No
261	Male	31-40	Other	Patient-facing: Mixed exposure	0	10	0	1	0	1	0	1	1	1	0	Moderate	Positive	Positive	Positive	Yes
262	Female	31-40	Asian	Patient-facing: COVID wards throughout	7	0	1	0	0	1	0	1	1	1	1	Not answered	Negative	Not done	Negative	No
263	Female	41-50	White	Patient-facing: Mixed exposure	0	0	0	1	1	0	1	0	0	0	0	n/a	Negative	Not done	Negative	No
264	Female	41-50	White	Patient-facing: Non-COVID wards only	14	14	0	0	0	1	0	1	1	1	1	Severe	Negative	Not done	Negative	Yes
265	Female	31-40	White	Patient-facing: Non-COVID wards only	14	14	0	0	1	0	0	1	0	0	0	Mild	Negative	Not done	Negative	No
266	Female	>60	Not answered	Patient facing: Unknown	0	0	0	0	1	0	Not an	Not ans	Not an	Not answe	Not answe	n/a	Positive	Negative	Positive	No
267	Male	51-60	Black	Patient-facing: Mixed exposure	7	0	0	0	1	0	0	1	0	0	0	Moderate	Positive	Positive	Positive	Yes
268	Female	31-40	White	Patient-facing: Non-COVID wards only	0	0	0	0	1	0	1	0	0	0	0	n/a	Negative	Not done	Negative	No
269	Female	31-40	White	Patient-facing: Mixed exposure	0	0	0	0	1	0	1	0	0	0	0	n/a	Positive	Not done	Positive	No
270	Female	31-40	White	Patient-facing: Mixed exposure	7	2	0	0	1	0	2	0	0	0	0	Mild	Negative	Negative	Negative	No
271	Female	41-50	Black	Patient-facing: Non-COVID wards only	0	0	0	0	1	0	1	0	0	0	0	n/a	Negative	Not done	Negative	No
272	Male	>60	Asian	Non-patient facing: anon	0	21	0	0	1	0	Not an	Not ans	Not an	Not answe	Not answe	n/a	Positive	Not done	Positive	Not answered
273	Female	31-40	Asian	Patient-facing: Mixed exposure	0	0	0	0	1	0	0	0	0	1	0	Mild	Negative	Not done	Negative	No
274	Male	31-40	Asian	Patient-facing: Mixed exposure	2	0	0	0	1	0	2	0	0	0	0	Mild	Negative	Negative	Negative	No
275	Female	31-40	Asian	Patient-facing: Mixed exposure	0	2	0	0	0	1	1	0	0	0	0	n/a	Negative	Not done	Negative	No
276	Male	41-50	Other	Patient-facing: Mixed exposure	14	0	0	0	1	0	1	0	0	0	0	n/a	Negative	Negative	Negative	Yes
277	Female	41-50	Asian	Patient-facing: Mixed exposure	0	21	0	0	0	1	0	1	1	1	1	Moderate	Positive	Not done	Positive	No
278	Female	51-60	White	Non-patient facing: anon	10	5	1	0	0	0	0	0	1	0	0	Moderate	Negative	Negative	Negative	No
279	Female	51-60	Asian	Patient-facing: Mixed exposure	0	0	0	0	1	0	1	0	0	0	0	n/a	Negative	Negative	Negative	No
280	Male	41-50	White	Patient-facing: Mixed exposure	0	0	0	0	1	0	1	0	0	0	0	n/a	Negative	Not done	Negative	No
281	Female	18 – 30	White	Non-patient facing: anon	7	0	0	0	1	0	0	0	1	0	0	Mild	Negative	Not done	Negative	No
282	Female	51-60	White	Patient-facing: Mixed exposure	0	0	0	0	1	0	1	0	0	0	0	n/a	Positive	Not done	Positive	No
283	Female	>60	White	Patient-facing: Mixed exposure	0	0	1	0	1	0	1	0	0	0	0	n/a	Positive	Not done	Positive	No
284	Female	18 – 30	White	Patient-facing: Non-COVID wards only	0	0	0	0	1	0	1	0	0	0	0	n/a	Negative	Not done	Negative	No
285	Female	18 – 30	Asian	Patient-facing: Mixed exposure	0	0	0	0	0	1	1	0	0	0	0	n/a	Positive	Not done	Positive	No
286	Female	18 – 30	Asian	Patient-facing: Non-COVID wards only	0	0	1	0	0	1	Not an	Not ans	Not an	Not answe	Not answe	n/a	Negative	Negative	Negative	No
287	Female	18 – 30	White	Patient-facing: COVID wards throughout	14	0	0	0	1	0	0	1	0	0	0	Mild	Negative	Negative	Negative	Yes
288	Female	18 – 30	Asian	Patient-facing: COVID wards throughout	14	0	0	0	0	1	0	0	0	1	0	Mild	Positive	Not done	Positive	Not answered
289	Female	51-60	White	Patient-facing: Non-COVID wards only	14	0	0	0	1	0	Not an	Not ans	Not an	Not answe	Not answe	n/a	Negative	Not done	Negative	Not answered
290	Female	51-60	White	Patient-facing: Non-COVID wards only	0	50	0	0	1	0	1	0	0	0	0	n/a	Negative	Not done	Negative	Yes
291	Female	31-40	White	Non-patient facing: Non-clinical hospital staff	7	7	0	0	1	0	0	0	1	0	0	Mild	Negative	Negative	Negative	Yes
292	Female	18 – 30	White	Patient-facing: Non-COVID wards only	0	0	0	0	1	0	1	0	0	0	0	n/a	Negative	Not done	Negative	No
293	Male	41-50	Asian	Patient-facing: Non-COVID wards only	0	0	0	0	1	1	1	0	0	0	0	n/a	Negative	Not done	Negative	No
294	Male	41-50	Asian	Patient-facing: Non-COVID wards only	0	21	0	0	1	1	0	1	1	0	0	Moderate	Positive	Positive	Positive	Yes
295	Female	18 – 30	White	Patient-facing: Non-COVID wards only	5	5	0	0	1	0	0	1	0	1	1	Moderate	Positive	Not done	Positive	No

296	Female	18 – 30	Asian	Patient-facing: Mixed exposure	14	0	1	0	0	0	0	1	1	0	0	Mild	Positive	Not done	Positive	Yes
297	Male	31-40	Asian	Patient-facing: Non-COVID wards only	0	0	1	0	0	0	Not an	Not ans	Not an	Not answ	Not answ	n/a	Positive	Not done	Positive	Not answered
298	Female	41-50	White	Non-patient facing: anon	0	14	0	0	1	0	0	1	0	0	0	Moderate	Negative	Not done	Negative	No
299	Female	18 – 30	Asian	Patient-facing: Mixed exposure	14	0	0	0	1	1	1	0	0	0	0	n/a	Positive	Not done	Positive	Yes
300	Female	18 – 30	Black	Non-patient facing: Non-clinical hospital staff	1	2	0	0	1	0	1	0	0	0	0	n/a	Positive	Negative	Positive	No
301	Male	51-60	White	Non-patient facing: Non-clinical hospital staff	0	21	0	0	1	0	0	1	0	0	0	Moderate	Negative	Negative	Negative	No
302	Female	18 – 30	White	Non-patient facing: Non-clinical hospital staff	0	0	0	0	1	1	1	0	0	0	0	n/a	Negative		Negative	No
303	Female	51-60	White	Patient-facing: Non-COVID wards only	0	0	0	0	1	0	1	0	0	0	0	n/a	Negative		Negative	No
304	Female	18 – 30	White	Non-patient facing: Non-clinical hospital staff	8	9	0	0	1	0	1	0	0	0	0	n/a	Negative	Negative	Negative	No
305	Female	41-50	Asian	Patient-facing: Mixed exposure	10	0	1	0	0	0	0	0	0	1	0	Mild	Positive	Negative	Positive	Yes
306	Female	18 – 30	White	Non-patient facing: Non-clinical hospital staff	0	0	0	0	1	0	0	1	0	0	0	Mild	Negative		Negative	No
307	Female	31-40	White	Patient-facing: Mixed exposure	0	0	0	0	1	0	1	0	0	0	0	n/a	Negative		Negative	No
308	Female	18 – 30	Mixed	Patient-facing: Non-COVID wards only	0	0	0	0	1	0	1	0	0	0	0	n/a	Negative		Negative	No
309	Male	18 – 30	Black	Patient-facing: Mixed exposure	21	7	0	0	1	0	0	1	1	0	0	MIld	Negative	Negative	Negative	Yes
310	Female	51-60	Mixed	Non-patient facing: Non-clinical hospital staff	0	23	0	0	1	0	0	1	1	1	1	Severe	Positive		Positive	No
311	Female	18 – 30	White	Patient-facing: Mixed exposure	0	0	0	0	1	0	1	0	0	0	0	n/a	Negative	Negative	Negative	Yes
312	Not answered	Not answered	Not answered	Patient facing: Unknown	Not answered	Not answered	Not answered	Not answered	Not answered	Not answered	1	0	0	0	0	n/a	Negative	Negative	Negative	Yes
313	Male	18 – 30	Asian	Patient-facing: Mixed exposure	14	14	0	0	0	1	0	1	0	1	0	Moderate	Negative	Negative	Negative	No
314	Not answered	Not answered	Not answered	Non-patient facing: anon	Not answered	Not answered	Not answered	Not answered	Not answered	Not answered	Not an	Not ans	Not an	Not answ	Not answ	n/a	Negative	Not done	Negative	Not answered
315	Female	18 – 30	White	Non-patient facing: Non-clinical hospital staff	0	1	0	0	1	0	1	0	0	0	0	n/a	Negative	Negative	Negative	No
316	Female	>60	White	Non-patient facing: Non-clinical hospital staff	0	0	0	0	1	0	1	0	0	0	0	n/a	Negative		Negative	No
317	Female	31-40	Not answered	Patient facing: Unknown	Not answered	Not answered	Not answered	Not answered	Not answered	Not answered	Not an	Not ans	Not an	Not answ	Not answ	n/a	Negative	Not done	Negative	Not answered
318	Female	31-40	Prefer not to say	Patient-facing: Non-COVID wards only	0	0	0	0	1	0	0	0	0	1	0	Mild	Negative		Negative	No
319	Female	51-60	White	Non-patient facing: Non-clinical hospital staff	0	0	0	0	1	0	Not an	Not ans	Not an	Not answ	Not answ	n/a	Positive	Not done	Positive	Not answered
320	Female	51-60	White	Non-patient facing: Non-clinical hospital staff	0	0	1	0	1	0	0	0	0	1	1	Moderate	Negative	Negative	Negative	Yes
321	Male	31-40	Not answered	Patient-facing: Mixed exposure	0	0	1	0	0	1	1	0	0	0	0	n/a	Negative		Negative	No
322	Female	41-50	White	Non-patient facing: Non-clinical hospital staff	0	0	1	0	0	0	1	0	0	0	0	n/a	Positive		Positive	No
323	Female	51-60	White	Non-patient facing: Non-clinical hospital staff	14	0	0	0	0	1	0	0	1	0	1	Moderate	Indeterminate		Indeterminate	No
324	Female	41-50	White	Non-patient facing: anon	0	0	0	0	1	0	1	0	0	0	0	n/a	Negative		Negative	No
325	Male	>60	White	Patient-facing: Mixed exposure	7	7	0	0	1	0	0	0	0	1	1	Moderate	Positive		Positive	Yes
326	Female	18 – 30	Black	Patient-facing: Mixed exposure	0	1	0	0	0	1	1	0	0	0	0	n/a	Positive		Positive	Yes
327	Female	51-60	White	Non-patient facing: Non-clinical hospital staff	0	8	0	0	1	0	0	1	1	1	1	Moderate	Positive		Positive	Yes
328	Male	41-50	Black	Non-patient facing: Non-clinical hospital staff	0	0	0	0	1	0	1	0	0	0	0	n/a	Negative	Negative	Negative	No
329	Female	41-50	Black	Patient-facing: Non-COVID wards only	0	0	0	0	0	1	0	0	1	0	0	Moderate	Positive		Positive	No
330	Female	18 – 30	Mixed	Non-patient facing: Non-clinical hospital staff	7	0	0	0	0	1	0	0	1	0	0	Mild	Negative		Negative	No
331	Female	31-40	Asian	Patient-facing: Non-COVID wards only	14	0	0	0	0	1	0	1	0	1	1	Moderate	Positive		Positive	No
332	Female	31-40	White	Non-patient facing: Non-clinical hospital staff	Not answered	Not answered	0	0	1	0	1	0	0	0	0	n/a	Negative	Negative	Negative	No
333	Female	41-50	White	Patient-facing: Non-COVID wards only	0	0	1	0	1	0	0	0	0	1	1	Mild	Positive		Positive	No
334	Female	41-50	Asian	Patient-facing: COVID wards throughout	0	14	0	0	1	0	2	0	0	0	0	Moderate	Negative	Negative	Negative	No
335	Male	18 – 30	Asian	Patient-facing: Mixed exposure	45	0	0	0	1	0	0	1	1	1	1	Severe	Positive	Positive	Positive	Yes
336	Female	51-60	White	Non-patient facing: Non-clinical hospital staff	0	0	0	0	1	0	1	0	0	0	0	n/a	Negative		Negative	No
337	Male	41-50	White	Non-patient facing: Non-clinical hospital staff	0	0	0	0	1	0	0	0	0	0	0	Not answered	Positive		Positive	No
338	Male	>60	Mixed	Patient-facing: Mixed exposure	0	0	0	0	1	0	1	0	0	0	0	n/a	Negative		Negative	No
339	Male	18 – 30	White	Non-patient facing: Non-clinical hospital staff	0	0	1	0	0	1	1	0	0	0	0	n/a	Negative		Negative	no
340	Female	31-40	White	Patient-facing: Non-COVID wards only	68	0	0	0	1	0	0	0	1	1	1	Moderate	Negative		Negative	Yes
341	Female	18 – 30	Black	Patient-facing: Mixed exposure	0	0	1	0	0	1	1	0	0	0	0	n/a	Negative		Negative	No
342	Female	51-60	White	Patient-facing: Non-COVID wards only	14	0	0	0	0	1	2	0	0	0	0	Mild	Negative		Negative	Yes
343	Female	41-50	Other	Patient-facing: Non-COVID wards only	14	84	1	0	1	0	0	1	1	1	1	Moderate	Positive		Positive	Yes
344	Female	18 – 30	White	Non-patient facing: Non-clinical hospital staff	0	0	0	0	1	0	1	0	0	0	0	n/a	Negative		Negative	No
345	Female	41-50	Asian	Non-patient facing: Non-clinical hospital staff	0	2	1	0	1	1	1	0	0	0	0	n/a	Negative		Negative	no
346	Female	18 – 30	Mixed	Non-patient facing: Non-clinical hospital staff	0	0	1	0	0	1	1	0	0	0	0	n/a	Negative		Negative	No
347	Female	41-50	Asian	Patient-facing: Mixed exposure	0	0	0	0	0	1	Not an	Not ans	Not an	Not answ	Not answ	n/a	Positive	Not done	Positive	No
348	Male	18 – 30	Asian	Non-patient facing: Non-clinical hospital staff	7	7	0	0	0	1	0	0	1	0	0	Mild	Positive		Positive	No
349	Female	41-50	Asian	Patient-facing: COVID wards throughout	0	0	0	0	0	1	1	0	0	0	0	n/a	Negative		Negative	No
350	Male	51-60	White	Non-patient facing: Non-clinical hospital staff	0	0	0	0	0	1	Not an	Not ans	Not an	Not answ	Not answ	n/a	Negative	Not done	Negative	Not answered
351	Female	18 – 30	Black	Non-patient facing: Non-clinical hospital staff	0	7	0	0	1	1	0	0	0	0	0	Not answered	Negative		Negative	no
353	Male	31-40	Black	Patient-facing: Non-COVID wards only	Not answered	Not answered	0	0	0	1	1	0	0	0	0	n/a	Negative		Negative	No
354	Male	51-60	Black	Patient-facing: Mixed exposure	Not answered	Not answered	1	0	0	1	1	0	0	0	0	n/a	Positive	Negative	Positive	No
355	Female	51-60	Asian	Patient-facing: Mixed exposure	19	0	0	0	0	1	0	1	1	1	1	Severe	Positive	Negative	Positive	Yes
356	Male	>60	White	Non-patient facing: Non-clinical hospital staff	0	0	0	0	1	0	1	0	0	0	0	n/a	Negative		Negative	No
357	Male	>60	Black	Non-patient facing: Non-clinical hospital staff	7	63	0	0	0	1	0	0	1	0	0	Mild	Positive		Positive	No
358	Female	51-60	White	Patient-facing: Non-COVID wards only	10	0	0	0	1	0	1	0	0	0	0	n/a	Negative	Negative	Negative	No
359	Male	51-60	White	Non-patient facing: Non-clinical hospital staff	0	0	0	0	1	0	1	0	0	0	0	n/a	Positive		Positive	Yes
360	Male	41-50	White	Non-patient facing: Non-clinical hospital staff	0	14	0	0	1	0	0	0	1	0	0	Not answered	Positive		Positive	No
362	Female	51-60	White	Patient-facing: Non-COVID wards only	7	21	0	0	1	0	0	0	0	1	0	Mild	Negative	Negative	Negative	No
363	Female	51-60	Black	Non-patient facing: Non-clinical hospital staff	0	0	0	0	1	0	1	0	0	0	0	n/a	Negative		Negative	No
364	Female	41-50	Asian	Patient-facing: Mixed exposure	0	0	0	0	0	1	1	0	0	0	0	n/a	Negative		Negative	No
365	Female	51-60	White	Non-patient facing: Non-clinical hospital staff	0	0	1	0	1	1	1	0	0	0	0	n/a	Negative		Negative	No
366	Male	>60	White	Patient-facing: COVID wards throughout	0	0	0	0	1	0	1	0	0	0	0	n/a	Negative		Negative	No
367	Male	41-50	Asian	Non-patient facing: Non-clinical hospital staff	0	0	0	0	0	1	0	0	1	0	0	Mild	Negative		Negative	No
368	Female	18 – 30	White	Non-patient facing: Non-clinical hospital staff	0	4	0	0	1	0	1	0	0	0	0	n/a	Negative		Negative	No
369	Female	31-40	White	Non-patient facing: Non-clinical hospital staff	0	0	0	0	0	1	1	0	0	0	0	n/a	Negative		Negative	No
370	Female	51-60	White	Non-patient facing: Non-clinical hospital staff	0	0	0	0	1	0	1	0	0	0	0	n/a	Negative		Negative	No
371	Female	31-40	White	Non-patient facing: Non-clinical hospital staff	0	0	0	0	1	0	0	1	0	0	0	Mild	Positive	Negative	Positive	No
372	Male	51-60	White	Non-patient facing: anon	0	28	0	0	1	0	0	0	1	1	1	Moderate	Positive		Positive	No
373	Male	18 – 30	Mixed	Patient-facing: Mixed exposure	14	1	0	0	1	0	1	0	0	0	0	n/a	Negative		Negative	Yes
374	Female	41-50	Asian	Patient-facing: Mixed exposure	Not answered	Not answered	0	0	1	0	Not an	Not ans	Not an	Not answ	Not answ	n/a	Positive		Positive	No
375	Female	41-50	Black	Patient-facing: Non-COVID wards only	0	30	0	0	1	0	1	0	0	0	0	n/a	Negative		Negative	No
376	Female	18 – 30	White	Patient-facing: Mixed exposure	0	19	0	0	0	1	Not an	Not ans	Not an	Not answ	Not answ	n/a	Negative		Negative	Yes
377	Female	41-50	White	Patient-facing: Mixed exposure	0	0	0	0	1	0	1	0	0	0	0	n/a	Negative		Negative	No
378	Female	41-50	White	Patient-facing: Mixed exposure	0	2	1	0	1	0	1	0	0	0	0	n/a	Negative	Negative	Negative	No
379	Female	18 – 30	Asian	Patient-facing: Mixed exposure	7	0	0	0	0	1	0	1	1	1	1	Moderate	Negative	Negative	Negative	Yes
380	Female	41-50	Asian	Patient-facing: COVID wards throughout	14	40	1	0	1	1	0	1	0	0	0	Moderate	Positive		Positive	Yes
381	Female	31-40	White	Non-patient facing: Non-clinical hospital staff	0	3	0	0	1	0	0	1	0	0	0	Moderate	Negative	Negative	Negative	No
382	Female	51-60	Other	Patient-facing: Non-COVID wards only	6	3	0	0	1	0	0	0	1	1	1	Moderate	Positive		Positive	Yes
383	Female	41-50	Mixed	Non-patient facing: anon	0	0	0	0	1	0	Not an	Not ans	Not an	Not answ	Not answ	n/a	Negative		Negative	No
384	Female	31-40	Black	Non-patient facing: Non-clinical hospital staff	0	5	0	0	1	0	1	0	0	0	0	n/a	Negative	Negative	Negative	No
385	Female	41-50	White	Non-patient facing: anon	0	0	1	0	1	0	1	0	0	0	0	n/a	Negative		Negative	No
386	Female	18 – 30	Asian	Patient-facing: COVID wards throughout	0	9	0	0	0	1	0	1	1	1	0	Moderate	Positive		Positive	No
387	Female	18 – 30	Other	Patient-facing: COVID wards throughout	1	1	1	0	1	0	1	0	0	0	0	n/a	Negative		Negative	No
388	Male	31-40	White	Non-patient facing: Non-clinical hospital staff	0	0	0	0	1	0	1	0	0	0	0	n/a	Negative		Negative	No
389	Male	31-40	Asian	Patient-facing: Non-COVID wards only	0	0	0	0	1	0	0	1	0	1	1	Mild	Positive		Positive	No
390	Female	31-40	black	Non-patient facing: anon	0	10	0	0	1	0	0	1	1	1	0	Moderate	Negative		Negative	No
391	Female	31-40	White	Non-patient facing: Non-clinical hospital staff	0	0	0	0	1	0	0	0	0	1	1	Moderate	Indeterminate	Negative	Indeterminate	No
392	Female	18 – 30	White	Non-patient facing: Non-clinical hospital staff	14	0	0	0	1	0	0	1	0	0	0	Mild	Negative		Negative	Yes

393	Female	18 – 30	Asian	Patient-facing: Mixed exposure	0	6	0	0	0	1	0	0	1	1	1	Mild	Positive	Negative	Positive	No
394	Male	41-50	Black	Patient-facing: COVID wards throughout	7	0	0	0	1	0	0	0	1	1	1	Moderate	Positive		Positive	Yes
395	Female	18 – 30	Asian	Patient-facing: Mixed exposure	9	12	0	0	0	1	1	0	0	0	0	n/a	Negative		Negative	Yes
396	Female	31-40	White	Patient-facing: Non-COVID wards only	0	0	0	0	1	0	1	0	0	0	0	n/a	Negative		Negative	No
397	Female	41-50	White	Patient-facing: Mixed exposure	0	0	0	0	1	0	1	0	0	0	0	n/a	Negative		Negative	No
398	Female	18 – 30	Asian	Patient-facing: Mixed exposure	4	7	0	0	0	1	0	1	0	0	0	Mild	Negative		Negative	No
399	Female	31-40	White	Non-patient facing: Non-clinical hospital staff	10	0	0	0	1	0	1	0	0	0	0	n/a	Negative	Negative	Negative	Yes
400	Female	41-50	White	Non-patient facing: anon	Not answered	Not answered	0	0	0	1	1	0	0	0	0	n/a	Negative		Negative	No
401	Male	18 – 30	White	Patient-facing: Mixed exposure	0	0	0	0	1	0	Not an	Not ans	Not an	Not answ	Not answ	n/a	Positive		Positive	No
402	Male	31-40	White	Patient-facing: Mixed exposure	1	2	1	0	0	1	1	0	0	0	0	n/a	Negative	Negative	Negative	No
403	Female	41-50	Asian	Non-patient facing: Non-clinical hospital staff	Not answered	Not answered	0	0	1	0	1	0	0	0	0	n/a	Negative	Negative	Negative	No
404	Female	41-50	Black	Non-patient facing: Non-clinical hospital staff	0	0	0	0	1	0	1	0	0	0	0	n/a	Negative		Negative	No
405	Female	31-40	Mixed	Patient-facing: Non-COVID wards only	0	0	0	0	1	0	1	0	0	0	0	n/a	Negative		Negative	No
406	Female	51-60	White	Patient-facing: COVID wards throughout	14	0	0	0	1	1	0	1	1	0	0	Moderate	Positive		Positive	No
407	Female	31-40	Black	Patient-facing: COVID wards throughout	0	0	0	0	1	0	1	0	0	0	0	n/a	Negative		Negative	No
408	Male	41-50	White	Non-patient facing: Non-clinical hospital staff	0	0	0	0	1	0	1	0	0	0	0	n/a	Positive		Positive	No
409	Female	41-50	Asian	Patient-facing: Mixed exposure	0	0	0	0	1	0	1	0	0	0	0	n/a	Negative		Negative	No
410	Female	18-30	White	Patient-facing: Mixed exposure	9	0	0	0	1	0	0	1	1	1	1	Mild	Positive	Negative	Positive	No
411	Female	>60	White	Non-patient facing: Non-clinical hospital staff	0	30	0	0	1	0	0	1	0	1	1	Moderate	Positive	Positive	Positive	No
412	Female	41-50	Other	Patient-facing: Non-COVID wards only	0	0	0	0	1	0	1	0	0	0	0	n/a	Negative		Negative	No
413	Male	18 – 30	White	Non-patient facing: Non-clinical hospital staff	2	2	0	0	1	0	0	1	0	0	0	Mild	Positive		Positive	Yes
414	Female	51-60	White	Non-patient facing: Non-clinical hospital staff	6	6	0	0	1	0	0	1	1	1	1	Moderate	Positive		Positive	Yes
415	Female	41-50	White	Non-patient facing: Non-clinical hospital staff	7	15	0	0	0	1	0	1	1	1	1	Moderate	Positive		Positive	No
416	Female	31-40	Asian	Patient-facing: Mixed exposure	0	0	0	0	1	0	1	0	0	0	0	n/a	Negative		Negative	No
417	Female	31-40	Asian	Patient-facing: Mixed exposure	0	0	1	0	1	1	1	0	0	0	0	n/a	Negative		Negative	No
418	Female	18 – 30	White	Patient-facing: Mixed exposure	7	7	0	1	1	0	0	0	0	0	1	Mild	Positive		Positive	No
419	Female	18 – 30	White	Non-patient facing: Non-clinical hospital staff	5	Not answered	1	0	0	0	0	0	1	1	1	Moderate	Positive	Positive	Positive	Yes
420	Female	18-30	Asian	Patient-facing: Mixed exposure	Not answered	Not answered	0	0	0	1	0	1	0	0	0	Mild	Negative	Negative	Negative	Yes
421	Female	41-50	Asian	Patient-facing: Mixed exposure	0	14	1	0	0	0	0	0	1	1	1	Moderate	Positive	Positive	Positive	No
422	Female	18-30	White	Non-patient facing: Non-clinical hospital staff	1	0	0	0	1	0	0	1	0	0	0	Mild	Negative	Negative	Negative	No
423	Male	18 – 30	Black	Non-patient facing: Non-clinical hospital staff	0	0	1	0	1	1	1	0	0	0	0	n/a	Positive		Positive	No
424	Female	51-60	White	Non-patient facing: Non-clinical hospital staff	0	40	0	0	1	0	1	0	0	0	0	n/a	Negative		Negative	No
425	Female	18-30	White	Non-patient facing: anon	Not answered	Not answered	0	0	1	0	1	0	0	0	0	n/a	Negative		Negative	No
426	Female	51-60	White	Patient-facing: Non-COVID wards only	28	0	1	1	1	0	0	1	0	1	1	Severe	Positive		Positive	Yes
427	Female	51-60	White	Non-patient facing: Non-clinical hospital staff	7	0	0	0	1	0	Not an	Not ans	Not an	Not answ	Not answ	n/a	Negative		Negative	No
428	Female	31-40	Asian	Non-patient facing: anon	Not answered	Not answered	0	0	1	0	1	0	0	0	0	n/a	Positive		Positive	No
429	Female	51-60	Black	Patient-facing: Non-COVID wards only	Not answered	Not answered	1	0	1	0	1	0	0	0	0	n/a	Negative		Negative	No
430	Male	18 – 30	White	Non-patient facing: Non-clinical hospital staff	14	5	0	0	0	1	0	1	0	1	1	Moderate	Negative	Negative	Negative	Yes
431	Male	>60	White	Non-patient facing: Non-clinical hospital staff	Not answered	Not answered	0	0	1	0	1	0	0	0	0	n/a	Negative		Negative	No
432	Male	51-60	White	Patient-facing: Mixed exposure	0	0	0	0	1	1	0	1	1	0	0	Severe	Negative		Negative	No
433	Female	>60	White	Non-patient facing: Non-clinical hospital staff	2	0	0	0	1	0	Not an	Not ans	Not an	Not answ	Not answ	n/a	Negative	Negative	Negative	Not answered
434	Female	41-50	Not answered	Patient-facing: Non-COVID wards only	14	10	0	0	0	1	0	0	0	1	1	Not answered	Positive		Positive	No
435	Female	18 – 30	Asian	Patient-facing: Mixed exposure	18	12	0	0	0	1	0	1	1	1	1	Severe	Positive	Positive	Positive	No
436	Female	18 – 30	White	Patient-facing: Mixed exposure	3	0	0	0	1	0	0	1	0	0	0	Mild	Negative		Negative	No
437	Male	18-30	White	Non-patient facing: Non-clinical hospital staff	0	0	0	0	0	1	1	0	0	0	0	n/a	Negative		Negative	Yes
438	Female	51-60	White	Patient-facing: Mixed exposure	0	0	0	0	0	1	1	0	0	0	0	n/a	Negative		Negative	No
439	Male	>60	Asian	Patient-facing: COVID wards throughout	0	0	0	0	1	0	1	0	0	0	0	n/a	Negative	Negative	Negative	No
440	Female	41-50	White	Non-patient facing: anon	14	0	1	0	1	0	0	1	0	0	0	Not answered	Negative	Negative	Negative	Yes
441	Female	18-30	White	Patient-facing: COVID wards throughout	4	2	0	0	1	0	0	1	1	0	0	Moderate	Negative	Negative	Negative	No
442	Female	41-50	White	Patient-facing: Non-COVID wards only	50	0	0	0	1	0	0	0	1	0	0	Not answered	Negative		Negative	Yes
443	Female	51-60	White	Patient-facing: Non-COVID wards only	0	0	1	0	1	0	1	0	0	0	0	n/a	Negative		Negative	No
444	Female	51-60	White	Non-patient facing: anon	0	0	0	0	1	0	1	0	0	0	0	n/a	Negative		Negative	No
445	Female	31-40	Black	Patient-facing: Mixed exposure	0	0	0	0	0	1	1	0	0	0	0	n/a	Positive		Positive	No
446	Female	>60	White	Non-patient facing: Non-clinical hospital staff	0	6	0	0	1	1	1	0	0	0	0	n/a	Negative		Negative	No
447	Female	41-50	Other	Patient-facing: Non-COVID wards only	14	0	0	0	0	1	0	0	0	1	1	Mild	Positive	Positive	Positive	Yes
448	Female	18-30	Asian	Patient-facing: Non-COVID wards only	14	37	0	0	1	0	1	0	0	0	0	n/a	Negative		Negative	Yes
449	Male	41-50	White	Patient-facing: Mixed exposure	0	0	0	0	0	1	1	0	0	0	0	n/a	Negative	Negative	Negative	No
450	Female	51-60	Mixed	Patient-facing: Mixed exposure	0	60	0	0	1	0	1	0	0	0	0	n/a	Negative		Negative	No
451	Female	18-30	White	Patient-facing: Mixed exposure	0	0	0	0	0	1	0	0	1	1	1	Moderate	Positive	Negative	Positive	Yes
452	Female	41-50	Other	Non-patient facing: Non-clinical hospital staff	Not answered	14	0	0	1	0	0	0	1	1	0	Severe	Positive		Positive	Yes
453	Female	18 – 30	Black	Non-patient facing: Non-clinical hospital staff	0	0	0	0	1	0	1	0	0	0	0	n/a	Negative		Negative	No
454	Female	41-50	Asian	Non-patient facing: Non-clinical hospital staff	7	0	0	0	1	0	1	0	0	0	0	n/a	Negative	Negative	Negative	No
455	Female	31-40	Black	Patient-facing: Mixed exposure	Not answered	Not answered	0	0	1	0	1	0	0	0	0	n/a	Positive		Positive	No
456	Female	31-40	White	Non-patient facing: Non-clinical hospital staff	0	2	0	0	1	0	1	0	0	0	0	n/a	Negative		Negative	No
457	Female	18-30	White	Patient-facing: Mixed exposure	2	0	0	0	1	0	0	0	1	0	0	Mild	Negative	Negative	Negative	No
458	Female	18 – 30	White	Non-patient facing: Non-clinical hospital staff	Not answered	Not answered	0	0	1	0	Not an	Not ans	Not an	Not answ	Not answ	n/a	Negative	Not done	Negative	Not answered
459	Female	31-40	Other	Patient-facing: Mixed exposure	Not answered	Not answered	0	0	0	1	1	0	0	0	0	n/a	Negative		Negative	No
460	Male	41-50	Asian	Patient facing: Unknown	Not answered	Not answered	0	0	1	0	0	0	0	0	0	Mild	Positive	Negative	Positive	Yes
461	Female	41-50	Asian	Patient facing: Unknown	7	0	0	0	1	0	0	1	1	0	0	Mild	Negative		Negative	No
462	Female	51-60	White	Non-patient facing: Non-clinical hospital staff	0	0	0	0	1	0	1	0	0	0	0	n/a	Negative		Negative	No
463	Male	31-40	White	Non-patient facing: Non-clinical hospital staff	0	0	0	1	0	0	Not an	Not ans	Not an	Not answ	Not answ	n/a	Negative	Not done	Negative	Not answered
464	Female	18-30	White	Non-patient facing: Non-clinical hospital staff	0	0	0	0	1	0	Not an	Not ans	Not an	Not answ	Not answ	n/a	Positive	Not done	Positive	Not answered
465	Female	>60	White	Non-patient facing: Non-clinical hospital staff	7	8	0	0	1	0	Not an	Not ans	Not an	Not answ	Not answ	n/a	Negative	Negative	Negative	Not answered
466	Female	18 – 30	White	Patient-facing: Mixed exposure	14	10	0	0	1	0	0	1	1	0	0	Moderate	Negative		Negative	No
467	Female	18 – 30	White	Non-patient facing: Non-clinical hospital staff	0	0	0	0	0	1	1	0	0	0	0	n/a	Negative		Negative	No
468	Female	51-60	White	Non-patient facing: Non-clinical hospital staff	3	3	0	0	1	0	1	0	0	0	0	n/a	Negative	Negative	Negative	No
469	Female	18 – 30	Asian	Non-patient facing: anon	0	0	0	0	1	1	1	0	0	0	0	n/a	Negative		Negative	No
470	Female	31-40	Asian	Patient-facing: Non-COVID wards only	0	0	1	0	0	1	1	0	0	0	0	n/a	Positive		Positive	No
471	Female	51-60	White	Non-patient facing: Non-clinical hospital staff	0	0	0	0	1	0	1	0	0	0	0	n/a	Negative		Negative	No
472	Female	31-40	Asian	Patient-facing: COVID wards throughout	0	4	0	0	1	0	0	1	0	0	0	Mild	Negative	Negative	Negative	No
473	Prefer Not to Say	41-50	Asian	Patient-facing: Non-COVID wards only	0	0	0	0	1	0	1	0	0	0	0	n/a	Negative		Negative	Yes
474	Female	31-40	Asian	Patient-facing: Mixed exposure	0	0	1	0	0	0	1	0	0	0	0	n/a	Positive		Positive	No
475	Female	31-40	White	Non-patient facing: Non-clinical hospital staff	0	0	0	0	1	0	1	0	0	0	0	n/a	Negative	Negative	Negative	No
476	Female	18 – 30	Asian	Patient-facing: Mixed exposure	4	0	1	0	0	1	2	0	0	0	0	Mild	Negative	Negative	Negative	Yes
477	Female	51-60	White	Patient-facing: Mixed exposure	0	0	0	0	1	0	1	0	0	0	0	n/a	Positive		Positive	No
478	Male	18 – 30	Asian	Non-patient facing: Non-clinical hospital staff	2	1	0	0	1	0	1	0	0	0	0	n/a	Negative		Negative	No
479	Female	18 – 30	White	Patient-facing: Mixed exposure	20	14	0	0	1	0	0	0	1	0	0	Moderate	Negative	Negative	Negative	No
480	Female	51-60	Mixed	Non-patient facing: Non-clinical hospital staff	0	0	0	0	1	0	1	0	0	0	0	n/a	Negative		Negative	No
481	Female	31-40	Asian	Patient-facing: COVID wards throughout	0	0	0	0	1	1	0	0	0	0	1	Mild	Positive		Positive	No
482	Female	51-60	White	Non-patient facing: Non-clinical hospital staff	0	0	0	0	1	0	Not an	Not ans	Not an	Not answ	Not answ	n/a	Negative		Negative	No
483	Male	51-60	Not answered	Patient-facing: COVID wards throughout	Not answered	Not answered	Not answered	Not answered	Not answered	Not answered	Not an	Not ans	Not an	Not answ	Not answ	n/a	Negative	Not done	Negative	Not answered
484	Female	51-60	White	Patient-facing: Non-COVID wards only	0	10	0	0	1	0	1	0	0	0	0	n/a	Negative	Negative	Negative	No
485	Female	51-60	White	Patient-facing: Mixed exposure	14	8	0	0	1	0	0	0	0	1	0	Mild	Positive		Positive	Yes
486	Male	31-40	White	Patient-facing: COVID wards throughout	2	0	0	1	0	0	1	0	0	0	0	n/a	Negative		Negative	No
487	Female	41-50	white	Non-patient facing: Non-clinical hospital staff	0	0	0	0	1	0	1	0	0	0	0	n/a	Negative		Negative	Yes

488	Female	18 – 30	White	Non-patient facing: Non-clinical hospital staff	0	0	1	0	0	1	1	0	0	0	0	n/a	Negative		Negative	No
489	Female	18 – 30	Black	Non-patient facing: Non-clinical hospital staff	14	0	0	0	1	0	1	0	0	0	0	n/a	Negative		Negative	No
490	Female	51-60	White	Non-patient facing: Non-clinical hospital staff	0	0	0	0	1	0	0	0	1	1	1	Mild	Positive		Positive	No
491	Female	41-50	White	Patient-facing: Mixed exposure	9	7	0	0	1	0	0	0	1	0	0	Moderate	Negative	Negative	Negative	Yes
492	Female	31-40	White	Non-patient facing: anon	0	0	0	0	1	0	1	0	0	0	0	n/a	Negative		Negative	No
493	Male	18 – 30	Asian	Patient-facing: Mixed exposure	14	0	0	0	0	1	0	1	1	0	0	Mild	Negative		Negative	No
494	Male	>60	White	Non-patient facing: Non-clinical hospital staff	14	0	1	0	0	0	0	1	0	0	0	Moderate	Negative		Negative	Yes
495	Male	31-40	White	Patient-facing: Mixed exposure	0	0	0	0	0	1	1	0	0	0	0	n/a	Negative		Negative	No
496	Female	>60	White	Non-patient facing: Non-clinical hospital staff	7	0	1	0	0	0	Not an	Not ans	Not an	Not answe	Not answe	n/a	Negative	Not done	Negative	Not answered
497	Female	41-50	Other	Non-patient facing: Non-clinical hospital staff	Not answered	Not answered	Not answered	Not answered	Not answered	Not answered	1	0	0	0	0	n/a	Positive		Positive	Not answered
498	Male	41-50	Asian	Non-patient facing: Non-clinical hospital staff	14	0	0	0	0	1	1	0	0	0	0	n/a	Negative		Negative	Yes
500	Female	51-60	White	Non-patient facing: Non-clinical hospital staff	0	2	0	0	1	0	1	0	0	0	0	n/a	Negative		Negative	No
501	Male	31-40	Other	Patient-facing: COVID wards throughout	7	21	0	1	1	0	0	1	1	1	1	Severe	Positive	Positive	Positive	No
502	Female	31-40	White	Non-patient facing: Non-clinical hospital staff	0	0	0	0	1	0	1	0	0	0	0	n/a	Negative		Negative	No
503	Female	18-30	Asian	Patient-facing: Non-COVID wards only	14	0	0	0	0	1	1	0	0	0	0	n/a	Negative		Negative	Yes
504	Male	51-60	White	Non-patient facing: Non-clinical hospital staff	0	0	1	0	1	1	0	1	1	0	0	Moderate	Negative		Negative	No
505	Female	51-60	White	Non-patient facing: anon	7	7	1	0	0	0	0	0	1	1	0	Moderate	Negative	Negative	Negative	Yes
506	Female	51-60	White	Non-patient facing: Non-clinical hospital staff	0	0	0	0	1	0	1	0	0	0	0	n/a	Negative		Negative	No
507	Female	18 – 30	Mixed	Patient-facing: Mixed exposure	0	0	0	0	1	0	1	0	0	0	0	n/a	Negative		Negative	No
508	Female	51-60	White	Non-patient facing: Non-clinical hospital staff	0	0	0	0	1	0	1	0	0	0	0	n/a	Negative	Negative	Negative	No
509	Female	31-40	White	Non-patient facing: Non-clinical hospital staff	0	0	1	0	1	0	Not an	Not ans	Not an	Not answe	Not answe	n/a	Negative	Not done	Negative	Not answered
510	Female	18 – 30	Asian	Patient-facing: Non-COVID wards only	0	0	1	0	1	1	1	0	0	0	0	n/a	Negative		Negative	No
511	Female	31-40	White	Patient-facing: Mixed exposure	Not answered	0	0	0	1	0	1	0	0	0	0	n/a	Negative		Negative	No
512	Female	31-40	White	Patient-facing: Mixed exposure	0	26	0	0	1	0	0	1	1	1	1	Moderate	Positive	Positive	Positive	No
513	Female	41-50	White	Non-patient facing: Non-clinical hospital staff	Not answered	Not answered	0	0	1	0	1	0	0	0	0	n/a	Negative	Not done	Negative	Not answered
514	Female	51-60	Black	Patient-facing: COVID wards throughout	0	0	0	0	0	1	1	0	0	0	0	n/a	Positive		Positive	No
515	Male	51-60	White	Non-patient facing: Non-clinical hospital staff	Not answered	Not answered	Not answered	Not answered	Not answered	Not answered	0	0	1	0	0	Mild	Negative		Negative	No
516	Female	18 – 30	Black	Non-patient facing: Non-clinical hospital staff	Not answered	Not answered	0	0	1	0	1	0	0	0	0	n/a	Negative	Negative	Negative	No
517	Female	51-60	White	Non-patient facing: Non-clinical hospital staff	5	0	1	0	0	0	Not an	Not ans	Not an	Not answe	Not answe	n/a	Positive		Positive	Yes
518	Female	31-40	White	Patient-facing: Mixed exposure	14	Not answered	0	0	1	0	0	1	0	0	0	Mild	Negative	Negative	Negative	No
519	Female	51-60	White	Patient-facing: Non-COVID wards only	0	Not answered	0	0	0	1	1	0	0	0	0	n/a	Negative		Negative	No
520	Female	31-40	White	Patient facing: Unknown	0	3	0	0	1	0	1	0	0	0	0	n/a	Negative		Negative	No
521	Female	18 – 30	Asian	Non-patient facing: anon	7	3	0	0	1	0	0	0	1	0	0	Mild	Positive	Negative	Positive	Yes
522	Female	18 – 30	White	Patient-facing: COVID wards throughout	5	0	1	0	0	0	0	0	0	1	1	Mild	Positive	Negative	Positive	No
523	Female	51-60	Black	Patient-facing: Mixed exposure	7	7	0	0	1	0	1	0	0	0	0	n/a	Positive	Negative	Positive	Yes
524	Female	18 – 30	White	Patient-facing: Mixed exposure	4	0	0	0	1	0	0	0	1	0	0	Mild	Negative		Negative	No
525	Male	41-50	Black	Patient-facing: Non-COVID wards only	7	Not answered	0	0	1	0	2	0	0	0	0	Mild	Negative	Negative	Negative	No
526	Female	41-50	White	Non-patient facing: Non-clinical hospital staff	0	0	0	0	1	0	1	0	0	0	0	n/a	Negative		Negative	No
527	Female	18 – 30	Asian	Patient facing: Unknown	Not answered	5	0	0	1	0	0	1	0	1	1	Mild	Positive		Positive	Yes
528	Male	51-60	White	Non-patient facing: Non-clinical hospital staff	0	0	0	0	1	0	1	0	0	0	0	n/a	Positive		Positive	Yes
529	Female	>60	White	Non-patient facing: Non-clinical hospital staff	0	10	0	0	1	0	1	0	0	0	0	n/a	Negative		Negative	No
530	Female	31-40	White	Non-patient facing: Non-clinical hospital staff	0	0	0	0	1	0	1	0	0	0	0	n/a	Negative		Negative	No
531	Female	31-40	White	Non-patient facing: anon	0	1	0	0	1	0	1	0	0	0	0	n/a	Negative		Negative	No
532	Female	51-60	White	Non-patient facing: Non-clinical hospital staff	Not answered	1	1	0	0	0	1	0	0	0	0	n/a	Negative		Negative	No
533	Female	18 – 30	White	Patient-facing: Non-COVID wards only	0	2	0	0	1	0	0	0	1	0	0	Mild	Negative	Negative	Negative	No
534	Female	41-50	Black	Non-patient facing: anon	0	7	0	0	1	0	1	0	0	0	0	n/a	Negative		Negative	No
535	Female	41-50	White	Non-patient facing: Non-clinical hospital staff	9	0	0	0	1	0	0	1	1	1	0	Moderate	Negative	Negative	Negative	No
536	Female	51-60	White	Non-patient facing: Non-clinical hospital staff	0	0	0	0	1	0	Not an	Not ans	Not an	Not answe	Not answe	n/a	Negative	Negative	Negative	No
537	Female	41-50	White	Patient-facing: COVID wards throughout	0	0	0	0	1	0	0	1	0	0	0	Mild	Negative		Negative	No
538	Female	31-40	Asian	Patient-facing: Non-COVID wards only	14	12	0	0	0	1	0	1	1	1	0	Moderate	Positive		Positive	No
539	Female	31-40	Black	Patient-facing: Mixed exposure	Not answered	Not answered	0	0	1	0	1	0	0	0	0	n/a	Negative		Negative	Yes
540	Female	>60	White	Non-patient facing: Non-clinical hospital staff	0	3	0	0	1	0	0	1	0	0	0	Mild	Negative		Negative	Yes
541	Male	18 – 30	Asian	Non-patient facing: Non-clinical hospital staff	Not answered	Not answered	0	0	1	1	1	0	0	0	0	n/a	Negative		Negative	No
542	Female	41-50	Black	Non-patient facing: anon	0	0	0	0	1	0	1	0	0	0	0	n/a	Negative		Negative	No
543	Female	31-40	White	Patient-facing: Non-COVID wards only	0	0	0	0	1	0	0	0	0	1	0	Mild	Positive		Positive	Yes
544	Male	31-40	Asian	Patient-facing: Mixed exposure	14	Not answered	1	0	0	0	1	0	0	0	0	n/a	Negative		Negative	Yes
545	Male	31-40	Black	Non-patient facing: Non-clinical hospital staff	Not answered	Not answered	0	0	1	0	1	0	0	0	0	n/a	Negative		Negative	No
546	Female	51-60	Asian	Patient-facing: Mixed exposure	0	7	0	0	0	1	1	0	0	0	0	n/a	Positive		Positive	Yes
547	Female	18-30	Black	Patient facing: Unknown	0	0	0	0	0	1	1	0	0	0	0	n/a	Negative		Negative	No
548	Male	31-40	Asian	Patient-facing: Mixed exposure	7	7	0	0	0	1	0	0	1	1	0	Not answered	Positive		Positive	No
549	Female	31-40	Asian	Patient-facing: Non-COVID wards only	14	Not answered	0	0	0	1	1	0	0	0	0	n/a	Negative	Negative	Negative	Yes
550	Male	31-40	Asian	Patient-facing: Mixed exposure	Not answered	7	0	0	0	1	0	0	1	0	1	Moderate	Positive	Negative	Positive	Yes
551	Female	31-40	White	Non-patient facing: Non-clinical hospital staff	0	0	0	0	1	0	0	1	0	0	0	Mild	Negative		Negative	No
552	Female	51-60	White	Non-patient facing: Non-clinical hospital staff	0	0	0	0	1	0	1	0	0	0	0	n/a	Negative		Negative	No
553	Female	51-60	White	Non-patient facing: Non-clinical hospital staff	14	0	0	0	1	0	0	1	0	1	0	Moderate	Positive		Positive	Yes
554	Female	31-40	White	Patient-facing: Non-COVID wards only	0	0	0	0	0	1	0	0	1	1	0	Severe	Negative		Negative	Yes
555	Female	41-50	Black	Patient-facing: COVID wards throughout	0	0	0	0	1	0	1	0	0	0	0	n/a	Negative		Negative	No
556	Female	41-50	Asian	Patient-facing: Mixed exposure	14	0	0	0	1	0	1	0	0	0	0	n/a	Positive		Positive	Yes
557	Female	41-50	Asian	Patient-facing: COVID wards throughout	0	0	0	0	1	1	1	0	0	0	0	n/a	Positive		Positive	No
558	Female	31-40	Asian	Patient-facing: COVID wards throughout	Not answered	Not answered	1	0	0	0	1	0	0	0	0	n/a	Positive		Positive	Yes
559	Male	31-40	White	Patient-facing: Mixed exposure	0	2	0	0	1	0	1	0	0	0	0	n/a	Negative		Negative	No
560	Female	51-60	White	Patient-facing: Non-COVID wards only	Not answered	9	1	0	1	0	0	0	1	0	0	Moderate	Positive		Positive	No
561	Female	51-60	White	Non-patient facing: anon	0	0	0	0	1	0	1	0	0	0	0	n/a	Negative		Negative	No
562	Female	31-40	Black	Patient-facing: COVID wards throughout	2	0	0	0	1	0	0	0	0	0	1	Mild	Positive		Positive	Yes
563	Female	31-40	Black	Non-patient facing: anon	0	3	0	0	1	0	1	0	0	0	0	n/a	Negative		Negative	No
564	Female	Not answered	Other	Patient-facing: Non-COVID wards only	7	7	0	0	0	1	0	0	1	1	0	Moderate	Negative		Negative	No
565	Female	51-60	White	Non-patient facing: anon	7	0	0	0	0	1	0	1	0	0	0	Not answered	Negative		Negative	No
566	Female	51-60	White	Patient-facing: Mixed exposure	10	0	0	0	1	0	2	0	0	0	0	Mild	Positive		Positive	Yes
567	Male	41-50	Asian	Patient-facing: Non-COVID wards only	0	21	0	0	1	0	0	1	1	1	1	Moderate	Positive		Positive	Yes
568	Male	51-60	White	Non-patient facing: anon	0	0	0	0	1	0	1	0	0	0	0	n/a	Negative		Negative	No
569	Male	51-60	Asian	Non-patient facing: anon	0	0	0	0	1	1	1	0	0	0	0	n/a	Negative		Negative	No
570	Female	31-40	White	Patient-facing: Mixed exposure	7	0	0	0	1	0	2	0	0	0	0	Mild	Positive		Positive	Yes
571	Female	18 – 30	Asian	Patient-facing: COVID wards throughout	0	0	1	1	0	1	1	0	0	0	0	n/a	Negative		Negative	Yes
572	Male	18 – 30	Asian	Patient-facing: Non-COVID wards only	Not answered	Not answered	1	0	0	1	0	0	1	0	0	Moderate	Negative	Negative	Negative	Yes
573	Male	41-50	Other	Non-patient facing: anon	0	0	0	0	1	0	1	0	0	0	0	n/a	Negative		Negative	No
574	Female	51-60	White	Non-patient facing: anon	0	1	0	0	0	1	1	0	0	0	0	n/a	Negative		Negative	Yes
575	Male	51-60	White	Non-patient facing: anon	10	Not answered	1	0	0	0	1	0	0	0	0	n/a	Negative	Negative	Negative	Yes
576	Female	51-60	White	Non-patient facing: anon	0	0	1	0	1	1	1	0	0	0	0	n/a	Negative		Negative	No
577	Female	51-60	White	Non-patient facing: anon	0	0	0	0	0	1	1	0	0	0	0	n/a	Negative		Negative	No
578	Female	51-60	White	Patient-facing: Mixed exposure	0	10	0	0	1	1	0	0	1	1	0	Moderate	Positive		Positive	No
579	Female	31-40	Mixed	Non-patient facing: Non-clinical hospital staff	7	Not answered	0	0	1	0	0	1	0	0	0	Mild	Negative	Negative	Negative	Yes
580	Female	41-50	Black	Patient-facing: Mixed exposure	Not answered	Not answered	0	0	0	1	1	0	0	0	0	n/a	Indeterminate		Indeterminate	No
581	Female	18 – 30	White	Patient-facing: Mixed exposure	9	Not answered	0	0	1	0	1	0	0	0	0	n/a	Negative	Negative	Negative	Yes
582	Male	51-60	White	Non-patient facing: anon	0	0	0	1	0	0	0	0	0	1	0	Mild	Positive		Positive	Yes
583	Female	18 – 30	Asian	Patient-facing: Non-COVID wards only	21	0	0	0	1	0	1	0	0	0	0	n/a	Negative		Negative	No

584	Female	51-60	Asian	Patient-facing: Mixed exposure	5	0	0	0	1	0	0	1	0	0	0	Mild	Negative	Negative	Negative	No
585	Male	31-40	White	Patient-facing: Non-COVID wards only	0	0	1	0	0	1	1	0	0	0	0	n/a	Negative		Negative	No
586	Female	41-50	Asian	Patient-facing: Mixed exposure	7	14	0	0	1	0	0	1	1	1	1	Moderate	Positive		Positive	Yes
587	Female	41-50	White	Patient-facing: COVID wards throughout	0	5 (other)	0	0	1	0	1	0	0	0	0	n/a	Negative	negative	Negative	No
588	Female	31-40	Asian	Patient-facing: COVID wards throughout	14	Not answered	1	0	0	0	1	0	0	0	0	n/a	Negative		Negative	Yes
589	Female	18 – 30	Asian	Patient-facing: COVID wards throughout	0	4	1	0	0	1	0	0	1	0	0	Not answered	Positive		Positive	No
590	Female	>60	White	Patient-facing: Non-COVID wards only	0	2	0	0	1	0	0	0	1	0	0	Moderate	Negative	Negative	Negative	No
591	Female	41-50	White	Patient-facing: Non-COVID wards only	Not answered	7	0	0	1	0	0	0	1	1	1	Moderate	Positive		Positive	Yes
592	Female	41-50	Other	Patient-facing: COVID wards throughout	7	46	0	0	1	0	0	0	1	1	1	Moderate	Positive		Positive	Yes
593	Female	31-40	Asian	Patient-facing: COVID wards throughout	Not answered	Not answered	0	0	0	1	1	0	0	0	0	n/a	Positive		Positive	Yes
594	Female	31-40	Not answered	Patient-facing: COVID wards throughout	Not answered	2	0	0	1	0	1	0	0	0	0	n/a	Positive		Positive	No
596	Female	>60	Black	Patient-facing: Mixed exposure	Not answered	5	0	0	0	1	Not an	Not ans	Not an	Not answ	Not answ	n/a	Positive		Positive	No
597	Female	18 – 30	Asian	Patient-facing: Mixed exposure	14	14	0	0	0	1	0	1	1	1	1	Moderate	Positive	Positive	Positive	No
598	Female	18 – 30	Asian	Patient-facing: Mixed exposure	14	12	0	0	0	1	0	1	1	0	0	Moderate	Negative	Negative	Negative	Yes
599	Male	31-40	Black	Patient-facing: Mixed exposure	0	0	0	0	1	1	Not an	Not ans	Not an	Not answ	Not answ	n/a	Positive	Not done	Positive	Not answered
600	Female	31-40	White	Non-patient facing: Non-clinical hospital staff	0	0	1	0	1	0	1	0	0	0	0	n/a	Negative		Negative	Yes
601	Female	51-60	White	Non-patient facing: Non-clinical hospital staff	21	14	0	0	1	0	0	1	1	1	1	Severe	Positive	Positive	Positive	Yes
602	Female	31-40	Asian	Patient-facing: Mixed exposure	7	Not answered	0	0	0	1	0	0	1	0	0	Moderate	Positive	Positive	Positive	Yes
603	Not answered	51-60	Black	Patient-facing: Non-COVID wards only	0	0	0	0	1	0	1	0	0	0	0	n/a	Negative		Negative	No
604	Female	31-40	Black	Patient-facing: Non-COVID wards only	Not answered	18	0	0	1	0	1	0	0	0	0	n/a	Positive		Positive	No
605	Female	41-50	White	Non-patient facing: Non-clinical hospital staff	0	0	0	0	1	0	1	0	0	0	0	n/a	Negative		Negative	No
606	Not answered	41-50	White	Patient-facing: Mixed exposure	0	0	0	0	1	0	1	0	0	0	0	n/a	Negative		Negative	No
607	Female	51-60	White	Patient-facing: COVID wards throughout	14	5	0	0	1	0	0	0	1	0	0	Mild	Positive		Positive	Yes
608	Female	41-50	White	Patient-facing: Mixed exposure	0	0	0	0	1	0	0	0	1	1	0	Moderate	Negative		Negative	Yes
609	Male	51-60	White	Non-patient facing: Non-clinical hospital staff	0	0	0	0	1	0	0	0	0	0	0	Not answered	Negative		Negative	Yes
610	Female	31-40	White	Patient-facing: Non-COVID wards only	0	Not answered	0	0	1	0	1	0	0	0	0	n/a	Negative		Negative	No
611	Female	51-60	White	Non-patient facing: Non-clinical hospital staff	1	Not answered	0	0	1	0	1	0	0	0	0	n/a	Positive		Positive	No
612	Male	51-60	White	Non-patient facing: Non-clinical hospital staff	0	0	1	0	1	0	1	0	0	0	0	n/a	Negative		Negative	No
613	Female	51-60	White	Non-patient facing: Non-clinical hospital staff	0	0	0	0	1	0	1	0	0	0	0	n/a	Negative		Negative	No
614	Female	41-50	White	Patient-facing: Mixed exposure	Not answered	Not answered	0	0	0	1	1	0	0	0	0	n/a	Negative		Negative	No
615	Female	51-60	White	Patient-facing: Mixed exposure	0	0	0	0	0	1	0	0	0	1	1	Mild	Positive		Positive	No
616	Female	18 – 30	Black	Patient-facing: Mixed exposure	0	0	0	0	0	1	1	0	0	0	0	n/a	Negative		Negative	No
617	Male	31-40	Asian	Patient-facing: Mixed exposure	7	7	0	0	1	0	0	0	1	0	0	Mild	Positive		Positive	Yes
618	Female	>60	White	Patient-facing: Mixed exposure	0	0	0	0	1	0	1	0	0	0	0	n/a	Positive		Positive	No
619	Female	41-50	White	Non-patient facing: Non-clinical hospital staff	0	0	1	0	0	0	1	0	0	0	0	n/a	Negative		Negative	No
620	Female	18-30	Asian	Patient-facing: Mixed exposure	14	0	0	0	0	1	1	0	0	0	0	n/a	Negative		Negative	Yes
621	Female	31-40	Asian	Patient-facing: Mixed exposure	14	Not answered	1	0	0	1	2	0	0	0	0	Mild	Positive		Positive	Yes
622	Female	51-60	White	Non-patient facing: anon	Not answered	Not answered	0	0	1	0	Not an	Not ans	Not an	Not answ	Not answ	n/a	Negative		Negative	No
623	Female	18 – 30	Black	Patient-facing: Mixed exposure	7	3	0	0	0	1	0	1	1	1	1	Moderate	Indeterminate		Indeterminate	No
624	Female	18 – 30	Asian	Patient-facing: Mixed exposure	4	0	0	0	0	1	0	1	1	1	0	Moderate	Positive		Positive	No
625	Male	31-40	Unknown	Patient-facing: Mixed exposure	7	14	0	0	0	1	0	0	0	1	1	Moderate	Positive		Positive	No
626	Female	18 – 30	White	Patient-facing: Non-COVID wards only	0	0	0	0	0	1	1	0	0	0	0	n/a	Negative		Negative	Yes
627	Female	18 – 30	Asian	Non-patient facing: Non-clinical hospital staff	0	0	0	0	1	0	1	0	0	0	0	n/a	Negative		Negative	No
628	Female	51-60	Other	Patient-facing: COVID wards throughout	16	0	1	0	0	0	0	1	1	1	0	Moderate	Positive		Positive	Yes
629	Female	31-40	Asian	Patient-facing: Non-COVID wards only	14	Not answered	0	0	0	1	0	1	0	1	1	Mild	Indeterminate		Indeterminate	Yes
631	Female	51-60	White	Patient-facing: Mixed exposure	0	0	0	0	1	0	2	0	0	0	0	Mild	Positive		Positive	No
632	Female	51-60	White	Non-patient facing: Non-clinical hospital staff	0	0	0	0	1	0	1	0	0	0	0	n/a	Negative		Negative	No
633	Female	31-40	Asian	Patient-facing: Non-COVID wards only	7	60	0	0	0	1	0	1	1	1	1	Moderate	Positive		Positive	Yes
634	Female	51-60	White	Non-patient facing: Non-clinical hospital staff	Not answered	15	1	0	1	0	1	0	0	0	0	n/a	Negative		Negative	Yes
635	Male	31-40	Black	Patient-facing: Mixed exposure	0	0	0	0	1	0	1	0	0	0	0	n/a	Positive		Positive	No
636	Female	51-60	White	Non-patient facing: Non-clinical hospital staff	0	0	0	0	1	0	1	0	0	0	0	n/a	Negative		Negative	No
637	Female	41-50	Other	Patient-facing: COVID wards throughout	Not answered	Not answered	0	0	1	1	Not an	Not ans	Not an	Not answ	Not answ	n/a	Negative	Not done	Negative	Yes
638	Female	41-50	White	Non-patient facing: Non-clinical hospital staff	0	0	0	0	1	0	1	0	0	0	0	n/a	Negative		Negative	No
639	Male	31-40	Asian	Non-patient facing: anon	Not answered	6	0	0	1	0	0	0	1	1	1	Severe	Negative		Negative	Yes
640	Female	51-60	White	Non-patient facing: Non-clinical hospital staff	5	Not answered	0	0	1	0	0	0	1	1	0	Moderate	Positive		Positive	Yes
641	Female	41-50	White	Non-patient facing: anon	0	0	1	0	1	0	1	0	0	0	0	n/a	Negative		Negative	No
642	Female	18 – 30	Asian	Patient-facing: Non-COVID wards only	Not answered	0	0	0	0	1	0	0	1	1	1	Mild	Positive		Positive	No
643	Female	31-40	White	Non-patient facing: Non-clinical hospital staff	0	0	0	0	1	1	1	0	0	0	0	n/a	Negative		Negative	No
644	Female	>60	White	Patient-facing: COVID wards throughout	0	0	1	0	1	1	1	0	0	0	0	n/a	Negative		Negative	No
645	Male	41-50	White	Non-patient facing: Non-clinical hospital staff	Not answered	11	0	0	1	0	Not an	Not ans	Not an	Not answ	Not answ	n/a	Positive	Negative	Positive	Not answered
646	Male	18 – 30	Asian	Non-patient facing: Non-clinical hospital staff	0	0	0	0	1	0	Not an	Not ans	Not an	Not answ	Not answ	n/a	Negative		Negative	No
647	Female	31-40	White	Non-patient facing: Non-clinical hospital staff	Not answered	5	0	0	1	0	0	1	0	0	0	Moderate	Negative		Negative	No
648	Female	51-60	White	Non-patient facing: Non-clinical hospital staff	5	1	0	0	1	0	1	0	0	0	0	n/a	Negative		Negative	Yes
649	Male	31-40	Asian	Non-patient facing: Non-clinical hospital staff	14	20	0	1	1	0	0	0	1	1	1	Moderate	Positive		Positive	Yes
650	Female	18 – 30	White	Patient-facing: COVID wards throughout	0	0	0	0	1	0	1	0	0	0	0	n/a	Negative		Negative	No
651	Female	51-60	Black	Patient-facing: Non-COVID wards only	6	Not answered	Not answered	Not answered	Not answered	Not answered	0	1	0	0	0	Moderate	Positive		Positive	No
652	Female	51-60	White	Patient-facing: Mixed exposure	0	0	0	0	1	0	1	0	0	0	0	n/a	Negative		Negative	No
653	Female	31-40	Asian	Patient-facing: Non-COVID wards only	Not answered	Not answered	1	0	0	0	0	0	0	1	1	Moderate	Positive		Positive	No
654	Male	18 – 30	Asian	Non-patient facing: Non-clinical hospital staff	Not answered	Not answered	0	1	0	1	0	0	0	1	1	Mild	Positive		Positive	No
655	Female	51-60	White	Non-patient facing: Non-clinical hospital staff	Not answered	7	1	0	0	1	1	0	0	0	0	n/a	Negative		Negative	No
656	Female	41-50	White	Non-patient facing: Non-clinical hospital staff	0	0	0	0	1	0	1	0	0	0	0	n/a	Negative		Negative	No
657	Male	18 – 30	White	Non-patient facing: Non-clinical hospital staff	0	0	0	0	1	0	1	0	0	0	0	n/a	Negative		Negative	No
658	Female	31-40	Prefer not to say	Patient-facing: Mixed exposure	0	0	0	0	1	0	1	0	0	0	0	n/a	Negative		Negative	No
659	Male	31-40	white	Patient-facing: Non-COVID wards only	14	0	0	0	0	1	0	1	1	1	1	Moderate	Negative		Negative	Yes
660	Female	41-50	Asian	Patient-facing: Non-COVID wards only	7	0	0	1	0	Not answered	0	0	0	1	1	Mild	Positive		Positive	Yes
661	Male	18 – 30	White	Patient-facing: Mixed exposure	Not answered	Not answered	0	0	1	0	1	0	0	0	0	n/a	Negative		Negative	No
662	Female	31-40	Asian	Patient-facing: Non-COVID wards only	2	0	1	0	0	1	0	1	0	0	0	Mild	Positive		Positive	Yes
663	Female	41-50	White	Non-patient facing: anon	0	0	0	0	1	0	1	0	0	0	0	n/a	Negative		Negative	No
664	Female	31-40	White	Non-patient facing: anon	0	0	0	0	1	0	1	0	0	0	0	n/a	Negative		Negative	No
665	Female	51-60	White	Non-patient facing: Non-clinical hospital staff	0	0	0	0	1	0	1	0	0	0	0	n/a	Negative	Negative	Negative	No
666	Male	31-40	White	Non-patient facing: Non-clinical hospital staff	14	Not answered	0	0	1	0	2	0	0	0	0	Mild	Positive		Positive	Yes
667	Male	31-40	White	Patient-facing: Non-COVID wards only	0	0	0	0	0	1	1	0	0	0	0	n/a	Negative		Negative	No
668	Female	41-50	Asian	Patient-facing: Non-COVID wards only	14	Not answered	0	0	0	1	1	0	0	0	0	n/a	Negative		Negative	No
669	Female	>60	White	Non-patient facing: anon	Not answered	2	1	0	1	0	1	0	0	0	0	n/a	Negative		Negative	No
670	Female	51-60	White	Non-patient facing: Non-clinical hospital staff	14	3	0	0	1	0	0	0	1	1	1	Moderate	Positive		Positive	No
671	Female	41-50	White	Patient-facing: Non-COVID wards only	0	0	1	0	1	0	0	0	1	0	0	Mild	Negative		Negative	No
672	Female	31-40	White	Patient-facing: Non-COVID wards only	0	2	0	0	0	1	0	1	0	0	0	Mild	Negative		Negative	No
673	Female	41-50	Black	Patient-facing: Non-COVID wards only	0	0	0	0	1	0	1	0	0	0	0	n/a	Negative	Not done	Negative	Not answered
674	Female	41-50	White	Non-patient facing: Non-clinical hospital staff	0	0	0	0	1	0	1	0	0	0	0	n/a	Negative		Negative	Yes
675	Male	18 – 30	White	Non-patient facing: Non-clinical hospital staff	0	0	0	0	1	0	1	0	0	0	0	n/a	Positive		Positive	No
676	Male	51-60	Asian	Non-patient facing: Non-clinical hospital staff	0	0	1	0	1	0	1	0	0	0	0	n/a	Negative		Negative	No
677	Female	>60	White	Patient-facing: Non-COVID wards only	0	1	0	0	1	0	1	0	0	0	0	n/a	Negative		Negative	No
678	Female	18-30	Asian	Patient-facing: Non-COVID wards only	7	Not answered	0	0	0	1	0	0	1	1	1	Moderate	Positive		Positive	Yes
679	Female	51-60	White	Non-patient facing: Non-clinical hospital staff	0	1	0	1	0	0	1	0	0	0	0	n/a	Negative		Negative	No
680	Female	18 – 30	White	Patient-facing: Non-COVID wards only	0	0	0	0	1	0	1	0	0	0	0	n/a	Negative		Negative	No

681	Female	41-50	White	Patient-facing: Mixed exposure	0	0	1	0	0	0	1	0	0	0	0	n/a	Positive		Positive	No
682	Female	18 – 30	White	Patient-facing: COVID wards throughout	0	7	0	0	0	1	1	0	0	0	0	n/a	Negative		Negative	No
683	Female	51-60	White	Patient-facing: COVID wards throughout	7	Not answered	0	0	0	1	0	0	0	1	1	Moderate	Positive		Positive	Not answered
684	Female	41-50	White	Patient-facing: Mixed exposure	Not answered	5	0	0	1	1	0	0	1	1	1	Mild	Negative		Negative	No
685	Female	41-50	Black	Patient-facing: COVID wards throughout	0	0	0	0	0	1	1	0	0	0	0	n/a	Positive		Positive	No
686	Male	31-40	White	Non-patient facing: Non-clinical hospital staff	0	0	0	0	1	0	1	0	0	0	0	n/a	Negative		Negative	No
687	Female	41-50	Black	Patient-facing: Mixed exposure	0	0	0	0	0	1	1	0	0	0	0	n/a	Negative		Negative	No
688	Female	18 – 30	Mixed	Non-patient facing: anon	0	0	0	0	0	1	Not an	Not ans	Not an	Not answ	Not answ	n/a	Negative	Not done	Negative	Not answered
689	Female	51-60	White	Non-patient facing: anon	0	Not answered	1	0	1	1	0	1	1	0	0	Moderate	Negative		Negative	Yes
690	Female	41-50	Asian	Patient-facing: COVID wards throughout	0	0	0	0	1	0	1	0	0	0	0	n/a	Negative		Negative	No
691	Male	18 – 30	Asian	Patient-facing: Mixed exposure	7	Not answered	0	0	0	1	1	0	0	0	0	n/a	Negative		Negative	No
692	Male	31-40	White	Non-patient facing: Non-clinical hospital staff	14	0	0	0	1	0	1	0	0	0	0	n/a	Positive		Positive	Yes
693	Female	31-40	White	Patient-facing: Mixed exposure	0	14	0	0	1	0	2	0	0	0	0	Moderate	Negative		Negative	Yes
694	Female	41-50	Asian	Patient-facing: Mixed exposure	Not answered	20	0	0	1	0	0	1	1	1	1	Moderate	Positive		Positive	Yes
695	Female	31-40	White	Non-patient facing: anon	0	0	0	0	1	0	1	0	0	0	0	n/a	Negative		Negative	No
696	Female	31-40	White	Non-patient facing: Non-clinical hospital staff	49	31	0	0	1	1	0	1	1	1	1	Moderate	Negative		Negative	Yes
697	Female	18 – 30	White	Non-patient facing: Non-clinical hospital staff	0	0	0	1	0	0	1	0	0	0	0	n/a	Negative		Negative	No
698	Female	41-50	White	Non-patient facing: Non-clinical hospital staff	5	2	0	0	1	0	0	0	1	0	0	Moderate	Negative		Negative	Yes
699	Female	31-40	White	Non-patient facing: Non-clinical hospital staff	Not answered	Not answered	0	0	1	0	1	0	0	0	0	n/a	Negative		Negative	No
700	Female	31-40	White	Non-patient facing: Non-clinical hospital staff	7	Not answered	0	0	1	0	0	0	1	0	0	Mild	Positive		Positive	No
701	Female	18 – 30	Asian	Patient-facing: Mixed exposure	14	0	0	0	0	1	0	0	1	0	0	Mild	Positive		Positive	Yes
702	Female	41-50	Asian	Patient-facing: Mixed exposure	0	21	0	0	1	0	0	0	1	1	1	Moderate	Positive		Positive	No
703	Female	18 – 30	Asian	Patient-facing: Mixed exposure	0	3	0	0	0	1	1	0	0	0	0	n/a	Negative		Negative	No
704	Male	51-60	White	Non-patient facing: anon	0	0	1	1	1	0	1	0	0	0	0	n/a	Negative		Negative	No
705	Male	41-50	Asian	Patient-facing: Non-COVID wards only	56	0	0	0	0	1	0	1	1	1	1	Severe	Positive		Positive	No
706	Female	41-50	White	Patient-facing: COVID wards throughout	0	1	0	0	1	1	1	0	0	0	0	n/a	Negative		Negative	No
707	Female	51-60	White	Patient-facing: Mixed exposure	0	0	0	0	1	0	1	0	0	0	0	n/a	Negative		Negative	No
708	Female	51-60	White	Patient-facing: Non-COVID wards only	60	5	0	0	1	0	2	0	0	0	0	Mild	Negative		Negative	No
709	Male	18 – 30	Mixed	Patient-facing: Mixed exposure	3	0	0	0	0	1	0	0	0	1	1	Mild	Negative		Negative	Yes
710	Female	18 – 30	White	Patient-facing: Mixed exposure	0	1	1	0	0	1	0	1	0	0	0	Mild	Positive		Positive	No
711	Male	18 – 30	Asian	Patient-facing: COVID wards throughout	14	0	0	0	0	1	0	0	1	0	0	Mild	Negative		Negative	Yes
712	Female	>60	White	Patient-facing: Non-COVID wards only	0	0	1	0	1	0	1	0	0	0	0	n/a	Negative		Negative	No
713	Female	51-60	White	Patient-facing: COVID wards throughout	0	0	0	0	1	0	1	0	0	0	0	n/a	Negative		Negative	No
714	Female	41-50	White	Patient-facing: Non-COVID wards only	0	3	0	0	0	1	1	0	0	0	0	n/a	Negative		Negative	No
715	Female	51-60	White	Patient-facing: Mixed exposure	0	0	0	0	1	0	1	0	0	0	0	n/a	Negative		Negative	No
716	Male	41-50	Asian	Patient-facing: Mixed exposure	3	0	0	0	1	0	1	0	0	0	0	n/a	Negative		Negative	No
717	Female	41-50	White	Non-patient facing: Non-clinical hospital staff	7	56	0	0	1	0	0	0	1	1	1	Moderate	Positive		Positive	Yes
718	Male	51-60	Prefer not to say	Patient-facing: Mixed exposure	9	9	0	0	1	0	0	1	1	1	1	Severe	Positive		Positive	Yes
719	Female	>60	White	Patient-facing: Non-COVID wards only	0	0	0	0	1	0	1	0	0	0	0	n/a	Negative		Negative	No
720	Female	31-40	Asian	Patient-facing: COVID wards throughout	14	21	0	0	1	0	1	0	0	0	0	n/a	Positive		Positive	No
721	Female	51-60	White	Patient-facing: COVID wards throughout	6	3	0	0	0	1	0	1	0	1	1	Moderate	Negative		Negative	No
722	Female	51-60	White	Patient-facing: COVID wards throughout	6	6	0	0	1	0	0	1	0	1	1	Moderate	Negative		Negative	No
723	Female	41-50	White	Patient-facing: COVID wards throughout	0	0	1	0	1	0	1	0	0	0	0	n/a	Negative		Negative	No
724	Male	>60	Not answered	Patient-facing: COVID wards throughout	0	0	0	0	1	0	1	0	0	0	0	n/a	Negative		Negative	No
725	Female	41-50	White	Patient-facing: Non-COVID wards only	0	0	1	0	0	0	1	0	0	0	0	n/a	Negative		Negative	No
726	Female	51-60	White	Patient-facing: Mixed exposure	0	0	0	1	0	1	1	0	0	0	0	n/a	Positive		Positive	No
727	Female	31-40	Asian	Patient-facing: COVID wards throughout	0	14	0	0	0	1	0	0	1	1	1	Moderate	Positive		Positive	Yes
729	Female	18 – 30	White	Patient-facing: Non-COVID wards only	14	0	0	0	1	0	Not an	Not ans	Not an	Not answ	Not answ	n/a	Negative	Not done	Negative	Not answered
731	Female	41-50	Asian	Patient-facing: Mixed exposure	0	0	0	0	1	1	1	0	0	0	0	n/a	Negative		Negative	No
732	Female	>60	White	Patient-facing: Non-COVID wards only	0	7	0	0	1	0	0	1	0	1	0	Moderate	Positive		Positive	No
733	Female	18 – 30	White	Patient-facing: COVID wards throughout	14	0	0	0	1	0	1	0	0	0	0	n/a	Negative		Negative	Yes
734	Male	41-50	White	Patient-facing: Non-COVID wards only	0	1	0	0	0	1	1	0	0	0	0	n/a	Negative		Negative	No
735	Female	18 – 30	White	Patient-facing: Non-COVID wards only	0	0	0	0	1	0	1	0	0	0	0	n/a	Negative		Negative	No
737	Female	18 – 30	White	Patient-facing: Non-COVID wards only	0	0	0	0	1	0	1	0	0	0	0	n/a	Negative		Negative	Yes
738	Female	41-50	Asian	Patient-facing: COVID wards throughout	0	2	0	0	1	1	0	0	1	0	0	Mild	Negative		Negative	No
739	Female	31-40	Asian	Patient-facing: Non-COVID wards only	7	0	0	0	0	1	0	1	0	1	1	Moderate	Positive		Positive	Yes
740	Female	31-40	White	Patient-facing: Non-COVID wards only	0	0	0	1	0	0	1	0	0	0	0	n/a	Negative		Negative	No
741	Female	31-40	Black	Patient-facing: Mixed exposure	12	7	0	0	0	1	Not an	Not ans	Not an	Not answ	Not answ	n/a	Positive		Positive	No
742	Male	51-60	White	Patient-facing: Mixed exposure	0	0	1	0	0	0	1	0	0	0	0	n/a	Negative		Negative	No
743	Male	18 – 30	White	Patient-facing: Mixed exposure	0	0	0	1	0	0	1	0	0	0	0	n/a	Negative		Negative	No
744	Female	>60	White	Patient-facing: Non-COVID wards only	0	0	0	0	1	0	1	0	0	0	0	n/a	Negative		Negative	Yes
745	Female	31-40	Asian	Patient-facing: Mixed exposure	7	10	0	0	0	1	0	1	1	0	0	Moderate	Positive		Positive	No
747	Male	31-40	White	Patient-facing: COVID wards throughout	0	0	0	0	1	0	1	0	0	0	0	n/a	Positive		Positive	Yes
748	Female	51-60	White	Patient-facing: Non-COVID wards only	0	0	0	0	1	0	2	0	0	0	0	Not answered	Negative		Negative	No
749	Female	41-50	White	Non-patient facing: Non-clinical hospital staff	0	2	0	0	1	0	1	0	0	0	0	n/a	Negative		Negative	No
750	Male	31-40	Asian	Patient-facing: COVID wards throughout	0	4	0	0	0	1	Not an	Not ans	Not an	Not answ	Not answ	n/a	Negative		Negative	No
751	Male	41-50	White	Patient-facing: COVID wards throughout	12	0	0	0	1	0	0	1	1	0	0	Moderate	Negative		Negative	No
752	Female	41-50	White	Patient-facing: COVID wards throughout	0	0	0	0	1	0	1	0	0	0	0	n/a	Negative		Negative	No
753	Male	41-50	White	Patient-facing: Mixed exposure	0	0	1	0	1	1	1	0	0	0	0	n/a	Negative		Negative	No
754	Female	18 – 30	White	Patient-facing: Non-COVID wards only	14	0	0	0	1	0	0	0	0	1	1	Severe	Positive		Positive	Yes
755	Male	18 – 30	White	Non-patient facing: Non-clinical hospital staff	0	0	0	0	1	0	1	0	0	0	0	n/a	Negative		Negative	No
756	Female	18 – 30	White	Patient-facing: Non-COVID wards only	0	3	0	0	1	0	1	0	0	0	0	n/a	Negative		Negative	Yes
757	Female	41-50	White	Non-patient facing: Non-clinical hospital staff	14	0	0	0	1	0	2	0	0	0	0	Mild	Positive		Positive	Yes
758	Female	51-60	White	Non-patient facing: Non-clinical hospital staff	7	1	0	0	1	0	1	0	0	0	0	n/a	Negative		Negative	Yes
759	Female	18 – 30	Black	Patient-facing: Non-COVID wards only	0	28	0	0	0	1	0	1	1	1	1	Severe	Positive		Positive	Yes
760	Female	51-60	White	Non-patient facing: Non-clinical hospital staff	14	0	0	0	1	0	0	1	0	1	0	Moderate	Positive		Positive	No
761	Male	18 – 30	Asian	Non-patient facing: Non-clinical hospital staff	14	0	0	0	1	0	1	0	0	0	0	n/a	Negative		Negative	Yes
762	Female	51-60	White	Non-patient facing: Non-clinical hospital staff	0	0	0	0	1	0	0	1	1	1	0	Severe	Negative		Negative	Yes
763	Female	51-60	White	Patient-facing: Non-COVID wards only	0	0	0	0	1	0	1	0	0	0	0	n/a	Negative		Negative	No
764	Female	>60	White	Non-patient facing: Non-clinical hospital staff	0	0	0	0	1	0	1	0	0	0	0	n/a	Negative		Negative	No
765	Female	31-40	White	Patient-facing: Non-COVID wards only	0	0	0	0	1	0	1	0	0	0	0	n/a	Negative		Negative	No
767	Female	18 – 30	Asian	Patient-facing: Non-COVID wards only	7	7	0	0	1	0	0	1	0	1	1	Moderate	Positive		Positive	No
768	Female	41-50	Asian	Patient-facing: COVID wards throughout	25	0	0	0	1	0	1	0	0	0	0	n/a	Negative		Negative	No
769	Male	31-40	Asian	Patient-facing: Mixed exposure	0	0	0	0	0	1	1	0	0	0	0	n/a	Negative		Negative	No
770	Female	18 – 30	Asian	Patient-facing: Non-COVID wards only	0	0	1	0	1	1	1	0	0	0	0	n/a	Negative		Negative	Yes
771	Female	18 – 30	White	Patient-facing: Non-COVID wards only	0	1	0	0	1	0	1	0	0	0	0	n/a	Negative		Negative	No
773	Female	18 – 30	Not answered	Patient-facing: Non-COVID wards only	0	3	0	0	1	0	1	0	0	0	0	n/a	Negative		Negative	No
774	Female	18 – 30	White	Patient-facing: Mixed exposure	0	3	0	0	1	0	1	0	0	0	0	n/a	Negative		Negative	No
775	Female	41-50	White	Patient-facing: Non-COVID wards only	4	6	0	0	0	1	0	0	0	1	1	Mild	Positive		Positive	Yes
776	Female	18 – 30	White	Patient-facing: Non-COVID wards only	0	0	0	1	0	1	1	0	0	0	0	n/a	Negative		Negative	No
777	Female	51-60	White	Patient-facing: Non-COVID wards only	0	4	0	0	0	1	1	0	0	0	0	n/a	Negative		Negative	No
778	Female	18 – 30	White	Patient-facing: Non-COVID wards only	16	7	0	0	1	0	1	0	0	0	0	n/a	Negative		Negative	Yes
779	Male	41-50	White	Patient-facing: COVID wards throughout	0	0	0	0	1	0	0	1	0	0	0	Mild	Positive		Positive	Yes
780	Female	51-60	White	Patient-facing: Mixed exposure	0	1	1	1	1	0	1	0	0	0	0	n/a	Positive		Positive	No
781	Male	31-40	Other	Patient-facing: COVID wards throughout	0	4	0	0	0	1	0	0	1	0	0	Moderate	Negative		Negative	No

782	Female	18 – 30	White	Patient-facing: Non-COVID wards only	0	0	0	0	1	0	1	0	0	0	0	n/a	Negative		Negative	No
783	Male	41-50	Asian	Patient-facing: COVID wards throughout	9	9	0	0	1	0	0	1	0	0	0	Mild	Negative		Negative	No
784	Female	18 – 30	White	Patient-facing: Mixed exposure	8	0	0	0	1	0	0	1	0	0	0	Moderate	Negative		Negative	No
785	Female	31-40	Not answered	Patient-facing: COVID wards throughout	7	0	0	0	0	1	0	0	0	0	1	Moderate	Positive		Positive	No
787	Female	31-40	Asian	Patient-facing: Mixed exposure	0	21	1	0	1	0	0	1	0	1	0	Moderate	Positive		Positive	Yes
788	Female	31-40	Asian	Patient-facing: Mixed exposure	0	16	1	0	1	1	0	1	0	1	1	Moderate	Negative		Negative	No
789	Female	18 – 30	Black	Patient-facing: Mixed exposure	0	7	0	0	1	0	0	1	1	0	0	Mild	Positive		Positive	No
790	Male	18 – 30	Asian	Patient-facing: Non-COVID wards only	14	9	0	0	1	0	0	0	0	1	1	Mild	Positive		Positive	Yes
791	Female	18 – 30	White	Patient-facing: Non-COVID wards only	0	0	0	0	0	1	0	0	0	1	1	Mild	Positive		Positive	No
792	Female	51-60	White	Non-patient facing: Non-clinical hospital staff	0	0	0	0	1	0	1	0	0	0	0	n/a	Negative		Negative	No
794	Female	51-60	White	Non-patient facing: Non-clinical hospital staff	0	0	0	0	1	0	1	0	0	0	0	n/a	Negative		Negative	No
795	Female	18 – 30	White	Patient-facing: Mixed exposure	4	0	0	0	1	0	1	0	0	0	0	n/a	Negative		Negative	No
796	Female	>60	White	Patient-facing: Non-COVID wards only	0	1	0	0	1	0	1	0	0	0	0	n/a	Negative		Negative	No
797	Female	31-40	White	Patient-facing: Non-COVID wards only	0	11	0	0	1	0	0	0	1	0	0	Mild	Negative		Negative	No
798	Female	51-60	Black	Patient-facing: Non-COVID wards only	0	0	0	0	1	0	0	1	0	0	0	Moderate	Positive		Positive	No
799	Male	18 – 30	White	Patient-facing: Mixed exposure	0	0	0	0	1	0	1	0	0	0	0	n/a	Negative		Negative	No
800	Female	41-50	White	Patient-facing: Non-COVID wards only	0	7	1	0	1	0	0	0	1	1	1	Not answered	Negative		Negative	No
801	Male	18 – 30	Asian	Patient-facing: Mixed exposure	0	21	0	0	1	0	0	1	0	1	0	Moderate	Negative		Negative	No
802	Female	18 – 30	White	Patient-facing: Non-COVID wards only	14	2	0	0	1	0	0	0	0	1	1	Moderate	Positive		Positive	Yes
803	Female	18 – 30	White	Patient-facing: Mixed exposure	0	0	0	0	0	1	1	0	0	0	0	n/a	Negative		Negative	No
804	Female	31-40	Asian	Non-patient facing: Non-clinical hospital staff	14	60	0	0	1	0	0	1	1	1	1	Severe	Positive		Positive	Yes
805	Male	41-50	White	Patient-facing: COVID wards throughout	0	15	0	0	1	0	0	0	1	1	1	Moderate	Positive		Positive	Yes
806	Female	18 – 30	White	Patient-facing: Mixed exposure	14	7	0	0	1	0	0	1	1	1	0	Moderate	Negative		Negative	Yes
807	Female	51-60	White	Non-patient facing: Non-clinical hospital staff	0	0	1	0	0	0	1	0	0	0	0	n/a	Negative		Negative	No
808	Female	31-40	Asian	Patient-facing: Non-COVID wards only	14	0	0	0	0	1	1	0	0	0	0	n/a	Negative		Negative	Yes
809	Female	51-60	Asian	Patient-facing: Non-COVID wards only	0	0	0	0	1	0	1	0	0	0	0	n/a	Negative		Negative	No
810	Female	18 – 30	White	Patient-facing: Mixed exposure	0	0	0	0	1	0	1	0	0	0	0	n/a	Negative		Negative	No
811	Female	31-40	Asian	Patient-facing: Non-COVID wards only	12	0	0	0	0	1	1	0	0	0	0	n/a	Negative		Negative	Yes
812	Female	51-60	White	Patient-facing: Mixed exposure	0	2	0	0	1	1	0	1	1	0	0	Moderate	Negative		Negative	Yes
813	Male	41-50	Black	Patient-facing: Mixed exposure	0	1	0	0	1	0	Not an	Not ans	Not an	Not answ	Not answ	n/a	Negative	Not done	Negative	Not answered
814	Female	18 – 30	White	Patient-facing: Non-COVID wards only	9	21	0	0	1	0	1	0	0	0	0	n/a	Negative		Negative	Yes
816	Female	18 – 30	White	Patient-facing: Non-COVID wards only	21	0	0	0	1	0	1	0	0	0	0	n/a	Negative		Negative	Yes
817	Female	31-40	White	Patient-facing: Non-COVID wards only	7	7	1	0	0	1	0	1	0	1	1	Moderate	Positive		Positive	No
818	Female	41-50	White	Patient-facing: Non-COVID wards only	14	18	0	0	1	0	0	0	1	0	0	Moderate	Negative		Negative	Yes
819	Female	31-40	Asian	Patient-facing: Non-COVID wards only	0	0	0	0	1	0	1	0	0	0	0	n/a	Negative		Negative	Yes
820	Female	31-40	Prefer not to say	Patient-facing: Non-COVID wards only	14	0	0	0	1	0	0	0	0	1	1	Mild	Negative		Negative	Yes
821	Male	18 – 30	White	Patient-facing: Mixed exposure	5	5	0	0	0	1	0	1	0	0	0	Moderate	Positive		Positive	No
822	Female	18 – 30	Asian	Patient-facing: Mixed exposure	0	0	1	0	0	1	1	0	0	0	0	n/a	Negative	Not done	Negative	Not answered
823	Male	31-40	White	Patient-facing: Mixed exposure	0	0	0	0	1	0	1	0	0	0	0	n/a	Negative		Negative	No
824	Female	18 – 30	White	Patient-facing: COVID wards throughout	7	15	0	0	0	1	0	1	1	0	1	Moderate	Positive		Positive	Yes
825	Female	31-40	Asian	Patient-facing: COVID wards throughout	0	0	0	0	0	1	1	0	0	0	0	n/a	Positive		Positive	No
826	Female	18 – 30	Black	Patient-facing: Mixed exposure	14	5	0	0	0	1	0	0	0	1	0	Severe	Positive		Positive	Yes
827	Female	31-40	Asian	Patient-facing: Non-COVID wards only	0	0	1	0	0	1	1	0	0	0	0	n/a	Negative		Negative	No
828	Female	31-40	Asian	Patient-facing: Mixed exposure	0	0	1	0	0	0	1	0	0	0	0	n/a	Negative		Negative	No
829	Female	41-50	White	Patient-facing: COVID wards throughout	0	0	0	0	1	0	1	0	0	0	0	n/a	Positive		Positive	No
830	Male	31-40	Prefer not to say	Patient-facing: Mixed exposure	7	7	0	0	1	0	0	0	1	1	1	Moderate	Positive		Positive	No
831	Male	31-40	Asian	Patient-facing: Mixed exposure	7	10	0	0	0	1	0	1	1	1	1	Severe	Positive		Positive	Yes
832	Female	51-60	White	Patient-facing: COVID wards throughout	0	13	0	0	1	0	0	1	0	1	1	Severe	Positive		Positive	Yes
833	Female	>60	White	Non-patient facing: Non-clinical hospital staff	0	0	0	0	1	0	1	0	0	0	0	n/a	Negative		Negative	No
834	Female	51-60	Black	Patient-facing: Non-COVID wards only	7	0	0	0	1	0	0	1	1	0	0	Severe	Positive		Positive	No
835	Female	31-40	Black	Non-patient facing: Non-clinical hospital staff	14	7	0	0	1	0	0	0	0	1	1	Moderate	Positive		Positive	Yes
836	Female	41-50	White	Non-patient facing: Non-clinical hospital staff	0	0	0	0	1	0	1	0	0	0	0	n/a	Negative		Negative	Yes
837	Male	18 – 30	Asian	Patient-facing: Mixed exposure	0	0	1	0	0	0	1	0	0	0	0	n/a	Negative		Negative	No
838	Female	18 – 30	Asian	Patient-facing: Mixed exposure	0	0	0	0	0	1	1	0	0	0	0	n/a	Negative		Negative	No
839	Male	31-40	White	Patient-facing: Mixed exposure	0	0	0	1	0	0	1	0	0	0	0	n/a	Negative		Negative	No
840	Male	>60	White	Non-patient facing: Non-clinical hospital staff	0	0	1	0	1	1	1	0	0	0	0	n/a	Negative		Negative	No
841	Female	51-60	Not answered	Patient facing: Unknown	30	0	0	0	1	0	0	0	1	1	1	Moderate	Negative		Negative	Yes
842	Female	51-60	White	Patient-facing: COVID wards throughout	0	6	0	0	1	0	0	1	0	1	0	Moderate	Positive		Positive	No
843	Female	18 – 30	Asian	Patient-facing: Non-COVID wards only	0	0	0	0	0	1	1	0	0	0	0	n/a	Negative		Negative	No
844	Female	41-50	White	Patient-facing: Mixed exposure	7	0	0	0	0	1	0	1	1	0	0	Moderate	Negative		Negative	No
845	Female	18 – 30	Asian	Patient-facing: Mixed exposure	0	0	0	0	0	1	1	0	0	0	0	n/a	Positive		Positive	No
846	Female	51-60	Other	Patient-facing: COVID wards throughout	7	7	0	0	1	0	0	0	0	1	1	Moderate	Negative		Negative	No
847	Female	51-60	White	Patient-facing: Non-COVID wards only	0	0	0	0	1	0	1	0	0	0	0	n/a	Negative		Negative	No
848	Female	51-60	White	Patient-facing: Non-COVID wards only	0	14	0	0	1	0	1	0	0	0	0	n/a	Positive		Positive	No
849	Female	18 – 30	Black	Patient-facing: Non-COVID wards only	14	21	1	0	1	0	0	1	1	0	0	Moderate	Negative		Negative	No
850	Female	51-60	White	Patient-facing: COVID wards throughout	0	0	0	0	1	0	1	0	0	0	0	n/a	Negative		Negative	No
851	Female	18 – 30	White	Patient-facing: Non-COVID wards only	0	0	0	0	1	0	1	0	0	0	0	n/a	Negative		Negative	No
852	Female	>60	White	Patient-facing: Mixed exposure	0	0	0	0	1	0	1	0	0	0	0	n/a	Negative		Negative	No
853	Female	41-50	Asian	Patient-facing: COVID wards throughout	14	0	0	0	1	0	0	0	1	1	0	Moderate	Positive		Positive	Yes
854	Female	>60	White	Patient-facing: Non-COVID wards only	0	7	0	0	1	0	0	1	1	0	0	Moderate	Negative		Negative	No
855	Female	18 – 30	Black	Patient-facing: Mixed exposure	4	0	0	0	1	0	Not an	Not ans	Not an	Not answ	Not answ	n/a	Negative		Negative	Yes
856	Female	31-40	Asian	Patient-facing: Mixed exposure	0	7	0	0	1	0	0	0	1	0	0	Mild	Negative		Negative	No
858	Female	41-50	Asian	Non-patient facing: Non-clinical hospital staff	0	0	0	0	1	0	1	0	0	0	0	n/a	Negative		Negative	No
859	Female	51-60	Asian	Patient-facing: Non-COVID wards only	0	0	0	0	1	1	0	0	0	1	1	Mild	Negative		Negative	No
860	Female	51-60	White	Patient-facing: Non-COVID wards only	7	7	1	0	0	0	0	0	1	1	1	Moderate	Positive		Positive	Yes
861	Female	31-40	Asian	Patient facing: Unknown	0	0	0	0	1	0	1	0	0	0	0	n/a	Negative		Negative	No
862	Female	18 – 30	Asian	Patient-facing: Non-COVID wards only	0	7	1	0	0	0	0	0	1	0	0	Mild	Negative		Negative	No
863	Female	51-60	Black	Patient-facing: Non-COVID wards only	0	0	0	0	1	0	Not an	Not ans	Not an	Not answ	Not answ	n/a	Negative		Negative	No
864	Female	41-50	Asian	Patient-facing: Non-COVID wards only	0	0	0	0	1	0	2	0	0	0	0	Mild	Negative		Negative	No
865	Female	31-40	White	Patient-facing: Mixed exposure	21	0	0	0	1	0	0	1	0	1	0	Moderate	Negative		Negative	No
866	Male	41-50	Asian	Patient-facing: Mixed exposure	14	3	0	0	0	1	0	1	0	0	0	Mild	Negative		Negative	Yes
867	Female	41-50	White	Patient-facing: Mixed exposure	7	0	0	0	0	1	0	1	1	0	0	Moderate	Positive		Positive	Yes
868	Female	51-60	White	Patient-facing: Mixed exposure	0	0	0	0	0	1	1	0	0	0	0	n/a	Negative		Negative	No
869	Female	31-40	Mixed	Patient-facing: Mixed exposure	0	0	0	0	1	0	0	1	0	0	0	Mild	Negative		Negative	Yes
870	Male	18 – 30	White	Patient-facing: Mixed exposure	0	0	0	0	1	0	0	1	1	0	0	Moderate	Positive		Positive	No
871	Male	18 – 30	Black	Patient-facing: Mixed exposure	0	0	0	0	0	1	1	0	0	0	0	n/a	Negative		Negative	No
872	Female	31-40	Asian	Patient-facing: COVID wards throughout	0	0	0	0	1	0	0	1	0	0	0	Mild	Negative		Negative	No
873	Female	18 – 30	White	Patient-facing: COVID wards throughout	0	0	0	0	1	0	0	1	1	1	1	Moderate	Negative		Negative	Yes
874	Male	31-40	White	Patient-facing: COVID wards throughout	0	0	1	0	1	0	1	0	0	0	0	n/a	Negative		Negative	Yes
875	Female	41-50	White	Patient-facing: Mixed exposure	0	3	1	0	0	0	0	0	1	0	0	Mild	Positive		Positive	No
876	Female	41-50	Asian	Patient-facing: Mixed exposure	0	21	1	0	1	0	1	0	0	0	0	n/a	Negative		Negative	Yes
877	Female	>60	White	Patient-facing: Mixed exposure	3	0	1	0	1	0	1	0	0	0	0	n/a	Negative		Negative	No
878	Male	51-60	Asian	Patient-facing: Non-COVID wards only	0	3	0	0	1	0	0	1	0	0	0	Mild	Negative		Negative	No
879	Female	51-60	White	Patient-facing: Non-COVID wards only	0	9	0	0	1	0	0	0	0	1	1	Severe	Positive		Positive	Yes
880	Female	41-50	White	Patient-facing: Non-COVID wards only	0	0	0	0	1	0	1	0	0	0	0	n/a	Negative		Negative	No

881	Female	51-60	Black	Patient-facing: COVID wards throughout	14	60	1	0	0	0	0	1	1	1	1	Severe	Positive		Positive	No
882	Female	41-50	Asian	Patient-facing: Mixed exposure	0	0	0	0	1	1	0	0	1	0	1	Mild	Positive		Positive	Yes
884	Male	31-40	Asian	Patient-facing: COVID wards throughout	0	0	0	0	1	0	0	0	1	1	1	Moderate	Positive		Positive	Yes
885	Female	18 – 30	Asian	Patient-facing: Mixed exposure	7	1	0	0	1	1	0	1	1	1	0	Moderate	Positive		Positive	Yes
886	Female	41-50	Other	Patient-facing: Mixed exposure	7	7	0	0	0	1	0	0	0	1	1	Severe	Positive		Positive	Yes
888	Female	>60	White	Non-patient facing: Non-clinical hospital staff	0	0	1	0	0	1	1	0	0	0	0	n/a	Negative		Negative	No
889	Male	18 – 30	White	Patient-facing: Non-COVID wards only	0	0	0	0	0	1	1	0	0	0	0	n/a	Negative		Negative	No
890	Male	18 – 30	Black	Patient-facing: Mixed exposure	0	0	0	0	1	0	Not an	Not ans	Not an	Not answ	Not answ	n/a	Negative	Not done	Negative	Not answered
891	Female	18 – 30	White	Non-patient facing: Non-clinical hospital staff	0	0	0	0	1	0	1	0	0	0	0	n/a	Negative	Not done	Negative	Not answered
892	Female	18 – 30	White	Patient-facing: Mixed exposure	14	0	1	0	1	1	0	1	1	1	1	Moderate	Positive		Positive	Yes
893	Male	41-50	Asian	Patient-facing: Mixed exposure	0	14	0	0	1	0	0	1	1	1	1	Severe	Positive		Positive	No
894	Female	18 – 30	White	Patient-facing: Non-COVID wards only	14	2	0	0	1	0	Not an	Not ans	Not an	Not answ	Not answ	n/a	Negative	Not done	Negative	Not answered
895	Female	51-60	White	Patient-facing: Non-COVID wards only	3	0	0	0	1	0	0	0	0	1	1	Mild	Negative		Negative	Yes
896	Male	18 – 30	Asian	Patient-facing: Mixed exposure	14	4	1	0	0	1	0	0	1	1	1	Moderate	Positive		Positive	No
897	Female	18 – 30	Asian	Patient-facing: Non-COVID wards only	7	0	1	0	0	0	1	0	0	0	0	n/a	Negative		Negative	Yes
898	Female	31-40	Black	Patient-facing: Mixed exposure	0	0	0	0	0	1	Not an	Not ans	Not an	Not answ	Not answ	n/a	Positive		Positive	No
899	Female	18 – 30	White	Patient-facing: Non-COVID wards only	0	0	0	0	1	0	1	0	0	0	0	n/a	Negative		Negative	No
900	Female	18 – 30	White	Patient-facing: Non-COVID wards only	0	0	0	0	1	0	1	0	0	0	0	n/a	Negative		Negative	No
901	Female	41-50	White	Patient-facing: COVID wards throughout	14	0	0	0	1	0	0	0	1	0	0	Mild	Positive		Positive	Yes
902	Female	18 – 30	Asian	Patient-facing: COVID wards throughout	14	0	1	0	0	1	1	0	0	0	0	n/a	Negative		Negative	Yes
903	Male	51-60	Asian	Patient-facing: Mixed exposure	14	16	1	0	0	0	0	0	1	1	1	Moderate	Positive		Positive	No
904	Female	18 – 30	Asian	Patient-facing: Mixed exposure	0	14	0	0	0	1	1	0	0	0	0	n/a	Positive		Positive	Yes
905	Female	31-40	White	Patient-facing: Non-COVID wards only	14	12	0	0	1	0	0	0	1	1	1	Mild	Positive		Positive	Yes
906	Female	51-60	White	Patient-facing: COVID wards throughout	0	0	0	0	1	0	1	0	0	0	0	n/a	Negative		Negative	No
907	Male	51-60	White	Patient-facing: Mixed exposure	0	2	0	0	1	0	2	0	0	0	0	Mild	Negative		Negative	Yes
908	Female	18 – 30	Asian	Patient-facing: COVID wards throughout	14	14	0	0	0	1	0	1	1	0	0	Mild	Negative		Negative	No
909	Female	51-60	White	Patient-facing: COVID wards throughout	0	120	1	0	1	0	0	1	1	1	1	Moderate	Positive		Positive	Yes
910	Male	31-40	Asian	Patient-facing: Non-COVID wards only	0	0	0	0	0	1	1	0	0	0	0	n/a	Negative	Not done	Negative	Yes
911	Female	41-50	Asian	Patient-facing: Non-COVID wards only	0	0	0	0	1	1	1	0	0	0	0	n/a	Negative		Negative	No
912	Male	31-40	Black	Non-patient facing: Non-clinical hospital staff	7	0	0	0	1	0	1	0	0	0	0	n/a	Negative		Negative	Yes
913	Female	31-40	Asian	Patient-facing: Mixed exposure	Not answered	0	0	0	1	1	1	0	0	0	0	n/a	Negative		Negative	Yes
914	Female	31-40	Asian	Patient-facing: Mixed exposure	14	18	0	0	1	0	0	1	1	0	1	Moderate	Positive		Positive	No
915	Male	51-60	White	Patient-facing: Non-COVID wards only	0	0	0	0	1	0	1	0	0	0	0	n/a	Positive		Positive	No
916	Female	51-60	White	Patient-facing: Non-COVID wards only	7	35	0	0	0	1	0	0	1	1	0	Severe	Positive		Positive	No
917	Male	51-60	Black	Patient-facing: COVID wards throughout	0	0	0	0	0	1	Not an	Not ans	Not an	Not answ	Not answ	n/a	Positive		Positive	No
918	Female	18 – 30	Asian	Patient-facing: Mixed exposure	0	0	0	0	0	1	1	0	0	0	0	n/a	Negative		Negative	No
920	Male	41-50	White	Patient-facing: Mixed exposure	0	0	0	0	1	0	1	0	0	0	0	n/a	Negative		Negative	No
921	Female	51-60	Asian	Patient-facing: Non-COVID wards only	0	0	0	0	1	0	Not an	Not ans	Not an	Not answ	Not answ	n/a	Negative		Negative	No
922	Female	51-60	Black	Patient-facing: Mixed exposure	0	8	0	0	1	0	0	1	1	0	0	Moderate	Negative		Negative	No
923	Female	18 – 30	Asian	Patient-facing: Non-COVID wards only	14	2	1	0	0	0	2	0	0	0	0	Mild	Negative		Negative	Yes
924	Male	51-60	Prefer not to say	Patient-facing: COVID wards throughout	0	0	0	0	1	1	1	0	0	0	0	n/a	Negative		Negative	No
925	Male	31-40	Asian	Patient-facing: Mixed exposure	14	0	0	0	0	1	0	1	1	1	1	Moderate	Negative		Negative	Yes
926	Male	18 – 30	Asian	Patient-facing: Mixed exposure	0	0	0	0	1	0	1	0	0	0	0	n/a	Negative		Negative	Yes
927	Female	31-40	Asian	Patient-facing: COVID wards throughout	14	0	1	0	1	1	0	0	0	1	1	Mild	Positive		Positive	Yes
928	Male	41-50	Black	Patient-facing: Mixed exposure	0	17	0	0	1	0	0	0	1	1	0	Severe	Positive		Positive	Yes
930	Male	31-40	Asian	Patient-facing: Mixed exposure	0	22	0	0	1	0	Not an	Not ans	Not an	Not answ	Not answ	n/a	Positive	Positive	Positive	Not answered
931	Male	31-40	Asian	Patient-facing: Mixed exposure	0	0	1	0	0	1	0	0	0	0	1	Mild	Positive		Positive	Yes
932	Male	41-50	Black	Patient-facing: COVID wards throughout	0	21	0	0	1	0	Not an	Not ans	Not an	Not answ	Not answ	n/a	Negative	Not done	Negative	Not answered
933	Female	51-60	White	Patient-facing: Mixed exposure	0	0	0	0	1	0	1	0	0	0	0	n/a	Negative		Negative	No
934	Female	41-50	White	Patient-facing: COVID wards throughout	7	0	0	0	1	0	1	0	0	0	0	n/a	Negative		Negative	Yes
935	Female	41-50	Other	Patient-facing: Mixed exposure	0	0	0	0	1	0	0	1	1	1	1	Moderate	Positive		Positive	Yes
936	Female	18 – 30	Asian	Patient-facing: Non-COVID wards only	23	0	0	0	0	1	0	1	1	1	1	Moderate	Positive		Positive	Yes
937	Female	18 – 30	Other	Patient-facing: COVID wards throughout	0	0	0	0	0	1	1	0	0	0	0	n/a	Negative	Not done	Negative	No
939	Female	31-40	Asian	Patient-facing: Mixed exposure	14	30	0	0	1	0	0	1	1	1	1	Not answered	Positive		Positive	No
940	Female	41-50	Asian	Patient-facing: COVID wards throughout	0	15	0	0	1	0	0	0	1	1	1	Moderate	Positive	Not done	Positive	Yes
941	Female	31-40	Black	Patient-facing: Non-COVID wards only	0	0	1	0	1	1	1	0	0	0	0	n/a	Negative		Negative	Yes
942	Female	41-50	Asian	Patient-facing: COVID wards throughout	0	50	0	0	1	0	0	1	1	1	1	Moderate	Negative		Negative	No
943	Female	41-50	White	Patient-facing: Non-COVID wards only	0	0	0	0	1	0	1	0	0	0	0	n/a	Negative		Negative	No
944	Female	51-60	Black	Patient-facing: Mixed exposure	0	12	0	0	1	0	0	0	1	1	0	Moderate	Indeterminate		Indeterminate	Not answered
945	Female	18 – 30	White	Patient-facing: Mixed exposure	0	4	0	0	0	1	1	0	0	0	0	n/a	Negative		Negative	No
946	Female	18 – 30	Asian	Patient-facing: Mixed exposure	7	2	1	0	0	1	0	1	0	0	0	Moderate	Negative		Negative	No
947	Female	18 – 30	White	Non-patient facing: Non-clinical hospital staff	14	0	0	0	1	0	1	0	0	0	0	n/a	Negative		Negative	Yes
948	Female	18 – 30	Asian	Patient-facing: Mixed exposure	18	10	1	0	0	1	0	0	1	1	1	Moderate	Positive		Positive	Yes
949	Female	31-40	Not answered	Patient-facing: COVID wards throughout	0	0	1	0	1	0	1	0	0	0	0	n/a	Negative		Negative	No
950	Female	41-50	Asian	Patient-facing: Mixed exposure	14	14	0	0	1	0	0	1	1	1	0	Moderate	Positive		Positive	Yes
951	Male	18 – 30	White	Patient-facing: Mixed exposure	10	2	0	0	1	0	0	0	1	0	0	Mild	Positive		Positive	No
952	Female	51-60	White	Patient-facing: Non-COVID wards only	0	0	0	0	1	0	1	0	0	0	0	n/a	Negative		Negative	No
953	Male	18 – 30	Asian	Patient-facing: Mixed exposure	5	2	0	0	1	0	0	1	1	0	0	Mild	Positive		Positive	No
954	Male	18 – 30	Asian	Patient-facing: Non-COVID wards only	14	0	1	0	0	0	1	0	0	0	0	n/a	Negative		Negative	Yes
955	Male	51-60	White	Non-patient facing: Non-clinical hospital staff	0	0	1	1	1	1	1	0	0	0	0	n/a	Negative		Negative	No
956	Female	31-40	Asian	Patient-facing: Non-COVID wards only	0	0	1	0	0	1	1	0	0	0	0	n/a	Negative		Negative	No
957	Female	>60	White	Patient-facing: Non-COVID wards only	0	0	0	0	1	0	1	0	0	0	0	n/a	Negative	Not done	Negative	Not answered
958	Female	51-60	Black	Patient-facing: Mixed exposure	0	2	0	0	1	0	0	0	0	1	0	Not answered	Negative		Negative	No
959	Female	18 – 30	Black	Patient-facing: COVID wards throughout	14	0	0	0	1	0	0	1	1	0	0	Moderate	Positive		Positive	No
960	Male	18 – 30	Asian	Patient-facing: COVID wards throughout	8	0	0	0	1	1	1	0	0	0	0	n/a	Positive		Positive	No
961	Female	>60	Black	Patient-facing: Mixed exposure	0	0	0	0	1	0	1	0	0	0	0	n/a	Positive		Positive	No
962	Female	18 – 30	Asian	Patient-facing: Mixed exposure	14	0	0	0	0	1	0	0	0	1	1	Mild	Positive		Positive	Yes
963	Female	31-40	White	Patient-facing: Non-COVID wards only	0	0	0	0	0	1	1	0	0	0	0	n/a	Positive	Not done	Positive	No
964	Female	51-60	White	Non-patient facing: anon	0	4	0	0	1	0	1	0	0	0	0	n/a	Negative		Negative	Yes
966	Female	41-50	Asian	Patient-facing: Non-COVID wards only	0	14	0	0	1	1	0	1	0	1	1	Moderate	Positive		Positive	Yes
967	Male	41-50	White	Non-patient facing: Non-clinical hospital staff	14	0	0	0	1	0	1	0	0	0	0	n/a	Negative		Negative	Yes
968	Female	18 – 30	White	Patient-facing: Mixed exposure	14	0	0	0	0	1	1	0	0	0	0	n/a	Negative		Negative	Yes
969	Female	18 – 30	Asian	Patient-facing: Mixed exposure	17	13	0	0	0	1	0	1	0	1	1	Moderate	Positive		Positive	No
970	Male	31-40	White	Patient-facing: Non-COVID wards only	0	0	0	0	1	0	0	1	0	0	0	Moderate	Negative		Negative	Yes
971	Female	31-40	Asian	Patient-facing: Mixed exposure	14	0	0	0	0	1	1	0	0	0	0	n/a	Negative		Negative	Yes
972	Female	31-40	White	Patient-facing: Non-COVID wards only	7	0	1	0	1	0	0	1	1	0	0	Not answered	Negative		Negative	No
973	Female	18 – 30	Asian	Patient-facing: Mixed exposure	0	0	1	0	0	1	0	1	0	1	1	Moderate	Positive		Positive	Yes
974	Female	31-40	Asian	Patient-facing: Mixed exposure	0	0	0	0	1	0	1	0	0	0	0	n/a	Negative		Negative	Yes
975	Female	18 – 30	White	Patient-facing: Non-COVID wards only	14	5	0	0	1	0	0	0	1	0	0	Not answered	Negative		Negative	Yes
978	Female	51-60	White	Patient-facing: Mixed exposure	0	0	0	0	1	1	1	0	0	0	0	n/a	Positive		Positive	No
979	Female	31-40	White	Patient-facing: Mixed exposure	0	5	1	0	1	0	0	0	1	0	0	Moderate	Negative		Negative	No
980	Female	41-50	White	Patient-facing: COVID wards throughout	0	0	0	0	1	0	0	0	0	0	1	Mild	Negative		Negative	No
981	Female	31-40	Other	Patient-facing: COVID wards throughout	4	2	1	0	0	0	1	0	0	0	0	n/a	Positive		Positive	No
982	Male	41-50	White	Patient-facing: Non-COVID wards only	0	0	1	0	0	0	1	0	0	0	0	n/a	Negative		Negative	No
983	Female	41-50	Asian	Patient-facing: COVID wards throughout	60	0	0	0	1	0	0	1	0	1	1	Moderate	Positive		Positive	No

984	Female	41-50	White	Non-patient facing: Non-clinical hospital staff	0	0	1	0	1	0	1	0	0	0	0	n/a	Negative		Negative	No
985	Female	41-50	Asian	Patient-facing: COVID wards throughout	0	20	1	0	0	0	0	1	1	0	0	Severe	Positive		Positive	Yes
986	Female	18 – 30	Black	Non-patient facing: Non-clinical hospital staff	0	2	0	0	0	1	1	0	0	0	0	n/a	Negative		Negative	No
987	Female	51-60	White	Patient-facing: Non-COVID wards only	0	0	0	1	0	0	1	0	0	0	0	n/a	Negative		Negative	No
988	Male	51-60	Mixed	Patient-facing: Mixed exposure	0	0	0	0	1	0	1	0	0	0	0	n/a	Negative		Negative	No
989	Female	18 – 30	White	Patient-facing: Mixed exposure	0	0	0	0	1	0	0	0	0	1	1	Mild	Positive		Positive	Yes
990	Female	41-50	Asian	Patient-facing: COVID wards throughout	10	0	1	0	1	0	Not an	Not ans	Not an	Not answ	Not answ	n/a	Negative		Negative	Yes
991	Female	31-40	Asian	Patient-facing: Non-COVID wards only	5	0	0	0	0	1	Not an	Not ans	Not an	Not answ	Not answ	n/a	Positive		Positive	Yes
992	Female	31-40	White	Patient-facing: Mixed exposure	14	0	0	0	0	1	0	0	1	1	0	Moderate	Positive		Positive	Yes
993	Female	41-50	Asian	Patient-facing: Mixed exposure	0	0	0	0	1	1	1	0	0	0	0	n/a	Negative		Negative	No
994	Female	51-60	White	Non-patient facing: Non-clinical hospital staff	0	0	0	0	1	0	Not an	Not ans	Not an	Not answ	Not answ	n/a	Negative		Negative	No
995	Female	41-50	White	Patient-facing: Mixed exposure	7	20	0	0	1	0	0	1	0	1	1	Moderate	Positive		Positive	No
996	Female	18 – 30	White	Patient-facing: Non-COVID wards only	0	10	0	0	1	1	1	0	0	0	0	n/a	Negative		Negative	No
997	Female	41-50	White	Patient-facing: Non-COVID wards only	21	0	0	0	1	0	0	0	1	1	1	Moderate	Positive		Positive	No
998	Female	41-50	White	Patient-facing: COVID wards throughout	5	0	0	0	1	0	1	0	0	0	0	n/a	Positive		Positive	No
1000	Female	31-40	White	Patient-facing: Non-COVID wards only	7	0	0	0	1	0	0	1	0	1	1	Mild	Positive		Positive	Yes
1001	Female	41-50	Black	Patient-facing: COVID wards throughout	0	0	0	0	1	0	1	0	0	0	0	n/a	Positive		Positive	No
1002	Female	31-40	Asian	Patient-facing: COVID wards throughout	0	7	0	0	0	1	2	0	0	0	0	Mild	Negative		Negative	Yes
1003	Female	31-40	White	Patient-facing: Non-COVID wards only	0	0	0	0	1	0	1	0	0	0	0	n/a	Negative		Negative	No
1004	Female	41-50	Asian	Patient-facing: Non-COVID wards only	7	0	0	0	1	0	Not an	Not ans	Not an	Not answ	Not answ	n/a	Positive	Negative	Positive	Not answered
1005	Female	31-40	Asian	Patient-facing: COVID wards throughout	11	8	0	0	0	1	0	0	1	1	1	Moderate	Positive		Positive	No
1006	Female	18 – 30	Asian	Patient-facing: COVID wards throughout	0	0	1	0	0	0	1	0	0	0	0	n/a	Negative		Negative	No
1007	Female	31-40	White	Patient-facing: Non-COVID wards only	3	0	0	0	1	0	1	0	0	0	0	n/a	Negative		Negative	Yes
1008	Male	31-40	Black	Patient-facing: Mixed exposure	0	14	0	0	1	0	0	1	0	0	0	Moderate	Negative		Negative	No
1009	Female	31-40	Asian	Patient-facing: Mixed exposure	12	0	0	0	0	1	0	1	1	0	0	Mild	Positive		Positive	Yes
1010	Male	31-40	White	Patient-facing: Non-COVID wards only	0	0	0	0	1	0	1	0	0	0	0	n/a	Negative		Negative	No
1012	Male	18 – 30	White	Patient-facing: Non-COVID wards only	0	0	0	0	1	0	1	0	0	0	0	n/a	Negative		Negative	No
1013	Female	41-50	Asian	Patient-facing: COVID wards throughout	0	0	0	0	1	0	1	0	0	0	0	n/a	Negative		Negative	No
1014	Female	>60	White	Patient-facing: Mixed exposure	40	28	1	0	1	0	0	1	0	1	1	Mild	Positive		Positive	No
1015	Female	18 – 30	Asian	Patient-facing: Non-COVID wards only	14	0	1	0	0	1	1	0	0	0	0	n/a	Negative		Negative	Yes
1016	Female	51-60	White	Patient-facing: Non-COVID wards only	0	0	0	0	1	0	1	0	0	0	0	n/a	Negative		Negative	No
1017	Male	41-50	Asian	Patient-facing: Mixed exposure	7	0	0	0	1	0	0	1	1	0	0	Mild	Negative		Negative	No
1018	Female	41-50	White	Patient-facing: Non-COVID wards only	0	1	0	0	1	0	1	0	0	0	0	n/a	Negative		Negative	No
1019	Female	41-50	Asian	Patient-facing: COVID wards throughout	0	0	0	0	1	0	1	0	0	0	0	n/a	Negative		Negative	No
1020	Female	>60	White	Patient-facing: Non-COVID wards only	6	0	0	0	1	0	1	0	0	0	0	n/a	Negative		Negative	Yes
1021	Female	31-40	Asian	Patient-facing: COVID wards throughout	14	0	0	0	1	0	2	0	0	0	0	Mild	Positive		Positive	Yes
1022	Female	18 – 30	White	Patient-facing: Non-COVID wards only	21	0	0	0	1	0	0	1	1	1	1	Moderate	Positive		Positive	No
1023	Female	18 – 30	Black	Patient-facing: Non-COVID wards only	7	0	0	0	1	0	0	1	0	0	0	Mild	Negative		Negative	No
1024	Female	41-50	Asian	Patient-facing: Mixed exposure	7	0	1	0	0	0	0	0	1	0	0	Mild	Negative		Negative	No
1025	Female	31-40	White	Patient-facing: COVID wards throughout	14	0	1	0	1	0	1	0	0	0	0	n/a	Negative		Negative	Yes
1026	Female	51-60	Asian	Patient-facing: COVID wards throughout	0	0	1	0	0	0	1	0	0	0	0	n/a	Negative		Negative	No
1027	Male	41-50	Asian	Patient-facing: Mixed exposure	0	0	0	0	1	0	1	0	0	0	0	n/a	Negative		Negative	No
1028	Male	31-40	Asian	Non-patient facing: anon	0	0	1	0	0	1	1	0	0	0	0	n/a	Negative		Negative	No
1029	Female	51-60	White	Patient-facing: COVID wards throughout	0	0	0	0	0	1	0	0	0	1	1	Severe	Positive		Positive	Yes
1030	Female	51-60	White	Non-patient facing: Non-clinical hospital staff	0	0	0	0	1	0	Not an	Not ans	Not an	Not answ	Not answ	n/a	Negative		Negative	No
1032	Female	18 – 30	White	Patient-facing: Mixed exposure	0	14	0	0	1	0	0	1	1	1	1	Moderate	Positive		Positive	No
1033	Female	18 – 30	Asian	Patient-facing: Mixed exposure	0	0	0	0	0	1	1	0	0	0	0	n/a	Negative		Negative	No
1034	Female	31-40	Asian	Patient-facing: Mixed exposure	0	35	0	0	1	0	0	1	1	1	1	Moderate	Positive		Positive	Yes
1035	Female	31-40	Asian	Patient-facing: Mixed exposure	0	0	1	0	0	1	1	0	0	0	0	n/a	Negative	Not done	Negative	Yes
1036	Female	>60	White	Patient-facing: Non-COVID wards only	7	0	1	0	0	0	0	1	0	0	0	Mild	Negative		Negative	Yes
1037	Female	51-60	Asian	Patient-facing: Non-COVID wards only	10	0	0	0	1	0	Not an	Not ans	Not an	Not answ	Not answ	n/a	Positive	Positive	Positive	Not answered
1038	Female	31-40	White	Patient-facing: Non-COVID wards only	5	0	0	0	1	0	0	1	0	0	0	Mild	Negative		Negative	No
1039	Female	31-40	White	Patient-facing: COVID wards throughout	0	0	0	0	1	0	0	1	0	1	1	Moderate	Negative		Negative	No
1040	Female	>60	White	Non-patient facing: anon	0	0	0	0	1	0	1	0	0	0	0	n/a	Negative		Negative	No
1041	Female	41-50	Asian	Patient-facing: Non-COVID wards only	14	0	0	0	1	0	1	0	0	0	0	n/a	Negative		Negative	Yes
1042	Female	41-50	Asian	Patient-facing: COVID wards throughout	0	17	0	0	1	0	0	1	1	1	1	Moderate	Positive		Positive	Yes
1043	Female	51-60	White	Non-patient facing: anon	0	0	0	0	1	0	1	0	0	0	0	n/a	Negative		Negative	No
1044	Female	41-50	White	Patient-facing: Mixed exposure	0	3	0	0	1	0	1	0	0	0	0	n/a	Positive	Negative	Positive	No
1045	Female	18 – 30	White	Patient-facing: COVID wards throughout	0	0	0	0	1	0	1	0	0	0	0	n/a	Positive		Positive	No
1046	Female	18 – 30	Asian	Non-patient facing: anon	14	0	0	0	1	1	0	0	1	0	0	Mild	Negative		Negative	Yes
1047	Female	51-60	Asian	Patient-facing: Mixed exposure	14	0	0	0	1	0	1	0	0	0	0	n/a	Negative		Negative	Not answered
1048	Female	31-40	Prefer not to say	Non-patient facing: anon	0	0	0	0	1	0	Not an	Not ans	Not an	Not answ	Not answ	n/a	Negative	Not done	Negative	Not answered
1049	Female	41-50	White	Non-patient facing: Non-clinical hospital staff	14	14	0	0	1	0	0	1	1	1	1	Severe	Positive		Positive	No
1050	Female	41-50	White	Non-patient facing: anon	0	5	0	0	1	0	0	1	1	0	1	Moderate	Negative	Not done	Negative	Yes
1051	Female	51-60	White	Non-patient facing: Non-clinical hospital staff	0	28	0	0	1	0	0	1	1	0	0	Moderate	Negative		Negative	Not answered
1052	Female	41-50	White	Non-patient facing: Non-clinical hospital staff	0	0	0	0	1	0	1	0	0	0	0	n/a	Positive		Positive	No
1053	Female	31-40	White	Non-patient facing: anon	0	0	0	0	1	0	1	0	0	0	0	n/a	Negative		Negative	Yes
1054	Female	41-50	Asian	Patient-facing: Non-COVID wards only	0	0	0	0	1	0	1	0	0	0	0	n/a	Positive		Positive	No
1055	Female	51-60	White	Non-patient facing: Non-clinical hospital staff	0	0	0	0	0	1	1	0	0	0	0	n/a	Negative		Negative	No
1056	Female	18 – 30	Asian	Patient-facing: COVID wards throughout	14	7	0	0	0	1	0	1	1	1	1	Moderate	Negative		Negative	Yes
1057	Female	41-50	Asian	Patient-facing: Mixed exposure	0	0	1	0	0	1	1	0	0	0	0	n/a	Negative		Negative	No
1058	Male	41-50	Black	Non-patient facing: anon	0	0	0	0	1	1	0	1	1	1	1	Moderate	Negative		Negative	Yes
1059	Female	18 – 30	Asian	Patient-facing: COVID wards throughout	14	0	1	0	0	0	1	0	0	0	0	n/a	Negative		Negative	No
1060	Female	31-40	Black	Patient-facing: Mixed exposure	0	5	0	0	0	1	0	1	0	0	0	Severe	Negative		Negative	Yes
1061	Male	18 – 30	Asian	Patient-facing: Non-COVID wards only	0	0	0	0	1	0	1	0	0	0	0	n/a	Negative		Negative	No
1062	Female	>60	White	Patient-facing: Non-COVID wards only	0	0	1	0	1	0	1	0	0	0	0	n/a	Negative		Negative	No
1063	Female	41-50	Not answered	Patient-facing: COVID wards throughout	14	0	1	0	1	0	0	0	1	0	0	Mild	Negative		Negative	No
1064	Female	41-50	Black	Patient-facing: COVID wards throughout	0	0	0	0	0	1	1	0	0	0	0	n/a	Positive		Positive	No
1065	Male	31-40	Other	Patient-facing: Mixed exposure	0	0	0	0	0	1	1	0	0	0	0	n/a	Negative		Negative	No
1066	Female	18 – 30	Other	Patient-facing: COVID wards throughout	14	5	0	0	0	1	0	1	0	0	1	Mild	Negative		Negative	Yes
1068	Female	41-50	White	Patient-facing: COVID wards throughout	0	0	0	0	1	0	1	0	0	0	0	n/a	Negative		Negative	Yes
1069	Female	51-60	White	Patient-facing: COVID wards throughout	0	0	0	0	1	0	1	0	0	0	0	n/a	Negative		Negative	No
1070	Female	>60	White	Patient-facing: Mixed exposure	0	0	0	0	0	1	1	0	0	0	0	n/a	Negative		Negative	No
1071	Female	18-30	White	Patient-facing: Non-COVID wards only	14	0	1	0	0	0	1	0	0	0	0	n/a	Negative		Negative	Yes
1072	Female	31-40	White	Non-patient facing: Non-clinical hospital staff	0	0	0	0	1	0	1	0	0	0	0	n/a	Positive		Positive	No
1073	Female	18-30	White	Non-patient facing: Non-clinical hospital staff	0	5	0	0	1	0	1	0	0	0	0	n/a	Negative		Negative	No
1074	Female	41-50	White	Patient-facing: Non-COVID wards only	4	0	0	0	0	1	0	1	0	0	0	Moderate	Negative		Negative	No
1075	Female	41-50	White	Non-patient facing: Non-clinical hospital staff	0	0	0	0	1	0	1	0	0	0	0	n/a	Negative		Negative	Yes
1076	Female	31-40	White	Non-patient facing: Non-clinical hospital staff	0	0	0	0	1	0	1	0	0	0	0	n/a	Negative		Negative	No
1077	Male	>60	White	Patient-facing: Non-COVID wards only	0	0	0	0	1	0	1	0	0	0	0	n/a	Negative		Negative	No
1078	Male	18-30	White	Non-patient facing: Non-clinical hospital staff	0	0	1	0	1	1	1	0	0	0	0	n/a	Negative		Negative	Yes
1079	Female	41-50	White	Non-patient facing: Non-clinical hospital staff	0	14	0	0	1	0	1	0	0	0	0	n/a	Negative		Negative	No
1080	Female	41-50	White	Patient-facing: Non-COVID wards only	0	0	0	0	1	0	1	0	0	0	0	n/a	Negative		Negative	No
1081	Female	18-30	White	Patient-facing: Mixed exposure	7	30	0	0	1	1	Not an	Not answ	Not an	Not answ	Not answ	n/a	Positive	Positive	Positive	Not answered
1082	Male	18-30	Black	Patient-facing: Non-COVID wards only	14	10	0	0	1	0	0	1	1	1	1	Severe	Positive		Positive	Yes

1083	Female	51-60	White	Patient-facing: Mixed exposure	47	0	0	0	1	0	1	0	0	0	0	n/a	Negative		Negative	No
1084	Female	>60	White	Patient-facing: Non-COVID wards only	0	0	1	0	1	0	1	0	0	0	0	n/a	Negative		Negative	No
1085	Female	18-30	Other	Patient-facing: Non-COVID wards only	0	0	1	0	0	1	1	0	0	0	0	n/a	Negative		Negative	No
1086	Male	>60	White	Patient-facing: COVID wards throughout	0	0	1	0	1	0	1	0	0	0	0	n/a	Negative		Negative	No
1087	Female	51-60	White	Patient-facing: Non-COVID wards only	0	0	1	0	0	0	1	0	0	0	0	n/a	Negative		Negative	Yes
1088	Female	41-50	White	Non-patient facing: Non-clinical hospital staff	0	0	0	0	0	1	1	0	0	0	0	n/a	Negative		Negative	No
1089	Female	51-60	White	Patient-facing: Non-COVID wards only	0	0	1	0	0	0	Not an	Not ans	Not an	Not answ	Not answ	n/a	Negative	Not done	Negative	Not answered
1090	Female	18-30	White	Patient-facing: Non-COVID wards only	0	8	0	0	0	1	0	0	1	1	1	Mild	Positive		Positive	Yes
1091	Female	51-60	White	Patient-facing: Non-COVID wards only	0	0	0	0	0	1	1	0	0	0	0	n/a	Negative		Negative	No
1092	Female	31-40	White	Non-patient facing: Non-clinical hospital staff	0	0	0	0	1	0	0	1	1	1	0	Moderate	Negative		Negative	Yes
1093	Female	31-40	White	Non-patient facing: Non-clinical hospital staff	2	0	0	0	1	0	1	0	0	0	0	n/a	Negative		Negative	No
1094	Female	41-50	Black	Patient-facing: Mixed exposure	0	0	Not answered	Not answered	Not answered	Not answered	1	0	0	0	0	n/a	Negative		Negative	No
1095	Female	51-60	White	Patient-facing: Non-COVID wards only	0	27	0	0	1	0	1	0	0	0	0	n/a	Negative		Negative	No
1096	Female	51-60	White	Patient-facing: Non-COVID wards only	8	0	0	0	1	0	0	1	1	1	1	Moderate	Positive		Positive	No
1097	Female	31-40	White	Patient-facing: Non-COVID wards only	0	0	0	0	1	0	0	0	0	1	1	Mild	Positive		Positive	No
1098	Female	51-60	Not answered	Patient-facing: Mixed exposure	5	0	0	0	1	0	1	0	0	0	0	n/a	Positive		Positive	No
1099	Female	51-60	Black	Patient-facing: Non-COVID wards only	14	20	0	0	1	1	Not an	Not ans	Not an	Not answ	Not answ	n/a	Positive		Positive	No
1100	Female	51-60	White	Patient-facing: Non-COVID wards only	Not answered	Not answered	0	0	1	0	1	0	0	0	0	n/a	Negative		Negative	No
1101	Female	18-30	White	Patient-facing: Mixed exposure	0	0	0	0	0	1	1	0	0	0	0	n/a	Negative		Negative	No
1102	Female	51-60	White	Patient-facing: COVID wards throughout	7	11	1	0	0	1	0	0	0	1	1	Moderate	Positive		Positive	No
1103	Female	51-60	White	Patient-facing: Non-COVID wards only	0	0	0	0	1	0	0	0	0	1	1	Moderate	Negative		Negative	Yes
1104	Female	51-60	Asian	Patient-facing: Non-COVID wards only	14	28	0	0	0	1	0	0	1	0	0	Mild	Negative		Negative	No
1105	Female	31-40	Asian	Patient-facing: COVID wards throughout	14	0	1	0	0	0	0	0	0	1	1	Moderate	Negative		Negative	No
1107	Female	51-60	White	Patient-facing: Mixed exposure	0	0	0	0	1	0	1	0	0	0	0	n/a	Positive		Positive	No
1109	Female	18-30	Black	Patient-facing: Non-COVID wards only	0	3	0	0	0	1	1	0	0	0	0	n/a	Positive		Positive	No
1111	Female	18-30	White	Patient facing: Unknown	12	0	0	0	1	0	0	0	0	1	1	Moderate	Positive		Positive	No
1113	Female	31-40	Asian	Patient-facing: Non-COVID wards only	7	7	1	0	0	1	0	0	1	0	0	Moderate	Positive		Positive	Yes
1116	Female	51-60	Black	Patient-facing: Non-COVID wards only	14	Not answered	0	0	1	0	0	1	1	1	1	Moderate	Positive		Positive	Yes
1117	Female	51-60	Black	Patient facing: Unknown	7	4	0	0	1	0	0	1	0	0	0	Severe	Positive		Positive	Yes
1118	Female	41-50	White	Patient-facing: Mixed exposure	0	37	0	0	1	0	0	1	1	0	0	Severe	Positive		Positive	No
1122	Female	41-50	Black	Patient-facing: Mixed exposure	0	0	0	0	1	0	0	0	0	1	1	Mild	Positive		Positive	No
1124	Female	51-60	Black	Patient-facing: Non-COVID wards only	0	0	0	0	1	0	1	0	0	0	0	n/a	Positive		Positive	No
1125	Female	51-60	White	Patient-facing: Non-COVID wards only	0	5	0	0	1	0	1	0	0	0	0	n/a	Negative		Negative	Yes
1126	Female	41-50	Other	Patient-facing: Mixed exposure	0	20	0	0	1	0	0	1	1	1	1	Moderate	Positive		Positive	Yes
1127	Female	41-50	White	Patient-facing: Mixed exposure	14	0	0	0	1	0	1	0	0	0	0	n/a	Negative		Negative	Yes
1128	Female	31-40	White	Non-patient facing: Non-clinical hospital staff	7	Not answered	0	0	1	0	0	0	1	0	0	Mild	Positive		Positive	No
1129	Male	41-50	Asian	Non-patient facing: Non-clinical hospital staff	0	0	0	0	1	0	1	0	0	0	0	n/a	Positive		Positive	No
1130	Male	41-50	White	Non-patient facing: Non-clinical hospital staff	0	7	0	0	1	0	1	0	0	0	0	n/a	Negative		Negative	No
1131	Female	51-60	White	Non-patient facing: Non-clinical hospital staff	2	0	0	0	1	0	2	0	0	0	0	Mild	Negative		Negative	No
1132	Female	51-60	White	Non-patient facing: Non-clinical hospital staff	50	8	0	0	1	0	0	1	1	0	0	Mild	Negative		Negative	Yes
1133	Female	41-50	Black	Patient facing: Unknown	0	0	0	0	0	1	0	0	0	1	1	Severe	Positive		Positive	Not answered
1134	Male	18 – 30	White	Non-patient facing: Non-clinical hospital staff	14	Not answered	0	0	1	0	0	1	0	0	0	Moderate	Negative		Negative	Yes
1135	Male	31-40	White	Non-patient facing: Non-clinical hospital staff	12	Not answered	0	0	1	0	0	1	1	1	1	Moderate	Negative		Negative	No
1136	Female	>60	Black	Non-patient facing: anon	Not answered	14	0	0	1	0	1	0	0	0	0	n/a	Negative		Negative	Yes
1137	Female	18 – 30	White	Non-patient facing: Non-clinical hospital staff	0	0	0	0	0	1	1	0	0	0	0	n/a	Negative		Negative	No
1138	Female	18 – 30	White	Patient-facing: Non-COVID wards only	0	0	0	0	1	0	1	0	0	0	0	n/a	Negative		Negative	No
1139	Female	18 – 30	White	Patient-facing: Mixed exposure	0	0	0	0	1	0	1	0	0	0	0	n/a	Positive		Positive	No
1140	Female	31-40	White	Non-patient facing: Non-clinical hospital staff	0	0	0	0	1	1	1	0	0	0	0	n/a	Negative		Negative	Yes
1141	Male	18 – 30	White	Patient-facing: Mixed exposure	0	0	0	0	1	1	1	0	0	0	0	n/a	Negative		Negative	No
1142	Female	31-40	Asian	Patient-facing: Non-COVID wards only	0	0	0	0	1	0	0	0	1	0	0	Mild	Negative		Negative	No
1143	Female	31-40	White	Patient-facing: Non-COVID wards only	Not answered	Not answered	1	0	1	0	0	0	0	1	1	Mild	Negative		Negative	Yes
1144	Female	18 – 30	White	Patient-facing: Mixed exposure	7	4	0	1	1	1	0	1	0	0	0	Mild	Positive		Positive	Not answered
1145	Male	51-60	White	Non-patient facing: Non-clinical hospital staff	0	0	0	0	1	0	1	0	0	0	0	n/a	Positive		Positive	No
1146	Female	18 – 30	Black	Non-patient facing: anon	0	0	0	0	1	1	1	0	0	0	0	n/a	Negative		Negative	Yes
1147	Female	18-30	White	Non-patient facing: anon	Not answered	14	0	0	1	0	1	0	0	0	0	n/a	Negative		Negative	No
1148	Female	18-30	White	Patient-facing: Non-COVID wards only	Not answered	12	0	0	0	1	0	0	0	1	1	Moderate	Negative		Negative	No
1149	Female	41-50	White	Non-patient facing: Non-clinical hospital staff	0	0	0	0	1	0	1	0	0	0	0	n/a	Negative		Negative	No
1150	Female	31-40	White	Non-patient facing: Non-clinical hospital staff	0	0	0	0	1	0	2	0	0	0	0	Mild	Negative		Negative	No
1151	Male	41-50	Asian	Non-patient facing: Non-clinical hospital staff	14	14	0	0	1	0	0	0	1	1	1	Moderate	Negative		Negative	Yes
1152	Male	41-50	White	Patient-facing: Mixed exposure	0	0	1	0	0	0	1	0	0	0	0	n/a	Negative		Negative	No
1153	Female	18 – 30	Black	Patient-facing: Mixed exposure	14	10	0	0	0	1	0	0	0	1	1	Severe	Negative		Negative	No
1154	Female	31-40	White	Patient-facing: Mixed exposure	7	Not answered	0	0	1	0	0	1	1	0	0	Mild	Negative		Negative	No
1155	Female	41-50	Asian	Patient-facing: Mixed exposure	7	0	0	0	0	1	1	0	0	0	0	n/a	Positive		Positive	No
1156	Female	51-60	White	Non-patient facing: anon	0	0	0	0	1	0	1	0	0	0	0	n/a	Negative		Negative	No
1157	Female	41-50	White	Patient-facing: Non-COVID wards only	0	10	0	0	1	0	1	0	0	0	0	n/a	Negative		Negative	No
1158	Male	51-60	White	Patient-facing: Mixed exposure	Not answered	7	0	0	1	0	0	1	0	0	0	Moderate	Negative		Negative	Yes
1159	Female	18 – 30	Mixed	Patient-facing: Non-COVID wards only	14	0	0	0	1	1	0	0	0	1	1	Moderate	positive		Positive	Yes
1160	Female	41-50	White	Non-patient facing: Non-clinical hospital staff	10	Not answered	0	0	1	0	1	0	0	0	0	n/a	Negative		Negative	Yes
1161	Female	18 – 30	Black	Non-patient facing: Non-clinical hospital staff	0	0	0	0	1	0	1	0	0	0	0	n/a	Positive		Positive	No
1162	Female	18 – 30	Black	Patient-facing: Mixed exposure	0	4	1	0	0	0	1	0	0	0	0	n/a	Negative		Negative	No
1163	Female	51-60	Black	Patient-facing: Non-COVID wards only	0	0	0	0	1	0	1	0	0	0	0	n/a	Negative		Negative	No
1164	Female	51-60	White	Patient-facing: Non-COVID wards only	0	0	1	0	0	0	1	0	0	0	0	n/a	Positive		Positive	No
1165	Female	31-40	Asian	Non-patient facing: anon	0	0	1	0	0	1	1	0	0	0	0	n/a	Negative		Negative	No
1166	Female	51-60	White	Non-patient facing: Non-clinical hospital staff	3	Not answered	0	0	0	1	1	0	0	0	0	n/a	Negative		Negative	No
1167	Female	41-50	Asian	Non-patient facing: Non-clinical hospital staff	0	0	1	0	1	0	1	0	0	0	0	n/a	Negative		Negative	No
1168	Female	41-50	White	Non-patient facing: Non-clinical hospital staff	0	0	0	0	1	0	1	0	0	0	0	n/a	Negative		Negative	No
