# Supplementary material for: High numbers of COVID-19 patients transit through non-COVID wards, and associated healthcare workers have high infection rates: An observational cross-sectional study
Source: PLoS One. 2022 Oct 19;17(10):e0275154. doi: 10.1371/journal.pone.0275154 (PMC9581418; doi:10.1371/journal.pone.0275154)
Supplement: Attachment — Submitted filename: PONE-D-21-07362_R2.pdf [file pone.0275154.s010.pdf]

PLOS ONE

High numbers of COVID-19 patients transit through non-COVID wards, and associated healthcare workers have high infection rates: An observational cross-sectional study

--Manuscript Draft--

**Manuscript Number:**	PONE-D-21-07362R2
**Article Type:**	Research Article
**Full Title:**	High numbers of COVID-19 patients transit through non-COVID wards, and associated healthcare workers have high infection rates: An observational cross-sectional study
**Short Title:**	COVID-19 patients on non-COVID wards
**Corresponding Author:**	Stella Shee<br>King's College Hospital NHS Trust: King's College Hospital NHS Foundation Trust<br>London, Kent UNITED KINGDOM
**Keywords:**	: covid-19; SARS-CoV-2 antibody; Healthcare workers; non-COVID area; infection risk, personal protective equipment
**Abstract:**	Infection risk is high in healthcare workers working with COVID-19 patients but the risk in non-COVID clinical environments is less clear. We measured infection rates early in the pandemic by SARS-CoV-2 antibody and/or a positive PCR test in 1118 HCWs within various hospital environments with particular focus on non-COVID clinical areas. Infection risk on non-COVID wards was estimated through the surrogate metric of numbers of patients transferred from a non-COVID to a COVID ward. Staff infection rates increased with likelihood of COVID exposure and suggested high risk in non-COVID clinical areas (non patient-facing 23.2% versus patient-facing in either non-COVID environments 31.5% or COVID wards 44%). High numbers of patients admitted to COVID wards had initially been admitted to designated non-COVID wards (22-48% at peak). Infection risk was high during a pandemic in all clinical environments and non-COVID designation may provide false reassurance. Our findings support the need for common personal protective equipment standards in all clinical areas, irrespective of COVID/non-COVID designation.
**Order of Authors:**	Susanna Nallamilli
	Tejus Patel
	April Buazon
	Jennifer Vidler
	Sam Norton
	Mustafa Atta
	James Galloway
	Stella Bowcock
**Response to Reviewers:**	Dear Editors,<br>Thank you for reconsidering our manuscript which was provisionally accepted for publication in June 2021, but then rejected on a concern about ethics approval. This has been reviewed by a senior editor who is now satisfied that we followed correct procedures and can resubmit the manuscript.We now resubmit a clean version of the manuscript and one with tracked changes made since the original peer review. The changes are mainly to the methods section clarifying the ethics and consent processes.<br><br>We very much hope that you are now able to accept the manuscript as it has already passed peer review.<br><br>With many thanks,
**Additional Information:**	
**Question**	**Response**

**Financial Disclosure**<br><br>Enter a financial disclosure statement that describes the sources of funding for the work included in this submission. Review the submission guidelines for detailed requirements. View published research articles from *PLOS ONE* for specific examples.<br><br>This statement is required for submission and **will appear in the published article** if the submission is accepted. Please make sure it is accurate.<br><br>**Unfunded studies**<br>Enter: *The author(s) received no specific funding for this work.*<br><br>**Funded studies**<br>Enter a statement with the following details:<br>• Initials of the authors who received each award<br>• Grant numbers awarded to each author<br>• The full name of each funder<br>• URL of each funder website<br>• Did the sponsors or funders play any role in the study design, data collection and analysis, decision to publish, or preparation of the manuscript?<br>• **NO** - Include this sentence at the end of your statement: *The funders had no role in study design, data collection and analysis, decision to publish, or preparation of the manuscript.*<br>• **YES** - Specify the role(s) played.<br><br>* typeset	The author(s) received no specific funding for this work
**Competing Interests**<br><br>Use the instructions below to enter a competing interest statement for this submission. On behalf of all authors, disclose any competing interests that could be perceived to bias this work—acknowledging all financial support and any other relevant financial or non-financial competing interests.<br><br>This statement is required for submission	The authors have declared that no competing interests exist.

and **will appear in the published article** if the submission is accepted. Please make sure it is accurate and that any funding sources listed in your Funding Information later in the submission form are also declared in your Financial Disclosure statement.<br><br>View published research articles from *PLOS ONE* for specific examples.<br><br>**NO authors have competing interests**<br><br>Enter: *The authors have declared that no competing interests exist.*<br><br>**Authors with competing interests**<br><br>Enter competing interest details beginning with this statement:<br><br>*I have read the journal's policy and the authors of this manuscript have the following competing interests: [insert competing interests here]*<br><br>* typeset	
**Ethics Statement**<br><br>Enter an ethics statement for this submission. This statement is required if the study involved:<br><br>• Human participants<br>• Human specimens or tissue<br>• Vertebrate animals or cephalopods<br>• Vertebrate embryos or tissues<br>• Field research<br><br>Write "N/A" if the submission does not require an ethics statement.<br><br>General guidance is provided below. Consult the submission guidelines for detailed instructions. **Make sure that all information entered here is included in the Methods section of the manuscript.**	Approval for this audit was not necessary as this was a service evaluation and permission was granted by the trust management board. Blood testing was done under the national NHS England & Improvement voluntary healthcare staff testing programme and took place independently of this audit. Written informed consent was obtained to access staff SARS-CoV-2 PCR and serology results.<br>The ethics and consent part of this service evaluation have been reviewed by a senior editor and accepted that appropriate procedures were followed.

**Format for specific study types**<br><br>**Human Subject Research (involving human participants and/or tissue)**<br>• Give the name of the institutional review board or ethics committee that approved the study<br>• Include the approval number and/or a statement indicating approval of this research<br>• Indicate the form of consent obtained (written/oral) or the reason that consent was not obtained (e.g. the data were analyzed anonymously)<br><br>**Animal Research (involving vertebrate animals, embryos or tissues)**<br>• Provide the name of the Institutional Animal Care and Use Committee (IACUC) or other relevant ethics board that reviewed the study protocol, and indicate whether they approved this research or granted a formal waiver of ethical approval<br>• Include an approval number if one was obtained<br>• If the study involved *non-human primates*, add *additional details* about animal welfare and steps taken to ameliorate suffering<br>• If anesthesia, euthanasia, or any kind of animal sacrifice is part of the study, include briefly which substances and/or methods were applied<br><br>**Field Research**<br><br>Include the following details if this study involves the collection of plant, animal, or other materials from a natural setting:<br>• Field permit number<br>• Name of the institution or relevant body that granted permission	
**Data Availability**<br><br>Authors are required to make all data underlying the findings described fully available, without restriction, and from the time of publication. PLOS allows rare exceptions to address legal and ethical concerns. See the PLOS Data Policy and FAQ for detailed information.	Yes - all data are fully available without restriction

A Data Availability Statement describing where the data can be found is required at submission. Your answers to this question constitute the Data Availability Statement and **will be published in the article**, if accepted.<br><br>**Important:** Stating 'data available on request from the author' is not sufficient. If your data are only available upon request, select 'No' for the first question and explain your exceptional situation in the text box.<br><br>Do the authors confirm that all data underlying the findings described in their manuscript are fully available without restriction?	
**Describe where the data may be found in full sentences. If you are copying our sample text, replace any instances of XXX with the appropriate details.**<br><br>• If the data are **held or will be held in a public repository**, include URLs, accession numbers or DOIs. If this information will only be available after acceptance, indicate this by ticking the box below. For example: *All XXX files are available from the XXX database (accession number(s) XXX, XXX.*).<br>• If the data are all contained **within the manuscript and/or Supporting Information files**, enter the following: *All relevant data are within the manuscript and its Supporting Information files.*<br>• If neither of these applies but you are able to provide **details of access elsewhere**, with or without limitations, please do so. For example:<br><br>*Data cannot be shared publicly because of [XXX]. Data are available from the XXX Institutional Data Access / Ethics Committee (contact via XXX) for researchers who meet the criteria for access to confidential data.*<br><br>*The data underlying the results presented in the study are available from (include the name of the third party*	All relevant data are within the manuscript and its Supporting Information files

and contact information or URL).<br>• This text is appropriate if the data are owned by a third party and authors do not have permission to share the data.<br><br>* typeset	
Additional data availability information:	

Powered by Editorial Manager® and ProduXion Manager® from Aries Systems Corporation

Manuscript

Click here to access/download;Manuscript;SARS-CoV-2 infecn non-covid wards.v3.7.plos one_clean.docx

Full title: High numbers of COVID-19 patients transit through non-COVID wards, and associated healthcare workers have high infection rates: An observational cross-sectional study

Short title: COVID-19 patients on non-COVID wards

Susanna Nallamilli[1], Tejus Patel[1], April Buazon[2], Jennifer Vidler[3], Sam Norton[4], Mustafa Atta[2], James Galloway[4] and Stella Bowcock[3]

[1] Department of Medicine, Princess Royal University Hospital, King's College Hospital NHS Trust, Kent, [2] Department of Microbiology, Princess Royal University Hospital, King's College Hospital NHS Trust, Kent, [3]Department of Haematology, Princess Royal University Hospital, King's College Hospital NHS Trust, Kent, [4]Centre for Rheumatic Disease, King's College London, London

Abstract

Infection risk is high in healthcare workers working with COVID-19 patients but the risk in non-COVID clinical environments is less clear. We measured infection rates early in the pandemic by SARS-CoV-2 antibody and/or a positive PCR test in 1118 HCWs within various hospital environments with particular focus on non-COVID clinical areas. Infection risk on non-COVID wards was estimated through the surrogate metric of numbers of patients transferred from a non-COVID to a COVID ward. Staff infection rates increased with likelihood of COVID exposure and suggested high risk in non-COVID clinical areas (non patient-facing 23.2% versus patient-facing in either non-COVID environments 31.5% or COVID wards 44%). High numbers of patients admitted to COVID wards had initially been admitted to designated non-COVID wards (22-48% at peak). Infection risk was high during a pandemic in all clinical environments and non-COVID designation may provide false reassurance. Our findings support the need for common personal protective equipment standards in all clinical areas, irrespective of COVID/non-COVID designation.

Introduction

The severe acute respiratory syndrome coronavirus 2 (SARS-CoV-2) swept the world rapidly in early 2020, before we had fully understood the transmission characteristics and illness spectrum caused by

the virus [1]. Early in the pandemic, testing capacity for the virus was limited, turnaround slow and the test was insufficiently sensitive to enable effective separation of patients at the point of admission [2]. The separation of patients into non-COVID and COVID areas was, therefore, inevitably imperfect. London experienced an early wave of infections, with cases appearing in late February 2020 and rising steeply over the following 3 months [3]. Healthcare workers (HCWs) working with COVID-19 patients have a higher infection risk than those with no patient contact [4, 5]. It is less clear whether HCWs working in non-COVID clinical environments were also at increased risk of infection. Recommendations from Public Health England (PHE) specified that HCWs working on COVID wards should wear PPE. There was no requirement to wear personal protective equipment (PPE) on non-COVID wards. We aimed to assess HCW SARS-CoV-2 infection during the initial pandemic in an acute care facility in London, with particular reference to non-COVID clinical environments. The facility has 454 acute inpatient and 120 elective and rehabilitation beds with approximately 1:4 distribution of single rooms to open 4-beded bays.

Methods

All hospital staff were invited for a voluntary blood test between May 25th-July 10th 2020, if they wished to know their SARS-CoV-2 antibody status. Samples were screened at a UKAS accredited laboratory for antibodies against SARS-CoV-2 spike protein using the Fortress COVID-19 Total Antibody ELISA kit. On attending the blood testing facility, staff were given an information sheet on the project, gave signed informed consent and were asked to complete a voluntary questionnaire detailing in which areas of the hospital they had worked between 17th February-25th May 2020, age group, ethnicity, COVID-19 patient exposure, days of sickness, SARS-CoV-2 PCR swab test results, WHO COVID-19 defined symptoms experienced during this period, symptoms in household contacts, modes of transport utilised and suggestions for improvement. The questionnaire can be found in table S1. All hospital staff who attended the phlebotomy service and completed a questionnaire, with consent were eligible. Questionnaires were available throughout the testing period. The voluntary nature of the blood testing and completion of the questionnaire meant the study sample may not have been fully representative of all hospital staff. Demographics of the staff recruited are shown in table 1 in the results section and in supplementary table S2. Evidence of SARS-CoV-2 infection was measured by the presence of positive antibodies and/or a positive PCR test. Staff were allocated a risk exposure according to their detailed working environment and grouped into non-patient facing and patient-facing environments. The latter were further subcategorised into solely non-COVID environments, mixed exposure or COVID wards. SARS-CoV-2 infection risk in non-COVID wards was measured by the numbers of patients transferred from a non-COVID to a COVID ward. The total number of weekly hospital inpatient admissions and numbers of those who were treated as COVID positive on admission were recorded. Staff blood samples were taken under the national

NHS England & Improvement voluntary healthcare staff testing programme during the initial pandemic. The Health Research Authority Decision Tool was used to confirm that National Ethics approval was not required, and the analysis was conducted as part of an internally approved service evaluation. The data were pseudonymised for analysis.

		Total<br>N=1,118 (%)	negative/indeterminate<br>N=733 (65.6%)	positive<br>N=385 (34.4%)
Age	18-30	295	191 (64.7)	104 (35.3)
	31-40	256	164 (64.1)	92 (35.9)
	41-50	260	163 (62.7)	97 (37.3)
	51-60	232	154 (66.4)	78 (33.6)
	>60	71	57 (80.3)	14 (19.7)
	unknown	4	4 (100.0)	0 (0.0)
Gender	male	233	151 (64.8)	82 (35.2)
	female	878	575 (65.5)	303 (34.5)
	unknown	7	7 (100.0)	0 (0.0)
Ethnicity	white	592	440 (74.3)	152 (25.7)
	black	118	64 (54.2)	54 (45.8)
	asian	315	171 (54.3)	144 (45.7)
	mixed	24	17 (70.8)	7 (29.2)
	other	37	19 (51.4)	18 (48.6)
	unknown	32	22 (68.8)	10 (31.2)
BAME	white/unknown	624	462 (74.0)	162 (26.0)
	BAME	494	271 (54.9)	223 (45.1)
Public transport	no	740	508 (68.6)	232 (31.4)
	yes	378	225 (59.5)	153 (40.5)
Risk allocation	Laboratory (NPF)	74	66 (89.2)	8 (10.8)
	Non-clinical hospital staff (NPF)	220	169 (76.8)	51 (23.2)
	Non-COVID wards only (PF)	286	196 (68.5)	90 (31.5)
	Mixed exposure (PF)	346	194 (56.1)	152 (43.9)
	COVID wards throughout (PF)	168	94 (56.0)	74 (44.0)

	Patient facing - Unknown	24	14 (58.3)	10 (41.7)
Symptomatic household contacts				
	No	718	517 (72.0)	201 (28.0)
	Yes	350	185 (52.9)	165 (47.1)
	Unknown	50	31 (62.0)	19 (38.0)

Table 1: Demographics of all 1118 staff by evidence of infection (positive serology or positive PCR versus negative for both tests). Values are all n (%) with p-values from chi-square tests (see supplement S1)

NPF – non patient-facing, PF – patient-facing, BAME: Black and minority ethnic

Anal[illegible]

Multiple regression models were used to estimate adjusted prevalence rates and odds ratio's between categories. Age, gender, ethnicity, mode of transport, and positive household contacts were included as potential confounders since this had been demonstrated in previous studies to be associated with infection and were potentially correlated with staff environment. Two models were estimated: i) broadly categorising staff as patient-facing versus non patient-facing; ii) more granular categories to separate patient-facing staff by time spent on COVID wards. Estimates from all models are included as supplementary material.

Results

1168 (33%) questionnaires were returned during 3520 staff blood tests. Fifty participants were excluded (5 incomplete records, 17 no blood sample, 28 prolonged home/community working). The demographic characteristics of the 1118 staff included are shown in Table 1 divided by the main outcome (evidence of infection or not). Further demographic details are in the supplementary table S2. Overall, 385 individuals (34.4%; 95%CI 31.7 to 37.3%) had evidence of SARS-CoV-2 infection (383 antibody positive and 2 swab positive but antibody negative). Of those with positive antibodies

against SARS-CoV-2, 203 (52.7%; 95% CI 47.6 to 57.8%) reported never having had a swab, despite 125 (61.6%; 95%CI 54.5 to 68.3) having experienced symptoms.

The regression analyses showed the following factors associated with an increased risk of infection: Black and minority ethnic (BAME) versus white, household contact with symptoms, and patient exposure risk (Table 2). Staff who were patient-facing versus non patient-facing were more than twice as likely to have evidence of infection (adjusted prevalence 38.6% vs 23.0%; OR 2.19; 95%CI 1.57 to 3.07). Using more granular categorisation demonstrated a stepwise increase by risk of COVID-19 patient exposure (Table 2 & Figure 1). Even those on non-COVID wards throughout had around a one-in-three chance of infection (prevalence 31.5%).

Fig 1 - Adjusted predicted probabilities of infection for the patient facing variable using different patient-facing groupings where these are adjusted for age, gender, BAME, public transport and household contacts. Non patient-facing cohort compared to all patient-facing subcategories.

		Od[illegible]atio	Confidence interval	p value
**Age**				
	18 – 30	1.0		
	31 - 40	1.10	0.763 – 1.600	0.599
	41 – 50	1.27	0.873 – 1.841	0.213
	51 – 60	1.64	1.094 – 2.457	0.017
	> 60	0.86	0.442 – 1.672	0.657
**Gender**				
	Male	1.0		
	Female	1.01	0.729 – 1.391	0.966
**Ethnicity**				
	White	1.0		
	BAME	1.89	1.422 – 2.510	<0.001
**Public transport used**				
	No	1.0		
	Yes	1.13	0.847 -1.506	0.408
**Household symptoms?**				
	No	1.0		
	Yes	2.16	1.631 – 2.854	<0.001
	Unknown	1.81	0.964 – 3.407	0.065

Risk exposure using non PF as reference	Non patient-facing	1.0		
	PF non-COVID	1.60	1.074 – 2.371	0.021
	PF mixed	2.63	1.812 – 3.836	<0.001
	PF COVID	2.61	1.682 – 4.063	<0.001

Table 2 – Multiple logistic regression of the risk of infection on selected predictors. *NPF – non patient-facing, PF – patient-facing, BAME: Black and minority ethnic*

Figure 2 shows the numbers of patients admitted to a COVID ward each week during the peak pandemic period, broken into those by direct admission or those transferred from a non-COVID ward, with a reference curve demonstrating the percentage of adult admissions that were initially coded as COVID on admission. A high number of patients on COVID wards during the first peak pandemic had initially been admitted to a non-COVID ward (22-48%). As the wave subsided, non-COVID ward transfers to COVID wards exceeded direct admissions with COVID, though numbers were small. Supplementary table 4 summarises staff comments for improvement. There were 234 replies with 261 comments. Main staff concerns were lack of reliable rapid testing, limited isolation facilities, insufficient patient separation and concern about appropriate ward allocation. Suggestions included separate hospital COVID floors and lifts, and less reliance on test results alone for ward allocation. Although staff were not asked about PPE, 9% of replies mentioned inadequacy as a concern.

Fig 2 - Breakdown of the routes of admission of adult COVID patients into a COVID ward during the initial pandemic

* Start of national UK lockdown

** Mandatory admissions testing commenced

Discussion

Evidence of increased COVID-19 in HCWs

Our data confirm previous observations that HCWs working in patient-facing environments are at increased risk of infection compared to those in non patient-facing environments, [6] and those of BAME background and with symptomatic household contacts are at higher risk (table 2). Infection risk increased with risk of COVID-19 patient exposure. Our finding of 31.5% infection rate in staff from patient-facing non-COVID environments is similar to that found in another study [7] at 28.9% and contrasts with 23.5% in non patient-facing staff.

COVID-19 patients on non-COVID wards

There were large numbers of patients who were initially admitted to a non-COVID ward and later transferred to a COVID ward (Fig 2). It is, therefore, not surprising that HCWs in patient-facing non-COVID environments were at increased risk of infection. This observation is supported by other studies that show clusters of infection outbreaks in non-COVID wards [6, 8]). Possibly, the unexpectedness of having unknown COVID status patients in non-COVID areas and reduced use of PPE may have enhanced HCW infection risk. The percentage of patients admitted to COVID wards who originated in non-COVID wards increased with time suggesting there may have been some nosocomial transmission. Nosocomial transmission has been reported to occur [9] but precise measurement is difficult because of overlap between some patients admitted with COVID displaying atypical symptoms, and those who acquired COVID after admission. The staff suggestions for improvement showed considerable concern about the difficulty in discriminating COVID from non-COVID patients and mis-classification at the time. A possible way to mitigate this is to improve decisions about patient ward moves, including adding clinical input into decisions as well as requiring negative test results. Initially swab testing capacity for patients and staff was limited as evidenced by how many symptomatic staff had not had a swab test done. Whilst testing is now more widely available and timely than in March-May 2020, tests have a significant false negative rate [2]. Thus, the problem may have diminished but still continues. PPE availability according to PHE guidance was not a significant issue in the trust but some staff were concerned that the recommended level of protection may have

been inadequate. Deficiencies detected retrospectively in this study were likely due to lack of knowledge about the virus at the time and testing and environmental constraints rather than poor professionalism. Epidemiological studies show both how infectious and how difficult it is to prevent spread of the virus within closed communities [9] [10]. Similar challenges are likely to have been replicated across the world and this study offers an opportunity to understand the scale of the challenge.

Strengths and Limitations

Strengths of this real world experience study were the large numbers of staff recruited soon after the initial wave of infection, carefully detailed staff categorisation about COVID-19 patient exposure and the high numbers of COVID-19 patients hospitalised. The combination of staff infection rates and detailed ward exposure have rarely been captured.

Limitations include possible sampling bias due to incomplete staff capture: Possibly there was a bias towards staff who regarded themselves at high risk for SARS-CoV-2 exposure. However, the seroprevalence of London blood donors in May 2020 was high at 17.5% [3] and does not contrast too much from the seroprevalence of those in non patient-facing roles at 23.2%. Also, inability to trace source of exposure (no viral fingerprinting) and, therefore, it was impossible to infer causality with certainty. External validity is limited by the fact that the pandemic is evolving, and these findings relate to experiences with a very high community case prevalence. In times of much lower case prevalence, findings would likely be very different.

Conclusion

Labelling hospital wards as COVID/non-COVID can provide a false sense of security for staff. Our data clearly show that transmission rates amongst staff are substantial even on the non-COVID wards. During periods of high community COVID prevalence and with the emergence of new more infectious

SARS-CoV-2 variants, all wards should adhere to common PPE standards if we want to minimise viral transmission in health care facilities.

Acknowledgements

We thank Nergish Desai, Head of Infection Surveillance and Dominic Thurgood, Business Intelligence Unit, King's College Hospital NHS Trust for their invaluable help with the data.

References

1. *Li, Q.; Guan, X.; Wu, P.; Wang, X.; Zhou, L.; Tong, Y.; Ren, R.; Leung, K.S.M.; Lau, E.H.Y.; Wong, J.Y.; et al. Early Transmission Dynamics in Wuhan, China, of Novel Coronavirus–Infected Pneumonia. N Engl J Med 2020; 382:1199-1207 DOI: 10.1056/NEJMoa2001316*

2. *Arevalo-Rodriguez, I.; Buitrago-Garcia, D.; Simancas-Racines, D.; Zambrano-Achig, P.; Del Campo, R.; Ciapponi, A.; Sued, O.; Martinez-García, L.; Rutjes, A.W.; Low, N.; et al. False-negative results of initial RT-PCR assays for COVID-19: A systematic review. PLoS ONE 15(12): e0242958. https://doi.org/10.1371/journal.pone.0242958*

3. *Public Health England Weekly Coronavirus Disease 2019 (COVID - 19) Surveillance Report: 23rd April 2020 (week 17); 2020; Vol. 2019;* https://www.gov.uk/government/publications/national-covid-19-surveillance-reports/sero-surveillance-of-covid-19 accessed 28 December 2020

4. *Iversen, K.; Bundgaard, H.; Hasselbalch, R.B.; Kristensen, J.H.; Nielsen, P.B.; Pries-Heje, M.; Knudsen, A.D.; Christensen, C.E.; Fogh, K.; Norsk, J.B.; et al. Risk of COVID-19 in health-care workers in Denmark: an observational cohort study. Lancet Infect. Dis. 2020, doi:10.1016/S1473-3099(20)30589-2.*

5. Nguyen, L.; Drew., D.; Graham, M.; et al. Risk of COVID-19 among front-line health-care workers and the general community: a prospective cohort study. Lancet Public Health 2020; 5: e475–83. https://doi.org/10.1016/ S2468-2667(20)30164-X

5. *Eyre, D.W.; Lumley, S.F.; O'donnell, D.; Campbell, M.; Sims, E.; Lawson, E.; Warren, F.; James, T.; Cox, S.; Howarth, A.; et al. Differential occupational risks to healthcare workers from SARS-CoV-2 observed during a prospective observational study. eLife 2020;9:e60675 DOI: 10.7554/eLife.60675*

6. *Grant, J.; Wilmore, S.; McCann, N.; Donnelly, O.; Lai, R.; Kinsella, M.; Rochford, H.; Patel, T.; Kelsey, M.; Andrews, J. Seroprevalence of SARS-CoV-2 antibodies in healthcare workers at a London NHS Trust. Infect. Control Hosp. Epidemiol. 2021 Feb;42(2):212-214. doi: 10.1017/ice.2020.402*

7. *Rivett, L.; Sridhar, S.; Sparkes, D.; Routledge, M.; Jones, N.K.; Forrest, S.; Young, J.; Pereira-Dias, J.; Hamilton, W.L.; Ferris, M.; et al. Screening of healthcare workers for SARS-CoV-2 highlights the role of asymptomatic carriage in COVID-19 transmission. eLife 2020;9:e58728 DOI: 10.7554/eLife.58728*

8. Wake, R.M.; Morgan, M.; Choi, J.; Winn, S. Reducing nosocomial transmission of COVID-19: Implementation of a COVID-19 triage system. Clin. Med. J. R. Coll. Physicians London 2020, doi:10.7861/CLINMED.2020-0411.

9. Kasper M et al, An Outbreak of Covid-19 on an Aircraft Carrier. N Engl J Med 2020; 383:2417-2426 DOI: 10.1056/NEJMoa2019375

10. Michael N.L. SARS-CoV-2 in the U.S. Military — Lessons for Civil Society**.** *N Engl J Med 2020; 383:2472-2473 DOI: 10.1056/NEJMe2032179*

Supporting information – captions

S1Table*:* Questionnaire completed by participants

S2 table: Demographics of all 1118 staff by evidence of infection (positive serology or positive PCR versus negative for both tests). Values are all n (%) with p-values from chi-square tests

NPF – non patient-facing, PF – patient-facing

S3Table*:* Demographics of all 1118 staff by role (patient-facing or non patient-facing). Values are all n (%) with p-values from chi-square tests.

S4 Table: Themes in staff responses to the question 'Do you have any suggestions for ways in which we can improve the processes for separating COVID and non-COVID patients for the future?'

*Staff were not specifically asked about PPE but these were free-text responses to the above question.

Figure 1

Click here to access/download;Figure;Fig1.tif

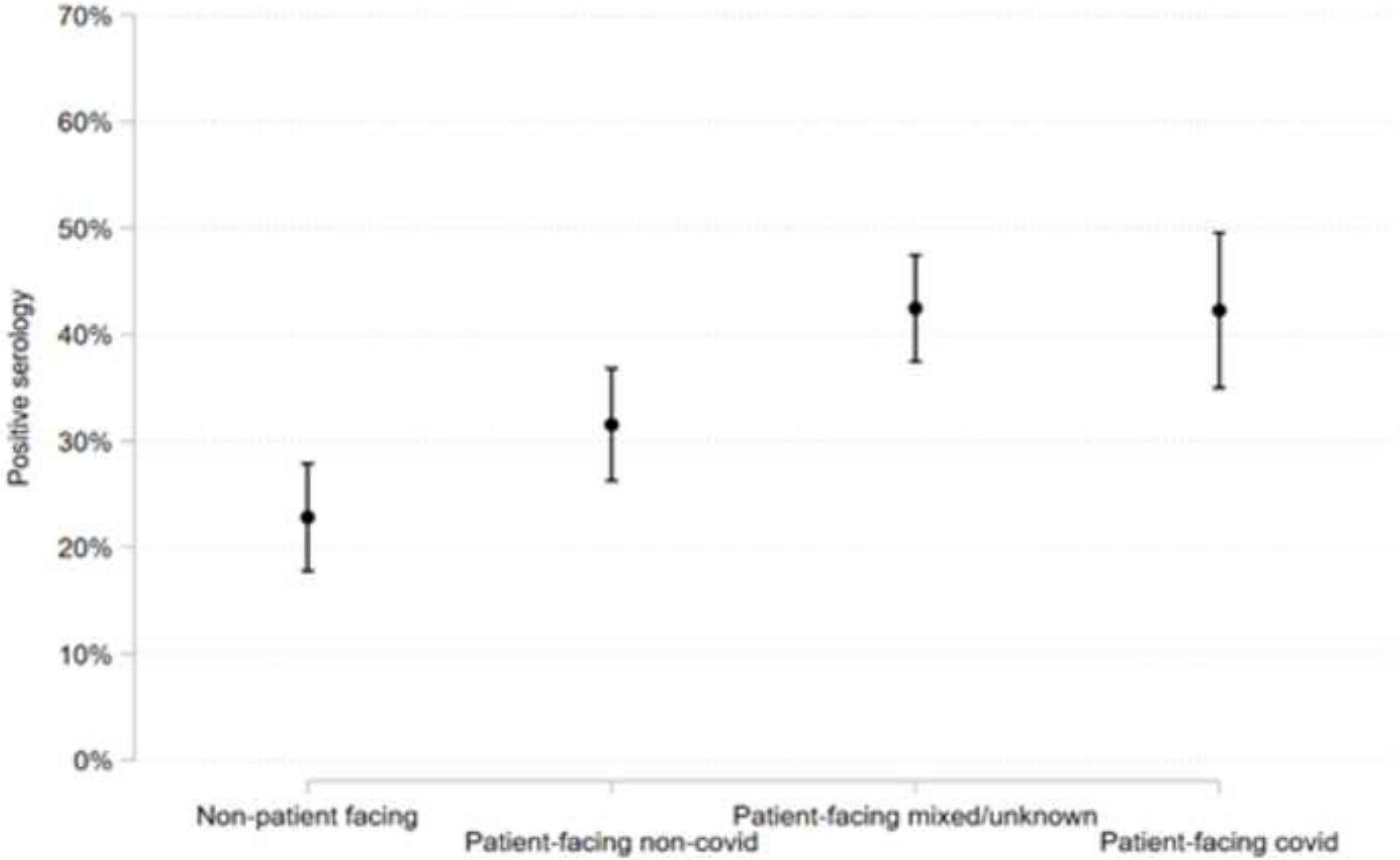

Figure 2

Click here to access/download;Figure;Fig 2.tif

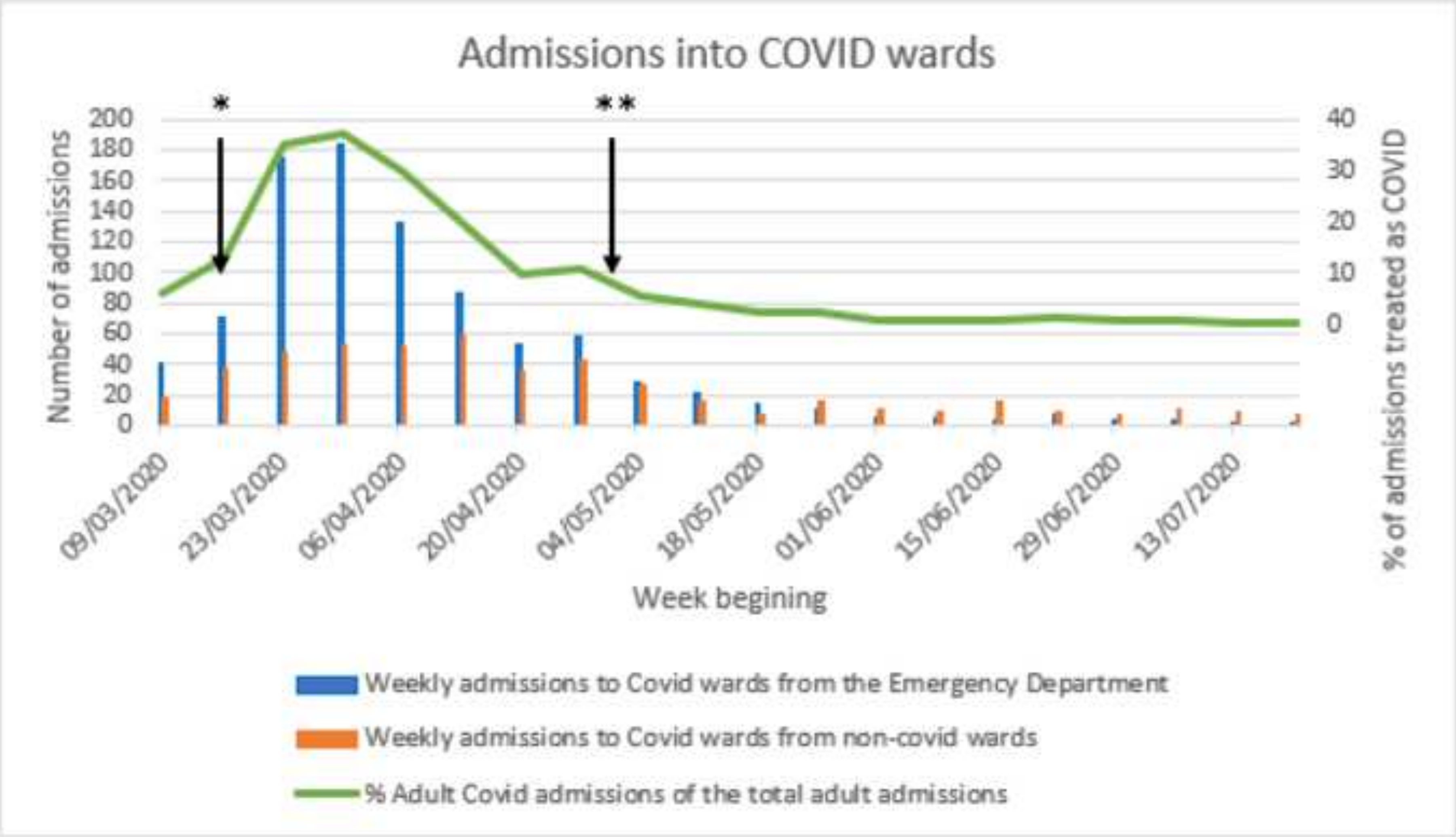

Click here to access/download
Supporting Information
Supplementary_tables_and_figures_plosone_180520.docx

Data set for reference

Click here to access/download
Supporting Information
Data set_plosone_pdf.pdf

Full title: High numbers of COVID-19 patients transit through non-COVID wards, and associated healthcare workers have high infection rates: An observational cross-sectional study

Short title: COVID-19 patients on non-COVID wards

Susanna Nallamilli[1], Tejus Patel[1], April Buazon[2], Jennifer Vidler[3], Sam Norton[4], Mustafa Atta[2], James Galloway[4] and Stella Bowcock[3]

[1] Department of Medicine, Princess Royal University Hospital, King's College Hospital NHS Trust, Kent, [2] Department of Microbiology, Princess Royal University Hospital, King's College Hospital NHS Trust, Kent, [3]Department of Haematology, Princess Royal University Hospital, King's College Hospital NHS Trust, Kent, [4]Centre for Rheumatic Disease, King's College London, London

Abstract

Infection risk is high in healthcare workers working with COVID-19 patients but the risk in non-COVID clinical environments is less clear. We measured infection rates early in the pandemic by SARS-CoV-2 antibody and/or a positive PCR test in 1118 HCWs within various hospital environments with particular focus on non-COVID clinical areas. Infection risk on non-COVID wards was estimated through the surrogate metric of numbers of patients transferred from a non-COVID to a COVID ward. Staff infection rates increased with likelihood of COVID exposure and suggested high risk in non-COVID clinical areas (non patient-facing 23.2% versus patient-facing in either non-COVID environments 31.5% or COVID wards 44%). High numbers of patients admitted to COVID wards had initially been admitted to designated non-COVID wards (22-48% at peak). Infection risk was high during a pandemic in all clinical environments and non-COVID designation may provide false reassurance. Our findings support the need for common personal protective equipment standards in all clinical areas, irrespective of COVID/non-COVID designation.

Introduction

The severe acute respiratory syndrome coronavirus 2 (SARS-CoV-2) swept the world rapidly in early 2020, before we had fully understood the transmission characteristics and illness spectrum caused by

the virus [1]. Early in the pandemic, testing capacity for the virus was limited, turnaround slow and the test was insufficiently sensitive to enable effective separation of patients at the point of admission [2]. The separation of patients into non-COVID and COVID areas was, therefore, inevitably imperfect. London experienced an early wave of infections, with cases appearing in late February 2020 and rising steeply over the following 3 months [3]. Healthcare workers (HCWs) working with COVID-19 patients have a higher infection risk than those with no patient contact [4, 5]. It is less clear whether HCWs working in non-COVID clinical environments were also at increased risk of infection. Recommendations from Public Health England (PHE) specified that HCWs working on COVID wards should wear PPE. There was no requirement to wear personal protective equipment (PPE) on non-COVID wards. We aimed to assess HCW SARS-CoV-2 infection during the initial pandemic in an acute care facility in London, with particular reference to non-COVID clinical environments. The facility has 454 acute inpatient and 120 elective and rehabilitation beds with approximately 1:4 distribution of single rooms to open 4-beded bays.

Methods

All hospital staff were invited for a voluntary blood test between May 25th-July 10th 2020, if they wished to know their SARS-CoV-2 antibody status ~~between May 25th-July 10th 2020~~. Samples were screened at a UKAS accredited laboratory for antibodies against SARS-CoV-2 spike protein using the Fortress COVID-19 Total Antibody ELISA kit. On attending the blood testing facility, staff were given an information sheet on the project, gave signed informed consent and were asked to complete a voluntary questionnaire detailing in which areas of the hospital they had worked between 17th February-25th May 2020, age group, ethnicity, COVID-19 patient exposure, days of sickness, SARS-CoV-2 PCR swab test results, WHO COVID-19 defined symptoms experienced during this period, symptoms in household contacts, modes of transport utilised and suggestions for improvement. The questionnaire can be found in table S1. All hospital staff who attended the phlebotomy service and completed a questionnaire, with consent were eligible. Questionnaires were available throughout the testing period. The voluntary nature of the blood testing and completion of the questionnaire meant the study sample may not have been fully representative of all hospital staff. Demographics of the staff recruited are shown in table 1 in the results section and in supplementary table S2. Evidence of SARS-CoV-2 infection was measured by the presence of positive antibodies and/or a positive PCR test. Staff were allocated a risk exposure according to their detailed working environment and grouped into non-patient facing and patient-facing environments. The latter were further subcategorised into solely non-COVID environments, mixed exposure or COVID wards. SARS-CoV-2 infection risk in non-COVID wards was measured by the numbers of patients transferred from a non-COVID to a COVID ward. The total number of weekly hospital inpatient admissions and numbers of those who were treated as COVID positive on admission were recorded. S~~Ethical~~

Formatted: Left, Line spacing: Multiple 1.08 li

approval for staff blood samples were takenas provided under the national NHS England & ImprovementInnovation voluntary healthcare staff testing programme -national Public Health England study of infection rates in healthcare workers dduring the initial pandemic. The analysis was conducted as part of an internally approved service evaluation. The Health Research Authority Decision Tool was used to confirm that National Ethics approval was not required, and- tThe analysis was conducted as part of an internally approved service evaluation. consent was not obtained although written informed consent was obtained from staff to use staff demographic data and access staff SARS-CoV-2 PCR and serology results. The data were pseudonymised for analysis. Our study was deemed not necessary as this was a service evaluation using secondary data and permission was granted by the hospitaltrust management board. Individual wWritten informed consent was obtained to use staff demographic data and access staff SARS-CoV-2 PCR and serology results.

Formatted: Font: (Default) Calibri, 12 pt, Font color: Black

		Total N=1,118 (%)	negative/indeterminate N=733 (65.6%)	positive N=385 (34.4%)
Age	18-30	295	191 (64.7)	104 (35.3)
	31-40	256	164 (64.1)	92 (35.9)
	41-50	260	163 (62.7)	97 (37.3)
	51-60	232	154 (66.4)	78 (33.6)
	>60	71	57 (80.3)	14 (19.7)
	unknown	4	4 (100.0)	0 (0.0)
Gender	male	233	151 (64.8)	82 (35.2)
	female	878	575 (65.5)	303 (34.5)
	unknown	7	7 (100.0)	0 (0.0)
Ethnicity	white	592	440 (74.3)	152 (25.7)
	black	118	64 (54.2)	54 (45.8)
	asian	315	171 (54.3)	144 (45.7)
	mixed	24	17 (70.8)	7 (29.2)
	other	37	19 (51.4)	18 (48.6)
	unknown	32	22 (68.8)	10 (31.2)
BAME	white/unknown	624	462 (74.0)	162 (26.0)
	BAME	494	271 (54.9)	223 (45.1)
Public transport	no	740	508 (68.6)	232 (31.4)
	yes	378	225 (59.5)	153 (40.5)
Risk allocation	Laboratory (NPF)	74	66 (89.2)	8 (10.8)
	Non-clinical hospital staff (NPF)	220	169 (76.8)	51 (23.2)
	Non-COVID wards only (PF)	286	196 (68.5)	90 (31.5)
	Mixed exposure (PF)	346	194 (56.1)	152 (43.9)
	COVID wards throughout (PF)	168	94 (56.0)	74 (44.0)

	Patient facing - Unknown	24	14 (58.3)	10 (41.7)
Symptomatic household contacts				
	No	718	517 (72.0)	201 (28.0)
	Yes	350	185 (52.9)	165 (47.1)
	Unknown	50	31 (62.0)	19 (38.0)

Table 1: Demographics of all 1118 staff by evidence of infection (positive serology or positive PCR versus negative for both tests). Values are all n (%) with p-values from chi-square tests (see supplement S1)

NPF – non patient-facing, PF – patient-facing, BAME: Black and minority ethnic

Analysis

Multiple regression models were used to estimate adjusted prevalence rates and odds ratio's between categories. Age, gender, ethnicity, mode of transport, and positive household contacts were included as potential confounders since this had been demonstrated in previous studies to be associated with infection and were potentially correlated with staff environment. Two models were estimated: i) broadly categorising staff as patient-facing versus non patient-facing; ii) more granular categories to separate patient-facing staff by time spent on COVID wards. Estimates from all models are included as supplementary material.

Results

1168 (33%) questionnaires were returned during 3520 staff blood tests. Fifty participants were excluded (5 incomplete records, 17 no blood sample, 28 prolonged home/community working). The demographic characteristics of the 1118 staff included are shown in Table 1 divided by the main outcome (evidence of infection or not). Further demographic details are in the supplementary table S2. Overall, 385 individuals (34.4%; 95%CI 31.7 to 37.3%) had evidence of SARS-CoV-2 infection (383 antibody positive and 2 swab positive but antibody negative). Of those with positive antibodies

against SARS-CoV-2, 203 (52.7%; 95% CI 47.6 to 57.8%) reported never having had a swab, despite 125 (61.6%; 95%CI 54.5 to 68.3) having experienced symptoms.

The regression analyses showed the following factors associated with an increased risk of infection: Black and minority ethnic (BAME) versus white, household contact with symptoms, and patient exposure risk (Table 2). Staff who were patient-facing versus non patient-facing were more than twice as likely to have evidence of infection (adjusted prevalence 38.6% vs 23.0%; OR 2.19; 95%CI 1.57 to 3.07). Using more granular categorisation demonstrated a stepwise increase by risk of COVID-19 patient exposure (Table 2 & Figure 1). Even those on non-COVID wards throughout had around a one-in-three chance of infection (prevalence 31.5%).

Fig 1 - Adjusted predicted probabilities of infection for the patient facing variable using different patient-facing groupings where these are adjusted for age, gender, BAME, public transport and household contacts. Non patient-facing cohort compared to all patient-facing subcategories.

		Odds ratio	Confidence interval	p value
**Age**				
	18 – 30	1.0		
	31 - 40	1.10	0.763 – 1.600	0.599
	41 – 50	1.27	0.873 – 1.841	0.213
	51 – 60	1.64	1.094 – 2.457	0.017
	> 60	0.86	0.442 – 1.672	0.657
**Gender**				
	Male	1.0		
	Female	1.01	0.729 – 1.391	0.966
**Ethnicity**				
	White	1.0		
	BAME	1.89	1.422 – 2.510	<0.001
**Public transport used**				
	No	1.0		
	Yes	1.13	0.847 -1.506	0.408
**Household symptoms?**				
	No	1.0		
	Yes	2.16	1.631 – 2.854	<0.001
	Unknown	1.81	0.964 – 3.407	0.065

Risk exposure using non PF as reference	Non patient-facing	1.0		
	PF non-COVID	1.60	1.074 – 2.371	0.021
	PF mixed	2.63	1.812 – 3.836	<0.001
	PF COVID	2.61	1.682 – 4.063	<0.001

Table 2 – Multiple logistic regression of the risk of infection on selected predictors. *NPF – non patient-facing, PF – patient-facing, BAME: Black and minority ethnic*

Figure 2 shows the numbers of patients admitted to a COVID ward each week during the peak pandemic period, broken into those by direct admission or those transferred from a non-COVID ward, with a reference curve demonstrating the percentage of adult admissions that were initially coded as COVID on admission. A high number of patients on COVID wards during the first peak pandemic had initially been admitted to a non-COVID ward (22-48%). As the wave subsided, non-COVID ward transfers to COVID wards exceeded direct admissions with COVID, though numbers were small. Supplementary table 4 summarises staff comments for improvement. There were 234 replies with 261 comments. Main staff concerns were lack of reliable rapid testing, limited isolation facilities, insufficient patient separation and concern about appropriate ward allocation. Suggestions included separate hospital COVID floors and lifts, and less reliance on test results alone for ward allocation. Although staff were not asked about PPE, 9% of replies mentioned inadequacy as a concern.

Fig 2 - Breakdown of the routes of admission of adult COVID patients into a COVID ward during the initial pandemic

* Start of national UK lockdown

** Mandatory admissions testing commenced

Discussion

Evidence of increased COVID-19 in HCWs

Our data confirm previous observations that HCWs working in patient-facing environments are at increased risk of infection compared to those in non patient-facing environments, [6] and those of BAME background and with symptomatic household contacts are at higher risk (table 2). Infection risk increased with risk of COVID-19 patient exposure. Our finding of 31.5% infection rate in staff from patient-facing non-COVID environments is similar to that found in another study [7] at 28.9% and contrasts with 23.5% in non patient-facing staff.

COVID-19 patients on non-COVID wards

There were large numbers of patients who were initially admitted to a non-COVID ward and later transferred to a COVID ward (Fig 2). It is, therefore, not surprising that HCWs in patient-facing non-COVID environments were at increased risk of infection. This observation is supported by other studies that show clusters of infection outbreaks in non-COVID wards [6, 8]). Possibly, the unexpectedness of having unknown COVID status patients in non-COVID areas and reduced use of PPE may have enhanced HCW infection risk. The percentage of patients admitted to COVID wards who originated in non-COVID wards increased with time suggesting there may have been some nosocomial transmission. Nosocomial transmission has been reported to occur [9] but precise measurement is difficult because of overlap between some patients admitted with COVID displaying atypical symptoms, and those who acquired COVID after admission. The staff suggestions for improvement showed considerable concern about the difficulty in discriminating COVID from non-COVID patients and mis-classification at the time. A possible way to mitigate this is to improve decisions about patient ward moves, including adding clinical input into decisions as well as requiring negative test results. Initially swab testing capacity for patients and staff was limited as evidenced by how many symptomatic staff had not had a swab test done. Whilst testing is now more widely available and timely than in March-May 2020, tests have a significant false negative rate [2]. Thus, the problem may have diminished but still continues. PPE availability according to PHE guidance was not a significant issue in the trust but some staff were concerned that the recommended level of protection may have

been inadequate. Deficiencies detected retrospectively in this study were likely due to lack of knowledge about the virus at the time and testing and environmental constraints rather than poor professionalism. Epidemiological studies show both how infectious and how difficult it is to prevent spread of the virus within closed communities [9] [10]. Similar challenges are likely to have been replicated across the world and this study offers an opportunity to understand the scale of the challenge.

Strengths and Limitations

Strengths of this real world experience study were the large numbers of staff recruited soon after the initial wave of infection, carefully detailed staff categorisation about COVID-19 patient exposure and the high numbers of COVID-19 patients hospitalised. The combination of staff infection rates and detailed ward exposure have rarely been captured.

Limitations include possible sampling bias due to incomplete staff capture: Possibly there was a bias towards staff who regarded themselves at high risk for SARS-CoV-2 exposure. However, the seroprevalence of London blood donors in May 2020 was high at 17.5% [3] and does not contrast too much from the seroprevalence of those in non patient-facing roles at 23.2%. Also, inability to trace source of exposure (no viral fingerprinting) and, therefore, it was impossible to infer causality with certainty. External validity is limited by the fact that the pandemic is evolving, and these findings relate to experiences with a very high community case prevalence. In times of much lower case prevalence, findings would likely be very different.

Conclusion

Labelling hospital wards as COVID/non-COVID can provide a false sense of security for staff. Our data clearly show that transmission rates amongst staff are substantial even on the non-COVID wards. During periods of high community COVID prevalence and with the emergence of new more infectious

SARS-CoV-2 variants, all wards should adhere to common PPE standards if we want to minimise viral transmission in health care facilities.

Acknowledgements

We thank Nergish Desai, Head of Infection Surveillance and Dominic Thurgood, Business Intelligence Unit, King's College Hospital NHS Trust for their invaluable help with the data.

References

1. Li, Q.; Guan, X.; Wu, P.; Wang, X.; Zhou, L.; Tong, Y.; Ren, R.; Leung, K.S.M.; Lau, E.H.Y.; Wong, J.Y.; et al. Early Transmission Dynamics in Wuhan, China, of Novel Coronavirus–Infected Pneumonia. *N Engl J Med 2020; 382:1199-1207 DOI: 10.1056/NEJMoa2001316*

2. Arevalo-Rodriguez, I.; Buitrago-Garcia, D.; Simancas-Racines, D.; Zambrano-Achig, P.; Del Campo, R.; Ciapponi, A.; Sued, O.; Martinez-García, L.; Rutjes, A.W.; Low, N.; et al. False-negative results of initial RT-PCR assays for COVID-19: A systematic review. PLoS ONE 15(12): e0242958. https://doi.org/10.1371/journal.pone.0242958

3. Public Health England Weekly Coronavirus Disease 2019 (COVID - 19) Surveillance Report: 23rd April 2020 (week 17); 2020; Vol. 2019; https://www.gov.uk/government/publications/national-covid-19-surveillance-reports/sero-surveillance-of-covid-19 accessed 28 December 2020

4. Iversen, K.; Bundgaard, H.; Hasselbalch, R.B.; Kristensen, J.H.; Nielsen, P.B.; Pries-Heje, M.; Knudsen, A.D.; Christensen, C.E.; Fogh, K.; Norsk, J.B.; et al. Risk of COVID-19 in health-care workers in Denmark: an observational cohort study. Lancet Infect. Dis. 2020, doi:10.1016/S1473-3099(20)30589-2.

5. Nguyen, L.; Drew., D.; Graham, M.; et al. Risk of COVID-19 among front-line health-care workers and the general community: a prospective cohort study. Lancet Public Health 2020; 5: e475–83. https://doi.org/10.1016/ S2468-2667(20)30164-X

5. Eyre, D.W.; Lumley, S.F.; O'donnell, D.; Campbell, M.; Sims, E.; Lawson, E.; Warren, F.; James, T.; Cox, S.; Howarth, A.; et al. Differential occupational risks to healthcare workers from SARS-CoV-2 observed during a prospective observational study. *eLife 2020;9:e60675 DOI: 10.7554/eLife.60675*

6. Grant, J.; Wilmore, S.; McCann, N.; Donnelly, O.; Lai, R.; Kinsella, M.; Rochford, H.; Patel, T.; Kelsey, M.; Andrews, J. Seroprevalence of SARS-CoV-2 antibodies in healthcare workers at a London NHS Trust. Infect. Control Hosp. Epidemiol. *2021 Feb;42(2):212-214. doi: 10.1017/ice.2020.402*

7. Rivett, L.; Sridhar, S.; Sparkes, D.; Routledge, M.; Jones, N.K.; Forrest, S.; Young, J.; Pereira-Dias, J.; Hamilton, W.L.; Ferris, M.; et al. Screening of healthcare workers for SARS-CoV-2 highlights the role of asymptomatic carriage in COVID-19 transmission. *eLife 2020;9:e58728 DOI: 10.7554/eLife.58728*

8. *Wake, R.M.; Morgan, M.; Choi, J.; Winn, S. Reducing nosocomial transmission of COVID-19: Implementation of a COVID-19 triage system. Clin. Med. J. R. Coll. Physicians London 2020, doi:10.7861/CLINMED.2020-0411.*

9. *Kasper M et al, An Outbreak of Covid-19 on an Aircraft Carrier. N Engl J Med 2020; 383:2417-2426 DOI: 10.1056/NEJMoa2019375*

10. Michael N.L. SARS-CoV-2 in the U.S. Military — Lessons for Civil Society. *N Engl J Med 2020; 383:2472-2473 DOI: 10.1056/NEJMe2032179*

Supporting information – captions

S1Table: Questionnaire completed by participants

S2 table: Demographics of all 1118 staff by evidence of infection (positive serology or positive PCR versus negative for both tests). Values are all n (%) with p-values from chi-square tests

NPF – non patient-facing, PF – patient-facing

S3Table: Demographics of all 1118 staff by role (patient-facing or non patient-facing). Values are all n (%) with p-values from chi-square tests.

S4 Table: Themes in staff responses to the question ‘Do you have any suggestions for ways in which we can improve the processes for separating COVID and non-COVID patients for the future?’

*Staff were not specifically asked about PPE but these were free-text responses to the above question.

Dr Susanna Nallamilli
Department of Medicine
Princess Royal University Hospital
King's College Hospital NHS Trust
BR6 8ND

1/6/21

Editors-in-Chief, PLOS One

RE: Response to reviewer

Thank you to the reviewer for their comments, and for consideration of this article for publication. Please find the response to the comments below.

1. Please ensure that your manuscript meets PLOS ONE's style requirements, including those for file naming.
   Response: We have checked and edited the manuscript as per the style requirements

2. Please review your reference list to ensure that it is complete and correct. If you have cited papers that have been retracted, please include the rationale for doing so in the manuscript text, or remove these references and replace them with relevant current references. Any changes to the reference list should be mentioned in the rebuttal letter that accompanies your revised manuscript. If you need to cite a retracted article, indicate the article's retracted status in the References list and also include a citation and full reference for the retraction notice.
   Response: We have updated the reference list to reflect the most recent citation for each reference.

3. Please provide additional details regarding participant consent. In the ethics statement in the Methods and online submission information, please ensure that you have specified whether consent was informed.
   Response: Please find further information now added in lines 50-53

4. Please include additional information regarding the survey or questionnaire used in the study and ensure that you have provided sufficient details that others could replicate the analyses. For instance, if you developed the survey or questionnaire as part of this study and it is not under a copyright more restrictive than CC-BY, please include a copy, in both the original language and English, as Supporting Information. If the questionnaire is published, please provide a citation to the (1) questionnaire and/or (2) original publication associated with the questionnaire.

Response: Please find the questionnaire in the new supplementary table 1 in our 'supporting information' section. The questionnaire has not been published.

5. In your Methods section, please provide additional information about the participant recruitment method and the demographic details of your participants. Please ensure you have provided sufficient details to replicate the analyses such as:
   a) the place where participants were recruited,
   b) a description of any inclusion/exclusion criteria that were applied to participant recruitment,
   c) a table of relevant demographic details,
   d) a statement as to whether your sample can be considered representative of a larger population.
   Response: Please find added information regarding this in the methods section, lines 50-56. Supplementary table 2 lists the demographic details and can be found in the 'supporting information' document uploaded.

6. We note that you have indicated that data from this study are available upon request. PLOS only allows data to be available upon request if there are legal or ethical restrictions on sharing data publicly.
   Response: As there are no ethical or legal restrictions on sharing this data set, please find an anonymised data set uploaded onto the submission portal.

7. Please upload a copy of Figure 2, to which you refer in your text on page 2 and 3. If the figure is no longer to be included as part of the submission please remove all reference to it within the text.

   Response: Please find the figure uploaded onto the submission portal

8. Please include a caption for figure 1 and 2.

   Response: Please find the captions for both figures within the manuscript, just below the paragraph in which they are initially referenced

9. Please include a copy of Table 1 which you refer to in your text on page 2.
10. Please include a copy of Table 2 which you refer to in your text on page 2.

   Response: Please find both tables within the manuscript text

11. Please include a copy of Table 3 which you refer to in your text on page 3.

Response: The table names have now been amended, and there is no Table 3 in the revised manuscript

12. Please include captions for your Supporting Information files at the end of your manuscript, and update any in-text citations to match accordingly.

    Response: Please find all the captions for the supporting information at the end of the manuscript

We are grateful for your time and consideration of this manuscript.

Please do let us know if you require any further clarification on any of the comments above.

Yours sincerely,

Susanna Nallamilli, Tejus Patel, April Buazon, Jennifer Vidler, Sam Norton, Mustafa Atta, James Galloway and Stella Bowcock
