# Supplementary material for: High numbers of COVID-19 patients transit through non-COVID wards, and associated healthcare workers have high infection rates: An observational cross-sectional study
Source: PLoS One. 2022 Oct 19;17(10):e0275154. doi: 10.1371/journal.pone.0275154 (PMC9581418; doi:10.1371/journal.pone.0275154)
Supplement: Attachment — Submitted filename: SARS-CoV-2 infecn non-covid wards.v3.1.docx [file pone.0275154.s007.docx]

**High numbers of COVID-19 patients transit through non-COVID wards, and associated healthcare workers have high infection rates: An observational cross-sectional study**

**Susanna Nallamilli^1^, Tejus Patel^1^, April Buazon^2^, Jennifer Vidler^3^, Sam Norton^4^, Mustafa Atta^2^, James Galloway^4^ and Stella Bowcock^3^**

^1^ Department of Medicine, Princess Royal University Hospital, King’s College Hospital NHS Trust, Kent, ^2^ Department of Microbiology, Princess Royal University Hospital, King’s College Hospital NHS Trust, Kent, ^3^Department of Haematology, Princess Royal University Hospital, King’s College Hospital NHS Trust, Kent, **^4^**Centre for Rheumatic Disease, King’s College London, London

*Abstract*

Infection risk is high in healthcare workers working with COVID-19 patients but the risk in non-COVID clinical environments is less clear. We measured infection rates early in the pandemic by SARS-CoV-2 antibody and/or a positive PCR test in 1118 HCWs within various hospital environments with particular focus on non-COVID clinical areas. Infection risk on non-COVID wards was estimated through the surrogate metric of numbers of patients transferred from a non-COVID to a COVID ward. Staff infection rates increased with likelihood of COVID exposure and suggested high risk in non-COVID clinical areas (non patient-facing 23.2% versus patient-facing in either non-COVID environments 31.5% or COVID wards 44%). High numbers of patients admitted to COVID wards had initially been admitted to designated non-COVID wards (22-48% at peak). Infection risk was high during a pandemic in all clinical environments and non-COVID designation may provide false reassurance. Our findings support the need for common personal protective equipment standards in all clinical areas, irrespective of COVID/non-COVID designation.

*Introduction*

The severe acute respiratory syndrome coronavirus 2 (SARS-CoV-2) swept the world rapidly in early 2020, before we had fully understood the transmission characteristics and illness spectrum caused by the virus [1]. Early in the pandemic, testing capacity for the virus was limited, turnaround slow and the test was insufficiently sensitive to enable effective separation of patients at the point of admission [2]. The separation of patients into non-COVID and COVID areas was, therefore, inevitably imperfect. London experienced an early wave of infections, with cases appearing in late February 2020 and rising steeply over the following 3 months [3]. Healthcare workers (HCWs) working with COVID-19 patients have a higher infection risk than those with no patient contact [4, 5] . It is less clear whether HCWs working in non-COVID clinical environments were also at increased risk of infection. Recommendations from Public Health England (PHE) specified that HCWs working on COVID wards should wear PPE. There was no requirement to wear personal protective equipment (PPE) on non-COVID wards. We aimed to assess HCW SARS-CoV-2 infection during the initial pandemic in an acute care facility in London, with particular reference to non-COVID clinical environments. The facility has 454 acute inpatient and 120 elective and rehabilitation beds with approximately 1:4 distribution of single rooms to open 4-beded bays.

*Methods*

All staff were invited for a blood test between May 25^th^-July 10^th^ 2020. Samples were screened at a UKAS accredited laboratory for antibodies against SARS-CoV-2 spike protein using the Fortress COVID-19 Total Antibody ELISA kit. During blood testing staff completed a voluntary questionnaire detailing in which areas of the hospital they had worked between 17^th^ February-25^th^ May 2020, age group, ethnicity, COVID-19 patient exposure, days of sickness, SARS-CoV-2 PCR swab test results, WHO COVID-19 defined symptoms experienced during this period, symptoms in household contacts, modes of transport utilised and suggestions for improvement. Evidence of SARS-CoV-2 infection was measured by the presence of positive antibodies and/or a positive PCR test. Staff were allocated a risk exposure according to their detailed working environment and grouped into non-patient facing and patient-facing environments. The latter were further subcategorised into solely non-COVID environments, mixed exposure or COVID wards. SARS-CoV-2 infection risk in non-COVID wards was measured by the numbers of patients transferred from a non-COVID to a COVID ward. The total number of weekly hospital inpatient admissions and numbers of those who were treated as COVID positive on admission were recorded. Ethical approval was not necessary as this was a service evaluation and permission was granted by the trust management board. Individual written consent was obtained to access staff SARS-CoV-2 PCR and serology results.

*Analysis*

Multiple regression models were used to estimate adjusted prevalence rates and odds ratio’s between categories. Age, gender, ethnicity, mode of transport, and positive household contacts were included as potential confounders since this had been demonstrated in previous studies to be associated with infection and were potentially correlated with staff environment. Two models were estimated: i) broadly categorising staff as patient-facing versus non patient-facing; ii) more granular categories to separate patient-facing staff by time spent on COVID wards. Estimates from all models are included as supplementary material.

*Results*

1168 (33%) questionnaires were returned during 3520 staff blood tests. Fifty participants were excluded (5 incomplete records, 17 no blood sample, 28 prolonged home/community working). The demographic characteristics of the 1118 staff included are shown in Table 1 divided by the main outcome (evidence of infection or not). Further demographic details are in the supplementary table S1. Overall, 385 individuals (34.4%; 95%CI 31.7 to 37.3%) had evidence of SARS-CoV-2 infection (383 antibody positive and 2 swab positive but antibody negative). Of those with positive antibodies against SARS-CoV-2, 203 (52.7%; 95% CI 47.6 to 57.8%) reported never having had a swab, despite 125 (61.6%; 95%CI 54.5 to 68.3) having experienced symptoms.

The regression analyses showed the following factors associated with an increased risk of infection: Black and minority ethnic (BAME) versus white, household contact with symptoms, and patient exposure risk (Table 2). Staff who were patient-facing versus non patient-facing were more than twice as likely to have evidence of infection (adjusted prevalence 38.6% vs 23.0%; OR 2.19; 95%CI 1.57 to 3.07). Using more granular categorisation demonstrated a stepwise increase by risk of COVID-19 patient exposure (Table 2 & Figure 1). Even those on non-COVID wards throughout had around a one-in-three chance of infection (prevalence 31.5%).

Figure 2 shows the numbers of patients admitted to a COVID ward each week during the peak pandemic period, broken into those by direct admission or those transferred from a non-COVID ward, with a reference curve demonstrating the percentage of adult admissions that were initially coded as COVID on admission. A high number of patients on COVID wards during the first peak pandemic had initially been admitted to a non-COVID ward (22-48%). As the wave subsided, non-COVID ward transfers to COVID wards exceeded direct admissions with COVID, though numbers were small. Table 3 summarises staff comments for improvement. There were 234 replies with 261 comments. Main staff concerns were lack of reliable rapid testing, limited isolation facilities, insufficient patient separation and concern about appropriate ward allocation. Suggestions included separate hospital COVID floors and lifts, and less reliance on test results alone for ward allocation. Although not asked about PPE, 9% of replies mentioned inadequacy as a concern.

*Discussion*

Evidence of increased COVID-19 in HCWs

Our data confirm previous observations that HCWs working in patient-facing environments are at increased risk of infection compared to those in non patient-facing environments [6] and those of BAME background and with symptomatic household contacts are at higher risk (table 2). Infection risk increased with risk of COVID-19 patient exposure. Our finding of 31.5% infection rate in staff from patient-facing non-COVID environments is similar to that found in another study [7] at 28.9% and contrasts with 23.5% in non patient-facing staff.

COVID-19 patients on non-COVID wards

There were large numbers of patients who were initially admitted to a non-COVID ward and later transferred to a COVID ward (Fig 2). It is, therefore, not surprising that HCWs in patient-facing non-COVID environments were at increased risk of infection. This observation is supported by other studies that show clusters of infection outbreaks in non-COVID wards [6, 8]). Possibly, the unexpectedness of having unknown COVID status patients in non-COVID areas and reduced use of PPE may have enhanced HCW infection risk. The percentage of patients admitted to COVID wards who originated in non-COVID wards increased with time suggesting there may have been some nosocomial transmission. Nosocomial transmission has been reported to occur [9] but precise measurement is difficult because of overlap between some patients admitted with COVID displaying atypical symptoms, and those who acquired COVID after admission. The staff suggestions for improvement showed considerable concern about the difficulty in discriminating COVID from non-COVID patients and mis-classification at the time. A possible way to mitigate this is to improve decisions about patient ward moves, including adding clinical input into decisions as well as requiring negative test results. Initially swab testing capacity for patients and staff was limited as evidenced by how many symptomatic staff had not had a swab test done. Whilst testing is now more widely available and timely than in March-May 2020, tests have a significant false negative rate [2]. Thus, the problem may have diminished but still continues. PPE availability according to PHE guidance was not a significant issue in the trust but some staff were concerned that the recommended level of protection may have been inadequate. Deficiencies detected retrospectively in this study were likely due to lack of knowledge about the virus at the time and testing and environmental constraints rather than poor professionalism. Epidemiological studies show both how infectious and how difficult it is to prevent spread of the virus within closed communities [9] [10]. Similar challenges are likely to have been replicated across the world and this study offers an opportunity to understand the scale of the challenge.

Strengths and Limitations

Strengths of this real world experience study were the large numbers of staff recruited soon after the initial wave of infection, carefully detailed staff categorisation about COVID-19 patient exposure and the high numbers of COVID-19 patients hospitalised. The combination of staff infection rates and detailed ward exposure have rarely been captured.

Limitations include possible sampling bias due to incomplete staff capture. However, the seroprevalence of London blood donors in May 2020 was high at 17.5% [3] and does not contrast too much from the seroprevalence of those in non patient-facing roles at 23.2%. Also, inability to trace source of exposure (no viral fingerprinting) and, therefore, it was impossible to infer causality with certainty. External validity is limited by the fact that the pandemic is evolving, and these findings relate to experiences with a very high community case prevalence. In times of much lower case prevalence, findings would likely be different.

*Conclusion*

Labelling hospital wards as COVID/non-COVID can provide a false sense of security for staff. Our data clearly show that transmission rates amongst staff are substantial even on the non-COVID wards. During periods of high community COVID prevalence and with the emergence of new more infectious SARS-CoV-2 variants, all wards should adhere to common PPE standards if we want to minimise viral transmission in health care facilities.

*Acknowledgements*

We thank Nergish Desai, Head of Infection Surveillance and Dominic Thurgood, Business Intelligence Unit, King’s College Hospital NHS Trust for their invaluable help with the data.

*References*

*1.	Li, Q.; Guan, X.; Wu, P.; Wang, X.; Zhou, L.; Tong, Y.; Ren, R.; Leung, K.S.M.; Lau, E.H.Y.; Wong, J.Y.; et al. Early Transmission Dynamics in Wuhan, China, of Novel Coronavirus–Infected Pneumonia. N. Engl. J. Med. 2020, doi:10.1056/nejmoa2001316.*

*2.	Arevalo-Rodriguez, I.; Buitrago-Garcia, D.; Simancas-Racines, D.; Zambrano-Achig, P.; Del Campo, R.; Ciapponi, A.; Sued, O.; Martinez-García, L.; Rutjes, A.W.; Low, N.; et al. False-negative results of initial RT-PCR assays for COVID-19: A systematic review. PLoS One 2020, 15, e0242958, doi:10.1371/journal.pone.0242958*

*3.	Public Health England Weekly Coronavirus Disease 2019 ( COVID - 19 ) Surveillance Report: 23rd April 2020 (week 17); 2020; Vol. 2019;* <https://www.gov.uk/government/publications/national-covid-19-surveillance-reports/sero-surveillance-of-covid-19> accessed 28 December 2020

*4.	Iversen, K.; Bundgaard, H.; Hasselbalch, R.B.; Kristensen, J.H.; Nielsen, P.B.; Pries-Heje, M.; Knudsen, A.D.; Christensen, C.E.; Fogh, K.; Norsk, J.B.; et al. Risk of COVID-19 in health-care workers in Denmark: an observational cohort study. Lancet Infect. Dis. 2020, doi:10.1016/S1473-3099(20)30589-2.*

*5.* Nguyen, L.; Drew., D.; Graham, M.; et al. Risk of COVID-19 among front-line health-care workers and the general community: a prospective cohort study. Lancet Public Health 2020; 5: e475–83. https://doi.org/10.1016/ S2468-2667(20)30164-X

*5.	Eyre, D.W.; Lumley, S.F.; O’donnell, D.; Campbell, M.; Sims, E.; Lawson, E.; Warren, F.; James, T.; Cox, S.; Howarth, A.; et al. Differential occupational risks to healthcare workers from SARS-CoV-2 observed during a prospective observational study. Elife 2020, doi:10.7554/ELIFE.60675.*

*6.	Grant, J.; Wilmore, S.; McCann, N.; Donnelly, O.; Lai, R.; Kinsella, M.; Rochford, H.; Patel, T.; Kelsey, M.; Andrews, J. Seroprevalence of SARS-CoV-2 antibodies in healthcare workers at a London NHS Trust. Infect. Control Hosp. Epidemiol. 2020, doi:10.1017/ice.2020.402.*

*7.	Rivett, L.; Sridhar, S.; Sparkes, D.; Routledge, M.; Jones, N.K.; Forrest, S.; Young, J.; Pereira-Dias, J.; Hamilton, W.L.; Ferris, M.; et al. Screening of healthcare workers for SARS-CoV-2 highlights the role of asymptomatic carriage in COVID-19 transmission. Elife 2020, doi:10.7554/eLife.58728.*

*8.	Wake, R.M.; Morgan, M.; Choi, J.; Winn, S. Reducing nosocomial transmission of COVID-19: Implementation of a COVID-19 triage system. Clin. Med. J. R. Coll. Physicians London 2020, doi:10.7861/CLINMED.2020-0411.*

*9.	Kasper M et al, An Outbreak of Covid-19 on an Aircraft Carrier. November 11, 2020
DOI: 10.1056/NEJMoa2019375*

*10.*	***Michael N.L. SARS-CoV-2 in the U.S. Military — Lessons for Civil Society. November 11, 2020
DOI: 10.1056/NEJMe2032179***
