# Supplementary material for: High numbers of COVID-19 patients transit through non-COVID wards, and associated healthcare workers have high infection rates: An observational cross-sectional study
Source: PLoS One. 2022 Oct 19;17(10):e0275154. doi: 10.1371/journal.pone.0275154 (PMC9581418; doi:10.1371/journal.pone.0275154)
Supplement: Attachment — Submitted filename: Response to reviewers.docx [file pone.0275154.s008.docx]

Dr Susanna Nallamilli

Department of Medicine

Princess Royal University Hospital

King’s College Hospital NHS Trust

BR6 8ND

1/6/21

Editors-in-Chief, PLOS One

RE: Response to reviewer

Thank you to the reviewer for their comments, and for consideration of this article for publication. Please find the response to the comments below.

1. Please ensure that your manuscript meets PLOS ONE's style requirements, including those for file naming.

Response: We have checked and edited the manuscript as per the style requirements

2. Please review your reference list to ensure that it is complete and correct. If you have cited papers that have been retracted, please include the rationale for doing so in the manuscript text, or remove these references and replace them with relevant current references. Any changes to the reference list should be mentioned in the rebuttal letter that accompanies your revised manuscript. If you need to cite a retracted article, indicate the article’s retracted status in the References list and also include a citation and full reference for the retraction notice.

Response: We have updated the reference list to reflect the most recent citation for each reference.

3. Please provide additional details regarding participant consent. In the ethics statement in the Methods and online submission information, please ensure that you have specified whether consent was informed.

Response: Please find further information now added in lines 50-53

4. Please include additional information regarding the survey or questionnaire used in the study and ensure that you have provided sufficient details that others could replicate the analyses. For instance, if you developed the survey or questionnaire as part of this study and it is not under a copyright more restrictive than CC-BY, please include a copy, in both the original language and English, as Supporting Information. If the questionnaire is published, please provide a citation to the (1) questionnaire and/or (2) original publication associated with the questionnaire.

Response: Please find the questionnaire in the new supplementary table 1 in our ‘supporting information’ section. The questionnaire has not been published.

5. In your Methods section, please provide additional information about the participant recruitment method and the demographic details of your participants. Please ensure you have provided sufficient details to replicate the analyses such as:
   a) the place where participants were recruited,
   b) a description of any inclusion/exclusion criteria that were applied to participant recruitment,
   c) a table of relevant demographic details,
   d) a statement as to whether your sample can be considered representative of a larger population.

Response: Please find added information regarding this in the methods section, lines 50-56. Supplementary table 2 lists the demographic details and can be found in the ‘supporting information’ document uploaded.

6. We note that you have indicated that data from this study are available upon request. PLOS only allows data to be available upon request if there are legal or ethical restrictions on sharing data publicly.

Response: As there are no ethical or legal restrictions on sharing this data set, please find an anonymised data set uploaded onto the submission portal.

7. Please upload a copy of Figure 2, to which you refer in your text on page 2 and 3. If the figure is no longer to be included as part of the submission please remove all reference to it within the text.

Response: Please find the figure uploaded onto the submission portal

8. Please include a caption for figure 1 and 2.

Response: Please find the captions for both figures within the manuscript, just below the paragraph in which they are initially referenced

9. Please include a copy of Table 1 which you refer to in your text on page 2.
10. Please include a copy of Table 2 which you refer to in your text on page 2.

Response: Please find both tables within the manuscript text

11. Please include a copy of Table 3 which you refer to in your text on page 3.

Response: The table names have now been amended, and there is no Table 3 in the revised manuscript

12. Please include captions for your Supporting Information files at the end of your manuscript, and update any in-text citations to match accordingly.

Response: Please find all the captions for the supporting information at the end of the manuscript

We are grateful for your time and consideration of this manuscript.

Please do let us know if you require any further clarification on any of the comments above.

Yours sincerely,

Susanna Nallamilli, Tejus Patel, April Buazon, Jennifer Vidler, Sam Norton, Mustafa Atta, James Galloway and Stella Bowcock
