# Supplementary material for: High numbers of COVID-19 patients transit through non-COVID wards, and associated healthcare workers have high infection rates: An observational cross-sectional study
Source: PLoS One. 2022 Oct 19;17(10):e0275154. doi: 10.1371/journal.pone.0275154 (PMC9581418; doi:10.1371/journal.pone.0275154)
Supplement: Attachment — Submitted filename: rebuttal lett 2.Plos one.docx [file pone.0275154.s011.docx]

Dear Editor

Re: Response to reviewers PONE-D-21-07362R2

Thank you for giving us the opportunity to revise this manuscript. We have addressed all the points made by the reviewers.

1. Figures 1 and 2 which were blurred have now been uploaded in a different format and are now clearer.
2. The methods section has now been broken up into sub-sections as suggested by the reviewer. The text has not changed substantially but has been rearranged. The questionnaire has been uploaded in the supplementary material. Detail about the statistical tool used and the approach to the multivariate analysis has been added in the ‘Analysis’ section.
3. All changes to the tables as highlighted in the attached document, have been completed.

We now hope that our manuscript fully meets your requirements.
